# Immunomodulatory effects and mechanisms of Qi-Xu-Tiao-Ti formula in Qi-deficiency constitution: a randomized controlled trial integrated with multi-omics and network pharmacology analysis

**DOI:** 10.3389/fimmu.2025.1675502

**Published:** 2025-10-31

**Authors:** Renyun Cui, Ming Fan, Chengzhi Yang, Cheng Chen, Jinlu Xia, Xi Liu, Guohui Zhang, Fangli Li

**Affiliations:** ^1^ Shenzhen Hospital, Beijing University of Chinese Medicine, Shenzhen, China; ^2^ Dongzhimen Hospital, Beijing University of Chinese Medicine, Beijing, China; ^3^ Corporate Culture Department, China State Construction International Holdings Limited, Hong Kong, Hong Kong SAR, China

**Keywords:** Qi-deficiency constitution, immune function, Qi-Xu-Tiao-Ti formula, clinical trial, network pharmacology

## Abstract

**Background:**

The Qi-deficiency constitution (QDC) is a body constitution type characterized by weakened immune function and increased susceptibility to infectious diseases. Emerging evidence indicates that Chinese herbal medicine can effectively improve immunological competence in individuals with QDC. This study aims to systematically evaluate the clinical efficacy of the Qi-Xu-Tiao-Ti Formula (QXTTF) in alleviating QDC and elucidate its underlying pharmacological mechanisms through transcriptome sequencing and network pharmacology approaches.

**Methods:**

This single-center randomized controlled trial was performed using QXTTF to treat the QDC subjects, with Balanced constitution (BC) as the healthy controls. Primary outcome measures focused on traditional Chinese medicine constitution assessment of QDC scores, with secondary evaluation parameters including immune biomarkers (IgA and IgG levels) and adverse events monitoring. To investigate the molecular mechanisms, we performed transcriptomic profiling to identify QXTTF-responsive gene targets, followed by integrated network pharmacology analysis, molecular docking simulations to characterize the compound-target interactions underlying QXTTF’s therapeutic effects, and ultimately validated at the proteomics level.

**Results:**

This study recruited 78 participants, comprising 63 QDC and 15 BC individuals. Comparative analysis between QDC and BC groups revealed significant differences in immunoglobulin IgA, IgG levels, and transcriptomic profiles. QXTTF intervention on QDC subjects significantly reduced the QDC scores (*p <* 0.05) and enhanced IgA and IgG production (*p <* 0.01), indicating QXTTF’s dual efficacy in constitution regulation and potential immunomodulation. Transcriptome sequencing identified eight putative therapeutic targets: *ATP2A1*, *GOT1*, *SLC16A8*, *FTCD*, *ERBB2*, *GSS*, *VLDLR*, and *IL2*. Functional enrichment analysis revealed significant overrepresentation of differentially expressed genes in immune-related pathways, notably “natural killer cell-mediated cytotoxicity”, “T cell receptor signaling pathway”, and “B cell receptor signaling pathway”. Molecular docking simulations confirmed stable binding conformations between QXTTF’s potential active components (luteolin, quercetin, naringenin) and key targets *ERBB2*, *GOT1*, and *IL2*. Proteomic profiling unveiled statistically significant correlations (*p <* 0.05) between the expression levels of core immune pathway-associated proteins and putative therapeutic targets.

**Conclusions:**

Our study demonstrates that QXTTF potentially mitigates QDC via immunomodulatory mechanisms. with therapeutic targets systematically identified through network pharmacology analysis and subsequently validated by molecular docking and proteomic profiling. These insights are crucial in delineating the pharmacological mechanisms of Chinese herbal formulas that augment the immune function of QDC, thus establishing a scientific basis for the early prevention and management of immune-related diseases.

**Clinical trial registration:**

http://www.chictr.org.cn/, identifier ChiCTR2200063044.

## Introduction

The Constitution describes the integrated morphological structure, physiological processes, and psychological state of an individual. It arises from the interplay of inherent endowment and acquired factors, influencing an individual’s susceptibility to certain diseases (1). Academician Wang Qi classified constitutions into nine basic categories: the balanced constitution (BC), the qi-deficiency constitution (QDC), the yin-deficiency constitution (YIDC), the yang-deficiency constitution (YADC), the blood stasis constitution (BSC), the qi stagnation constitution (QSC), the dampness-heat constitution (DHC), the phlegm-dampness constitution (PDC), and the inherited special constitution (ISC). Among these constitutions, the BC represents the healthy constitution, whereas the other eight are considered biased constitutions. QDC manifests as a pathological state characterized by primordial qi (Yuan qi) insufficiency, wherein diminished vital energy reserves precipitate systemic hypofunctionality across both the organismal and visceral systems, culminating in compromised immunocompetence (2). The epidemiological study results indicated that QDC was the predominant type (53.9%) among individuals with biased constitution, and it ranked as the second most common type among Chinese individuals with biased constitution (3, 4). The previous study indicates that a variety of disease processes are associated with qi deficiency, especially stroke, diabetes, infectious diseases, acquired immunodeficiency syndrome, and hypertension (5). Recent research demonstrates that QDC correlates with psychiatric symptoms, such as depression and obsessive-compulsion (6, 7). Therefore, it is crucial to find methods to intervene in the QDC.

The constitution is a relatively stable body condition with predefined susceptibilities to certain diseases. Adjusting an individual’s body constitution makes it possible to prevent disease onset and block disease progression (8). The Qi-Xu-Tiao-Ti Formula (QXTTF) was formulated by Academician Wang Qi, grounded in his clinical expertise in regulating the QDC through immune enhancement strategies. QXTTF comprises nine Chinese herbs: Lonicerae japonicae Flos. (Chinese: Jinyinhua), Phragmitis Rhizoma (Chinese: Lugen), Imperatae Rhizoma (Chinese: Baimaogen), Pogostemonis Herba (Chinese: Huoxiang): Angelicae dahuricae Radix (Chinese: Baizhi), Citri Reticulatae Pericarpium (Chinese: Chenpi), Ginseng Radix et Rhizoma (Chinese: Renshen), Dioscoreae rhizoma (Chinese: Shanyao), and Glycyrrhizae Radix et Rhizoma (Chinese: Gancao). The clinical application of QXTTF has definitely improved the therapeutic effect of QDC. Research indicates that genes specific for QDC and targeted by its particular lncRNAs are intricately linked to immune regulation (9). However, the precise therapeutic targets of QXTTF in QDC treatment and its underlying mechanisms, particularly the immune-related pathways, remain largely poorly understood.

To address this gap, we conducted a randomized controlled trial (RCT) to evaluate the therapeutic impact of QXTTF in modulating QDC, employing multidimensional outcome assessments. Subsequently, we performed transcriptome and proteomic sequencing and systematically identified putative molecular targets of QXTTF in QDC regulation by integrating network pharmacology and molecular docking approaches, thereby elucidating the underlying pharmacological mechanisms of QXTTF.

## Materials and methods

### Study design

This study constitutes a single-center randomized controlled trial (RCT) conducted at Shenzhen Hospital of Beijing University of Chinese Medicine, located in Longgang District, Shenzhen, Guangdong Province, China. The investigation spanned from July 2023 to May 2024. This trial was approved by the Ethics Committee of Shenzhen Hospital of Beijing University of Chinese Medicine (Approval No. SZLDH2022LSYM-007), and had been registered at the ClinicalTrials.gov (Identifier: ChiCTR2200063044, http://www.chictr.org.cn/).

A simple randomization protocol was implemented utilizing a computer-generated random number table (random seed = 20230701). Participants were allocated to either the QDC-control or QDC-QXTTF group at a 1:1 ratio. Allocation sequences were secured within opaque, consecutively numbered envelopes. These envelopes were opened by an independent researcher not involved in outcome assessment. Due to the inherent characteristics of the herbal tea intervention (distinct taste and appearance), a double-blind design was not feasible. However, outcome assessors and data analysts were blinded to group assignments throughout the study.

### Participants

The written informed consent of each participant was obtained from the participant or his/her representative prior to any procedure. During the study, the participants could withdraw their consent at any time without any consequences. A total of 78 participants were enrolled in our study, comprising 15 BC and 63 QDC participants. The QDC participants were randomly categorized into QDC-control (pre-intervention QDC-control and post-intervention QDC-control) and QDC-QXTTF (pre-intervention QDC-QXTTF and post-intervention QDC-QXTTF) groups.

Inclusion criteria for participants included: (1) men and women aged 18 to 60 years old; (2) the patients had a BC or a QDC after physical identification by professional Chinese medicine practitioners. Exclusion criteria included: (1) participants with several systemic diseases (malignant tumors, autoimmune diseases, liver and kidney diseases) or had undergone surgery (splenectomy, organ transplantation); (2) pregnant or lactating women; (3) participants who participated in other clinical trials within 3 months; (4) the participants were allergic to the research drugs; (5) the participants with other constitution; (6) other situations as judged by the investigator. Exiting criteria included: (1) serious adverse drug reactions; (2) severe systemic disease during drug interventions; (3) pregnancy during drug interventions; (4) participants did not take medicine on time; (5) use of other drugs during drug interventions.

### Intervention

QXTTF comprises nine herbal components, sourced exclusively from certified suppliers and authenticated by the Department of Pharmacognosy, Shenzhen Hospital of Beijing University of Chinese Medicine. Voucher specimens are deposited within the hospital’s herbarium. All Chinese herbal medicines comply rigorously with national standards and have undergone verification by the hospital’s pharmacists (**Supplementary Figure S1-S9**). According to the composition of herbs in QXTTF, the corresponding herbs were weighted and chopped into coarse powder, and then prepared into medicinal tea according to the specific prescription ratio (Jinyinhua : Lugen : Baimaogen : Huoxiang : Baizhi : Chenpi : Renshen : Shanyao : Gancao = 6:15:15:6:6:6:5:10:3). The weight of each packet of the medicinal tea is 10 g. The participants in the QDC-QXTTF group were given one packet of the medicinal tea daily, brewed repeatedly, five days a week for 12 weeks.

### Outcome measures

In our study, the primary outcome was the Chinese medicine constitution score. The Wang Qi’s Nine Types of Traditional Chinese Medicine Constitution Scale (2009) was applied to conduct the constitution score. The scale contains a total of 67 items, each of which has a 5-level answer, and gives a score of 1–5 points from nothing to something tendency (where the item marked with * is a reverse scoring item). Select by single-choice method, and then a simple summation method was applied for the original points of each type. The calculated formulas were: original score = $\sum_{i=1}^{n=67}x_{i}$ ; and conversion score = [(original score - the number of items)/(the number of items × 4)] × 100. The determination criteria are displayed in **Table 1**.

**Table 1:** The identification criteria of the body constitution.

Constitution types	Determination criteria	Results
Balanced constitution	conversion score ≥ 60	Yes
	conversion score of each constitution in other eight constitution < 30	
	conversion score ≥ 60	Basically determined to be BC
	conversion score of each constitution in other eight constitution < 40	
	Failure to meet the above conditions	No
Biased constitution	conversion score ≥ 40	Yes
	30 ≤ conversion score < 39	Tendency
	conversion score < 30	No

Secondary outcome measures comprised immunological parameters, specifically measuring serum immunoglobulin A (IgA) and immunoglobulin G (IgG) levels. Additionally, all adverse events were systematically documented throughout the study duration.

### Transcriptome sequencing

The transcriptome sequencing was conducted at the Beijing Genomics Institution (Shenzhen, China). After extracting total RNA from blood samples, mRNA was isolated from them using mRNA enrichment, and the obtained mRNA was reverse transcribed to cDNA. Next, the cDNA was constructed into a sequencing library through a series of enzymatic reactions. After the library was developed, high-throughput sequencing was carried out, and the resulting sequencing data was called raw reads or raw data. Subsequently, quality control (QC) of raw reads was conducted using SOAPnuke (version 2.3) to ensure that sequencing data were suitable for subsequent analysis (10). After the QC was passed, clean reads were aligned to the reference genome (GCF_000001405.39_GRCh38.p13) using HISAT2 (version 2.0.4) (11). A second QC was conducted by counting the matching rate and the distribution of reads on the reference sequence to determine whether the results met the analysis requirements.

### Differentially expressed analysis

The “limma” R package (version 3.54.2) (12) was applied to identify differentially expressed genes (DEGs) between pre-intervention QDC-control and BC groups, between post-intervention QDC-control and BC groups, as well as post-intervention QDC-QXTTF and post-intervention QDC-control groups, which were denoted as DEGs1, DEGs2, and DEGs3, respectively. The screening threshold was *P*-value < 0.05. Moreover, volcano diagrams were drawn to visualize these DEGs.

### Functional enrichment analysis

To understand the potential molecular functions and signaling pathways associated with DEGs, Gene Ontology (GO) and Kyoto Encyclopedia of Genes and Genomes (KEGG) enrichment analyses were conducted by the “clusterProfiler” R package (version 4.7.1.001) (13). The GO enrichment analysis includes three parts: biological process (BP), cellular component (CC), and molecular function (MF). The item or signaling pathway with *P* less than 0.05 was considered statistically significant.

### Network pharmacology analysis

Through the Traditional Chinese Medicine Systems Pharmacology (TCMSP) (https://old.tcmsp-e.com/tcmsp.php), the potential active components corresponding to nine traditional Chinese medicines were retrieved (screening thresholds: oral bioavailability (OB) (%) ≥ 30%, drug-likeness (DL) ≥ 0.18), and the target genes of potential active components were retrieved. Moreover, the target genes of nine traditional Chinese medicines were also retrieved from The Encyclopedia of Traditional Chinese Medicine (ETCM) (http://39.98.88.176/ETCM/index.php/Home/Index/index.html). After eliminating duplication in these target genes from two databases, QXTTF-target genes were obtained. Subsequently, the down-regulated genes in DEGs2 intersected with the up-regulated genes in DEGs3, and the up-regulated genes in DEGs2 intersected with the down-regulated genes in DEGs3. Moreover, the shared genes obtained from the intersection were denoted as QDC-target genes. After that, the intersection between QXTTF-target genes and QDC-target genes was conducted to obtain potential therapeutic targets for QXTTF in treating QDC, which were denoted as key genes. The Cytoscape (version 3.7.1) was employed to visualize the drug-key genes-diseases network. Additionally, to investigate whether there were interactions among these key genes at the protein level, the STRING database (http://string.embl.de/) was applied to develop the protein-protein interaction (PPI) network (threshold setting: confidence score ≥ 0.4).

### Gene set enrichment analysis

To investigate the possible mechanisms by which QXTTF targeted key genes for the treatment of QDC, GSEA was conducted with the KEGG gene set as the reference set by the “clusterProfiler” R package (version 4.7.1.001). The correlations between each key gene and all genes were evaluated and ranked according to the correlation coefficient. The thresholds of |NES| > 1 and adjust. *P*-value < 0.05 were considered significant enrichment.

### Molecular docking

Molecular docking was carried out to predict the combined modalities and capacities between potential active components of QXTTF and key genes. The 2D protein structures of potential active components (SDF file) were extracted from the PubChem database (https://pubchem.ncbi.nlm.nih.gov/), and 2D structures were then converted to 3D structures (PDB file) by Babel GUI software. These PDB files were integrated into AutoDockTools and exported as PDBQT format files as docking ligands. Subsequently, the 3D protein structures (PDB file) of key genes were downloaded from the UniProt database (https://www.uniprot.org) and added with nonpolar hydrogen using AutoDockTools software to calculate the charge and saved as a PDBQT file as an acceptor. After that, molecular docking was carried out utilizing AutoDockTools, and the results were displayed by PyMOL.

### Proteomic profiling

Proteomic profiling was performed at the Beijing Genomics Institute (Shenzhen, China) employing liquid chromatography-tandem mass spectrometry (LC-MS/MS) technology. Cellular specimens were lysed through ultrasonication in RIPA buffer supplemented with protease inhibitors, followed by centrifugal clarification (12,000 ×g, 15 min, 4°C) to obtain soluble fractions. Total protein concentration was determined using a bicinchoninic acid (BCA) assay. Protein samples underwent reduction with 10 mM dithiothreitol (DTT), alkylation with 50 mM iodoacetamide (IAA), and enzymatic digestion with sequencing-grade trypsin (37 °C, 16 h). The resultant peptides were desalted using C18 solid-phase extraction columns, subsequently labeled with TMT 11-plex isobaric tags, pooled in equimolar ratios, and lyophilized under vacuum.

Chromatographic separation was achieved through reversed-phase nanoflow liquid chromatography on an EASY-nLC 1200 system, employing a 120-min linear gradient. Eluted peptides were analyzed using a Q Exactive HF-X hybrid quadrupole-Orbitrap mass spectrometer operated in data-dependent acquisition (DDA) mode, with MS1 resolution set to 70,000 (at m/z 200) and MS2 resolution to 17,500. Raw spectral data were processed through the MaxQuant computational platform (v2.1.0) against the UniProtKB/Swiss-Prot human proteome database. Differentially expressed proteins were identified using stringent thresholds (|log2 fold change| ≥ 1, adjusted p-value < 0.05). Quality assurance measures included triplicate technical replicates and bovine serum albumin (BSA) spike-in controls demonstrating detection sensitivity ≤ 10 fmol.

### Statistical analysis

The R software and GraphPad Prism were used for statistical analysis. The differences were compared through the T-test. Statistical correlations were quantified through application of the Pearson correlation coefficient method. The *P*-value less than 0.05 was considered to be statistically significant.

## Results

### Basic characteristics of participants

This study enrolled 15 BC individuals and 63 QDC individuals (including 26 QDC-control and 37 QDC-QXTTF) from July 2023 to May 2024. As shown in **Table 2**, in both the QDC-control and QDC-QXTTF, the number of female individuals was higher than that of the male individuals. Moreover, the number of individuals with comorbidity, smoking, and drinking in the QDC group was higher than in the BC group. All baseline characteristics exhibited no statistically significant differences (*P* > 0.05).

**Table 2:** Basic characteristics of participants.

Characteristics	BC	QDC		*P*-value
		QDC-control	QDC-QXTTF	
Age (Mean ± SD)	35.87 ± 5.16	37.46 ± 5.36	36.70 ± 5 .73	0.63
Gender				
Male	6 (40.00%)	8 (30.77%)	11 (29.73%)	0.77
Female	9 (60.00%)	18 (69.23%)	26(70.27%)
Comorbidity				
Hypertension	0	1 (3.85%)	2 (5.41%)	0.67
Fatty liver	0	3 (11.54%)	1 (2.70%)	0.18
Diabetes	0	0	0	1
Other	0	2 (7.69%)	1 (2.70%)	0.43
History of Alcohol and Tobacco				
Smoking	1 (6.67%)	3 (11.54%)	4 (10.81%)	0.88
Drink	1 (6.67%)	2 (7.69%)	4 (10.81%)	0.86

### QDC subjects exhibit molecular signatures closely associated with immune function

**Figure 1:**
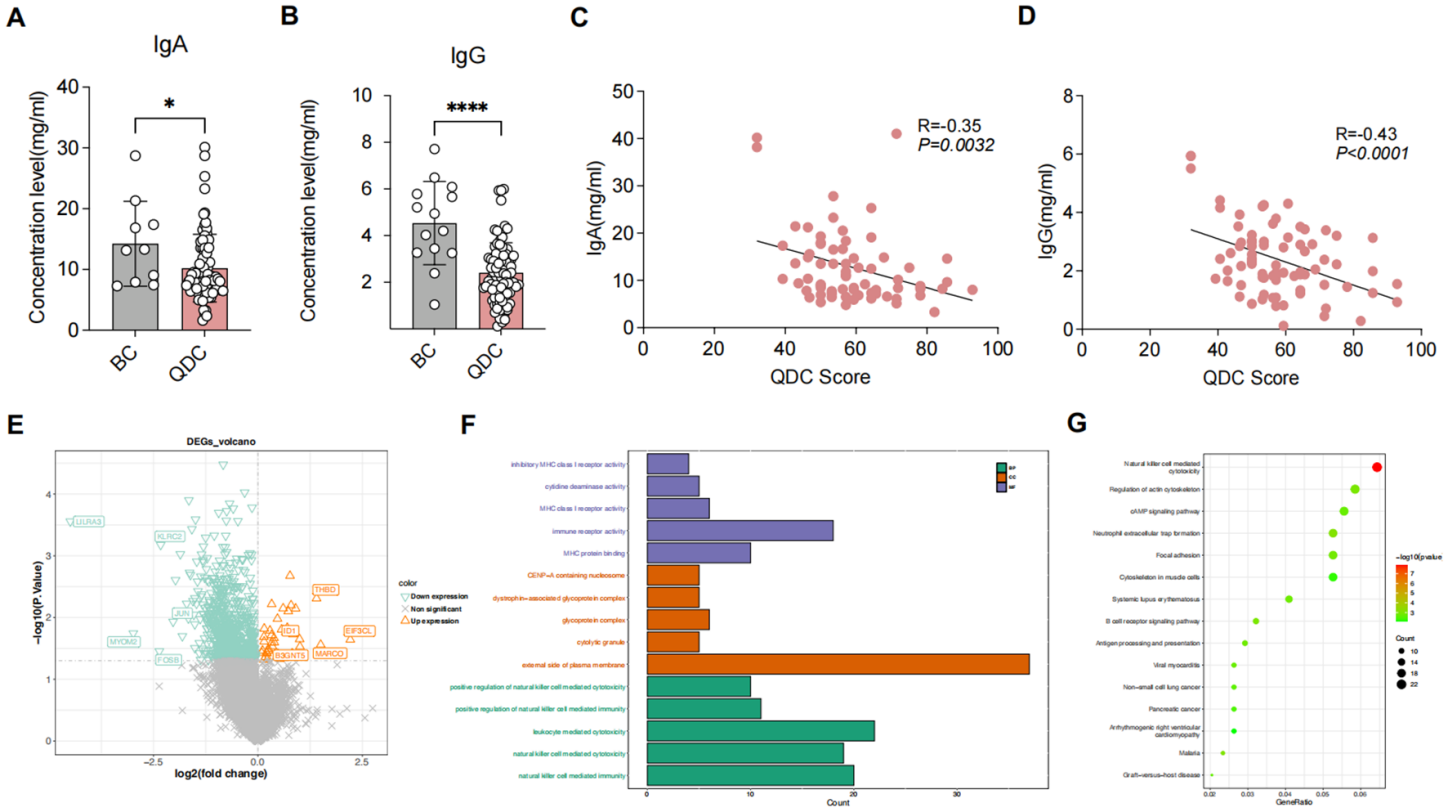
Differential analysis between QDC and BC groups **(A, B)** Comparison of immune-related indicators between QDC and BC groups **(A)** IgA (Unpaired t-test; n= 63/15) **(B)** IgG (Unpaired t-test; n= 63/15) **(C, D)** Correlations between Chinese medicine constitution score and immune-related indicators **(C)** Correlations between Chinese medicine constitution score and IgA (Linear-regression analysis; n= 63) **(D)** Correlations between Chinese medicine constitution score and IgG (Linear-regression analysis; n= 63) **(E)** The volcano plot showing the DEGs between pre-intervention QDC-control and BC groups (Linear models and empirical Bayesian methods; n = 6/3) **(F, G)** Enrichment analysis for DEGs1 **(F)** Gene Ontology **(G)** Kyoto Encyclopedia of Genes and Genomes (KEGG). * *P* < 0.05, **** *P* < 0.0001. QDC, qi-deficiency constitution; BC, balanced constitution; IgA: immunoglobulin A; IgG: immunoglobulin G; DEGs, the differentially expressed genes; KEGG, Kyoto Encyclopedia of Genes and Genomes.

In terms of immune-related indicators, the IgA and IgG levels in the QDC group were significantly declined than those in the BC group (**Figures 1A, B**). Moreover, correlation analysis demonstrated that IgA and IgG had both significantly negative relevance to the QDC score (IgA: R = -0.35, *P* = 0.0032 < 0.05; IgG: R = -0.43, *P* < 0.001) (**Figures 1C, D**).

Given these changes, we further evaluated the variations between QDC and BC groups at the transcription level. QC of raw reads was conducted using SOAPnuke (version 2.3), QC Metrics: Average sequencing depth = 6.2 Gb/sample; mapping rate to GRCh38.p13 reference genome = 92.3 ± 2.1%; Q30 score = 94.7 ± 1.5%. Sequencing data were suitable for subsequent analysis.

Differential analysis showed that a total of 846 DEGs1 were identified, comprising 40 up-regulated and 806 down-regulated genes (**Figure 1E**). Functional enrichment revealed that a total of 584 GO items (consisting of 451 GO BP items, 69 GO CC items, and 64 GO MF items) and 51 KEGG pathways were significantly enriched (**Supplementary Table S1**). As shown in **Figures 1F, G**, the top 5 GO items of each part and the top 15 KEGG pathways were visualized, such as “natural killer cell-mediated immunity (GO-BP),” “external side of plasma membrane (GO-CC),” “immune receptor activity (GO-MF),” and “natural killer cell-mediated cytotoxicity (KEGG).” These results indicated that different immunoregulatory mechanisms possibly might exist between BC individuals and QDC individuals, implying that QDC has a predisposition to immune-related diseases.

### QXTTF effectively reduced the QDC score and enhanced plasma immunoglobulin levels

**Figure 2:**
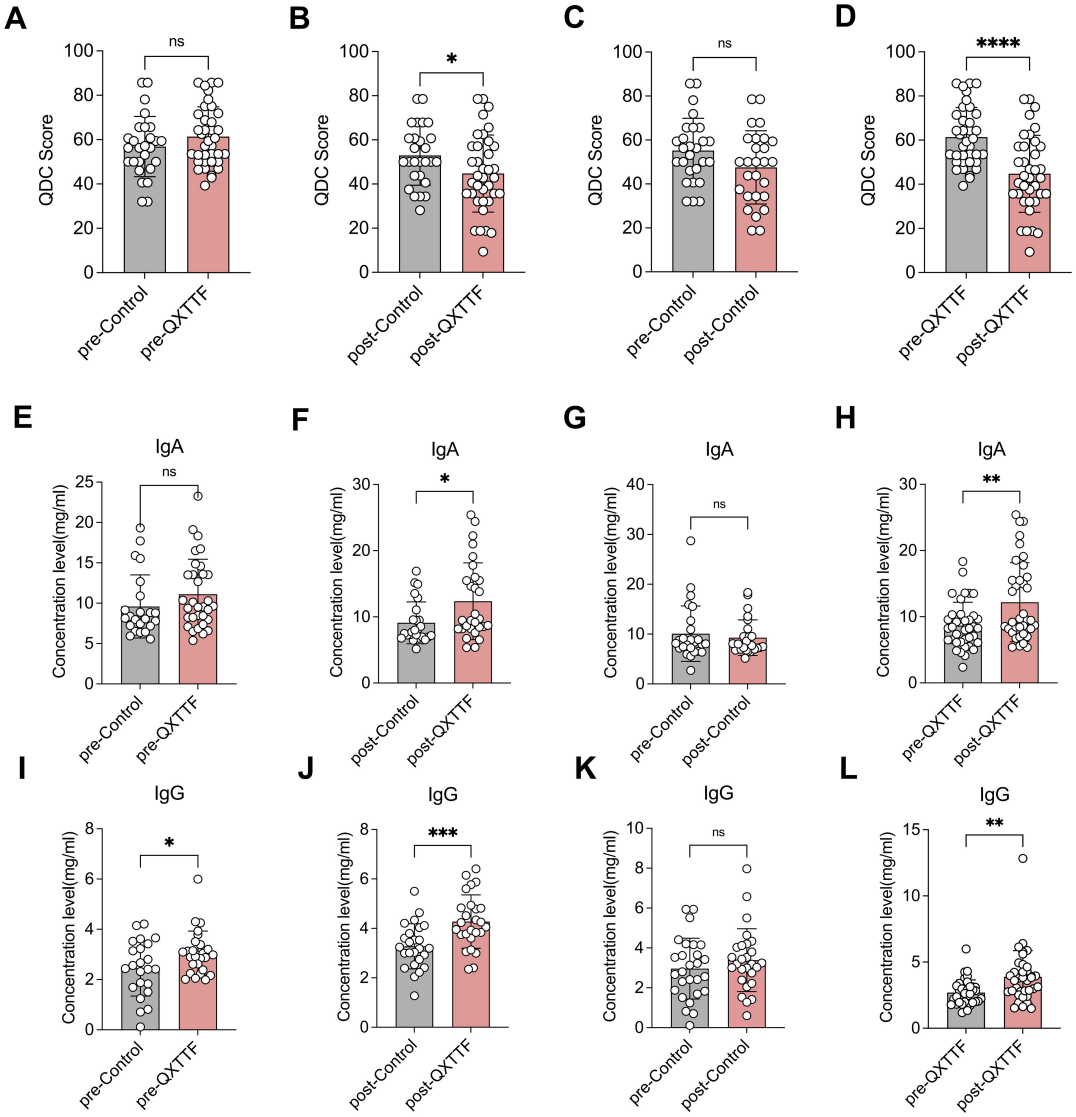
Outcomes of QXTTF treatment for QDC **(A-D)** Primary outcomes **(A)** Box plot showing the difference in Chinese medicine constitution QDC scores between pre-intervention QDC-QXTTF and pre-intervention QDC-control groups (Unpaired t-test, n= 37/26) **(B)** Box plot showing the difference in Chinese medicine constitution QDC scores between post-intervention QDC-QXTTF and post-intervention QDC-control groups (Unpaired t-test, n= 37/26) **(C)** Box plot showing the difference in Chinese medicine constitution QDC scores between post-intervention QDC-control and pre-intervention QDC-control groups (Paired t-test, n= 26) **(D)** Box plot showing the difference in Chinese medicine constitution QDC scores between post-intervention QDC-QXTTF and pre-intervention QDC-QXTTF groups (Paired t-test, n= 37) **(E-L)** Secondary outcomes **(E)** Box plot showing the difference in IgA between pre-intervention QDC-QXTTF and pre-intervention QDC-control groups (Unpaired t-test, n= 37/26) **(F)** Box plot showing the difference in IgA between post-intervention QDC-QXTTF and post-intervention QDC-control groups (Unpaired t-test, n= 37/26) **(G)** Box plot showing the difference in IgA between post-intervention QDC-control and pre-intervention QDC-control groups (Paired t-test, n= 26) **(H)** Box plot showing the difference in IgA between post-intervention QDC-QXTTF and pre-intervention QDC-QXTTF groups (Paired t-test, n= 37) **(I)** Box plot showing the difference in IgG between pre-intervention QDC-QXTTF and pre-intervention QDC-control groups (Unpaired t-test, n = 37/26) **(J)** Box plot showing the difference in IgG between post-intervention QDC-QXTTF and post-intervention QDC-control groups (Unpaired t-test, n = 37/26) **(K)** Box plot showing the difference in IgG between post-intervention QDC-control and pre-intervention QDC-control groups (Paired t-test, n = 26) **(L)** Box plot showing the difference in IgG between post-intervention QDC-QXTTF and pre-intervention QDC-QXTTF groups (Paired t-test, n = 37). ns: no significance, *P < 0.05, **P < 0.01, *** *P* < 0.001, ****P < 0.0001. QXTTF, Qi-Xu-Tiao-Ti Formula; QDC, qi-deficiency constitution.

The QDC scores showed no significant difference between the QDC-QXTTF and QDC-control groups at baseline (**Figure 2A**). After the QXTTF intervention, the QDC scores in the post-intervention QDC-QXTTF group were considerably lower than that in the post-intervention QDC-control group (**Figure 2B**). Moreover, after intervention, the QDC scores between post-intervention QDC-control group and pre-intervention QDC-control group were not significantly different (**Figure 2C**). However, the QDC scores in the post-intervention QDC-QXTTF group were significantly lower compared to the pre-intervention QDC-QXTTF group (**Figure 2D**). In terms of IgA, there was no marked variation between pre-intervention QDC-QXTTF and QDC-control groups (**Figure 2E**). However, after intervention, the QDC-QXTTF group exhibited considerably higher IgA than that in the QDC-control group (**Figure 2F**). For IgG, both pre- and post-intervention, significant variations were observed between QDC-QXTTF and QDC-control groups, with the variation being more significant after the intervention (**Figure 2I, J**). Moreover, these two indicators were not significantly variable between post-intervention QDC-control and pre-intervention QDC-control (**Figure 2G, K**). Nevertheless, these two indicators were both markedly higher in the post-intervention QDC-QXTTF group than in the pre-intervention QDC-QXTTF group (**Figure 2H, L**). Additionally, none of the participants had adverse effects. Consequently, QXTTF had potential therapeutic effects on QDC.

### Identification of therapeutic targets for QXTTF in treating QDC

**Figure 3:**
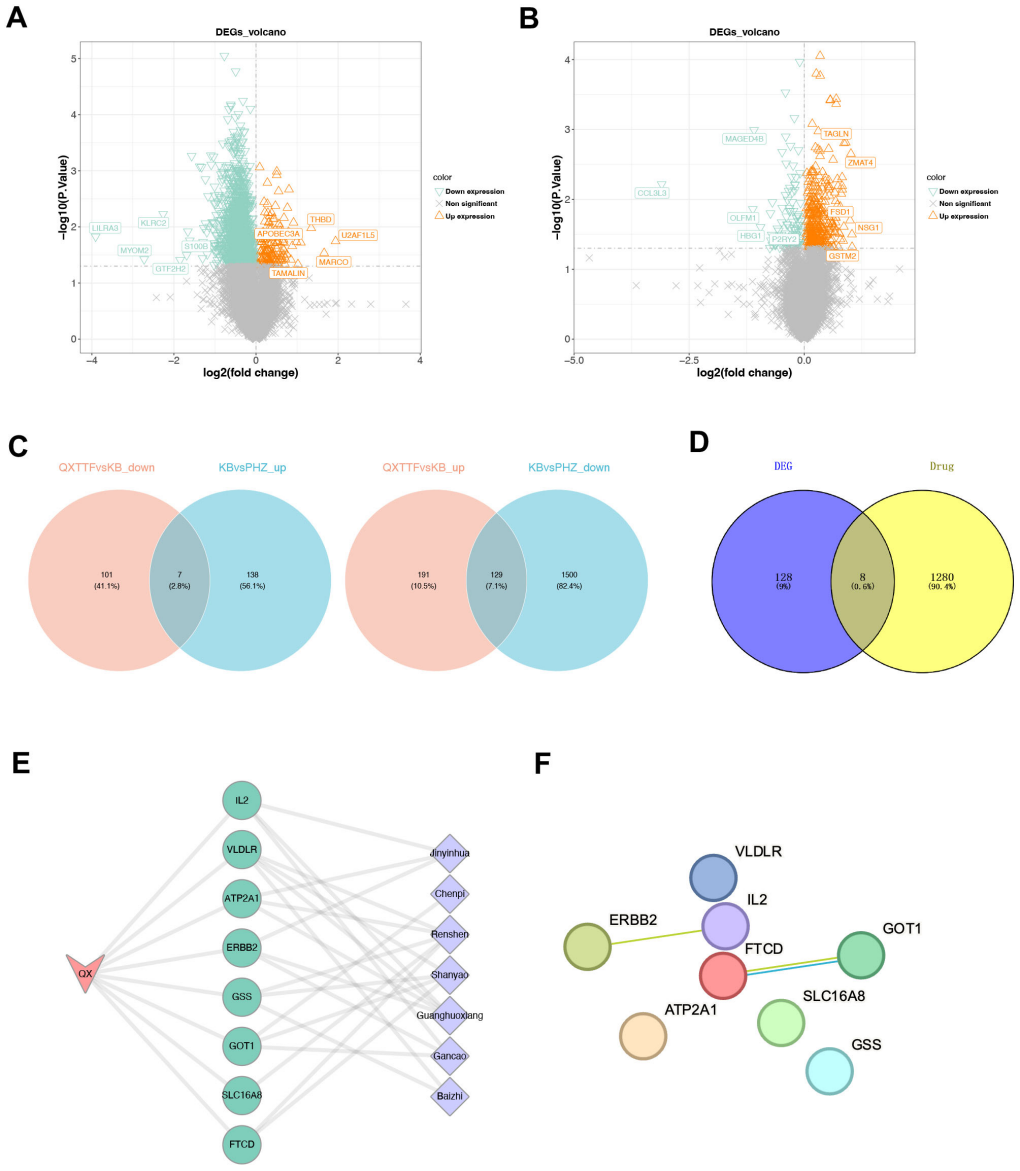
Identification of therapeutic targets for QXTTF in treating QDC **(A)** The DEGs between between post-intervention QDC-control and BC groups (Linear models and empirical Bayesian methods; n = 6/3) **(B)** The DEGs between post-intervention QDC-QXTTF and post-intervention QDC-control groups (Linear models and empirical Bayesian methods; n = 6/6) **(C)** The shared genes between DEGs2 and DEGs3 From left to right: the intersection between down-regulated genes in DEGs2 and up-regulated genes in DEGs3; the intersection between up-regulated genes in DEGs2 and down-regulated genes in DEGs3 **(D)** The intersection between drug-target genes and disease-target genes **(E)** The drug-key genes-disease network **(F)** The protein-protein interaction (PPI) network. QXTTF, Qi-Xu-Tiao-Ti Formula; QDC, qi-deficiency constitution; DEGs, the differentially expressed genes; PPI, protein-protein interaction.

Given the results we obtained above, we further identified therapeutic targets for QXTTF in treating QDC through network pharmacology. Through the TCMSP database, a total of 156 potential active components and 266 drug-target genes were obtained. Through the ETCM database, 1,130 drug-target genes were retrieved. After integration, a total of 1,288 drug-target genes were enrolled in our work. Subsequently, through differentially expressed analyses, 1,774 DEGs2 (145 up-regulated genes and 1,629 down-regulated genes) and 428 DEGs3 (320 up-regulated and 108 down-regulated genes) were discovered (**Figures 3A, B**). Following intersecting, there were 136 QDC-target genes (**Figure 3C**). Subsequently, a total of eight potential therapeutic targets (*ATP2A1*, *GOT1*, *SLC16A8*, *FTCD*, *ERBB2*, *GSS*, *VLDLR*, *IL2*) for QXTTF in treating QDC were identified (**Figure 3D**). As shown in **Figure 3E**, the drug-key genes-disease network was visualized, where pink, green, and purple represented QDC, key genes, and Chinese medicine ingredients of QXTTF, respectively. Furthermore, the PPI network contained eight nodes and two edges (**Figure 3F**). The robust interactions were observed between *ERBB2* and *IL2*, as well as between *FTCD* and *GOT1*.

### Core target genes of QXTTF are enriched in multiple immune-related gene pathways

**Figure 4:**
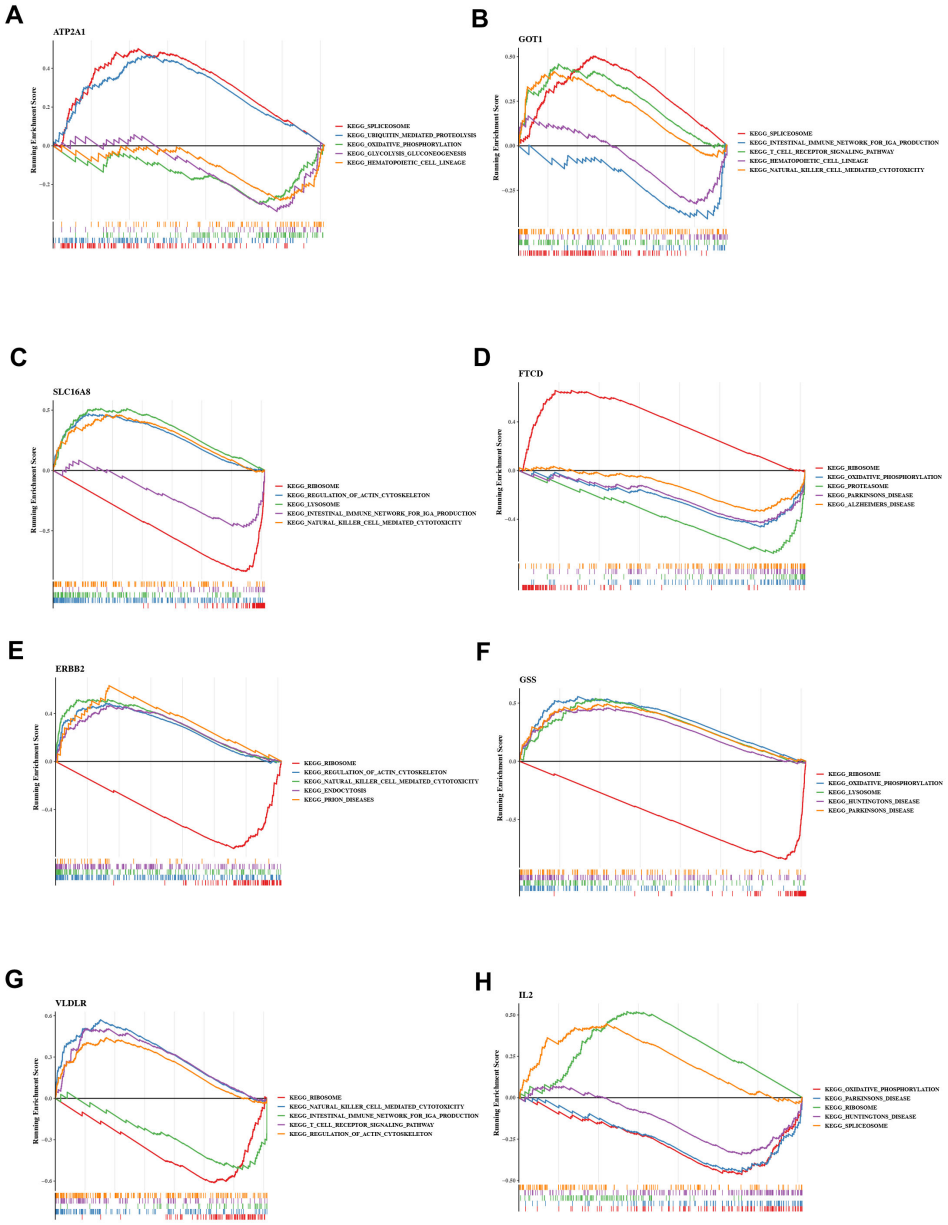
Gene set enrichment analysis (GSEA) **(A)** *ATP2A1* **(B)** *GOT1* **(C)** *SLC16A8* **(D)** *FTCD* **(E)** *ERBB2* **(F)** *GSS* **(G)** *VLDLR* **(H)** *IL2* GSEA: Gene set enrichment analysis.

To investigate the possible mechanisms by which QXTTF targeted key genes for the treatment of QDC, we conducted a single-gene GSEA-KEGG pathway analysis. **Figures 4A-H** visualize the top 5 KEGG pathways for each key gene enrichment. After a comprehensive analysis, we found the complex role of these key genes in disease-related pathways. Specifically, in terms of immune, excepting *FTCD*, remained seven key genes were significantly relevant to “natural killer cell-mediated cytotoxicity”. *GOT1*, *SLC16A8*, *ERBB2*, and *VLDLR* were markedly associated with “T cell receptor signaling pathway” and “intestinal immune network for IgA production.” Moreover, *GSS* was also related to “intestinal immune network for IgA production.” *ERBB2* was involved into “B cell receptor signaling pathway” and “primary immunodeficiency.” Some genetic information processing (translation)-related pathways (including “Ribosome” (*SLC16A8*, *FTCD*, *ERBB2*, *GSS*, *VLDLR*, and *IL2*), “Spliceosome” (*ATP2A1*, *GOT1*, and *IL2*), metabolism-related pathways (such as “oxidative phosphorylation” (*ATP2A1*, *SLC16A8*, *FTCD*, *GSS*, and *IL2*), “glycolysis gluconeogenesis” (*ATP2A1*, *FTCD*, *GSS*, and *IL2*), neurodegenerative disease-related pathways (including “Parkinson disease” (*SLC16A8*, *FTCD*, *GSS*, and *IL2*), “Alzheimer’s disease” (*SLC16A8*, *FTCD*, *GSS*, and *IL2*), “Huntington’s disease”(*SLC16A8*, *GSS*, and *IL2*), and other pathways were significantly enriched.

### Molecular docking between key genes and potential active components

**Figure 5:**
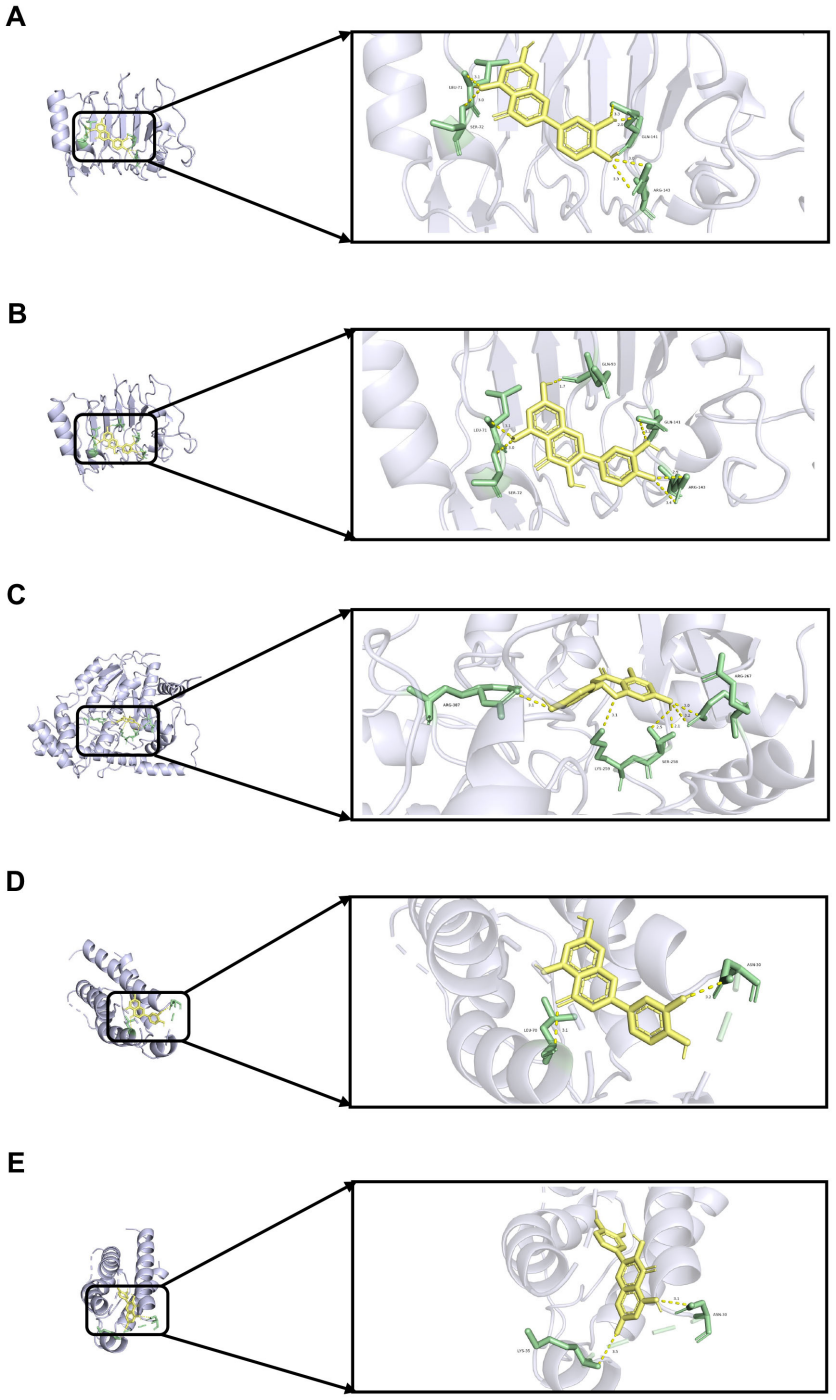
Molecular docking **(A)** Molecular docking between *ERBB2* and luteolin **(B)** Molecular docking between *ERBB2* and quercetin **(C)** Molecular docking between *GOT1* and naringenin **(D)** Molecular docking between *IL2* and luteolin **(E)** Molecular docking between *IL2* and quercetin.

To validate the findings of the network pharmacology, molecular docking was conducted to evaluate the binding affinity between key genes and potential active components. In general, as the binding energy between ligand and receptor decreases, the stability of the binding conformation increases. **Table 3** displayed the binding energy between key genes and potential active components, and the binding energy was all less than -5 kcal/mol, suggesting that these four potential active components bind well to *ERBB2*, *GOT1*, and *IL2*, and they may be crucial in the treatment of QDC. The molecular docking results are shown in **Figure 5**.

**Table 3:** Binding energy between key genes and potential active components.

herb_pinyin	molecule_name	PubChem CID	Symbol	Identifier	Energy (kcal/mol)
Jinyinhua	luteolin	5280445	*ERBB2*	8 JYR	-6.7
Huoxiang	quercetin	5280343	*ERBB2*	8 JYR	-6.6
Gancao	quercetin	5280343	*ERBB2*	8 JYR	-6.6
Jinyinhua	quercetin	5280343	*ERBB2*	8 JYR	-6.6
Chenpi	naringenin	439246	*GOT1*	6 DNB	-8.0
Gancao	naringenin	439246	*GOT1*	6 DNB	-8.0
Jinyinhua	luteolin	5280445	*IL2*	8 SOZ	-6.1
Jinyinhua	quercetin	5280343	*IL2*	8 SOZ	-6.0
Gancao	quercetin	5280343	*IL2*	8 SOZ	-6.0
Huoxiang	quercetin	5280343	*IL2*	8 SOZ	-6.0

### Core target genes of QXTTF modulating protein expression implicated in diverse immune signaling pathways

**Figure 6:**
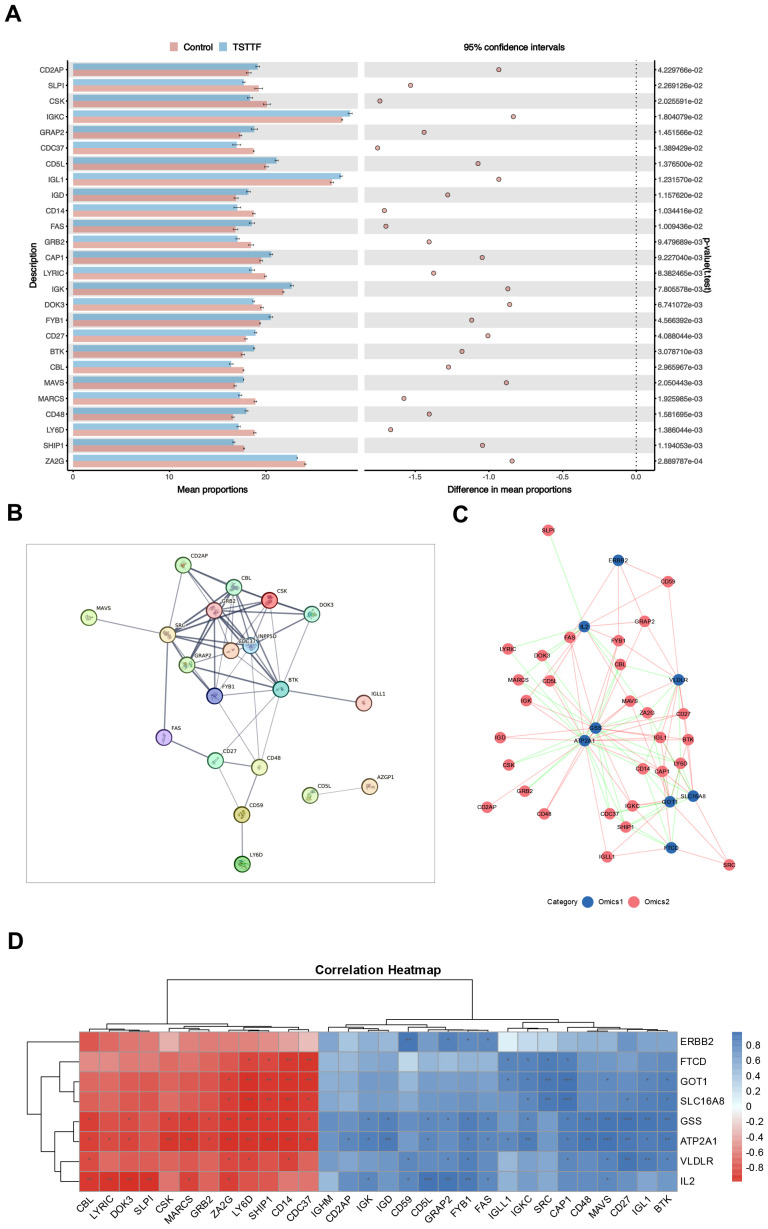
Target validation based on proteomics. **(A)** Differential expression profiles of core immune pathway-associated proteins across experimental groups (Unpaired t-test, n = 6/6) **(B)** The protein-protein interaction (PPI) network **(C)** Correlation network diagram of potential target genes and core immune pathway-associated proteins (Pearson correlation coefficient method, n = 6/6) **(D)** Correlation heatmap of potential target genes and core immune pathway-associated proteins. PPI, protein-protein interaction.

Furthermore, we conducted proteomic-level validation by performing a comprehensive systematic screening of proteins linked to key enriched signaling pathways. The investigation focused on three critical immune pathways: “natural killer cell-mediated cytotoxicity”, “T cell receptor signaling pathway”, and “B cell receptor signaling pathway”, wherein we identified 30 pathway-associated proteins. Comparative analysis revealed that 26 proteins exhibited significant differential expression in the post-intervention QDC-QXTTF group versus the post-intervention QDC-Control group (**Figure 6A**), comprising 14 upregulated and 12 downregulated proteins. The remaining four proteins (CD59, IGHM, IGLL1, and SRC) demonstrated non-significant elevation trends, as detailed in **Supplementary Table S2**. BTK emerged as the central regulatory protein in B cell signaling (15); FYB1, CD2AP, and SRC principally mediate T cell signaling (16, 17); CBL, DOK3, GRB2, LY6D, and CSK function as pivotal regulators of immune signaling through the coordinated inhibition of T-cell, B-cell, and NK-cell activating receptors (18, 19). The protein-protein interaction (PPI) network demonstrated extensive interconnectivity among 20 nodal components, with key regulators including SRC, GRB2, CBL, CSK, BTK, DOK3, FYB1, and CD2AP establishing significant interaction affinities (**Figure 6B**). These findings substantiate QXTTF’s capacity to modulate immune functionality through differential expression of pathway-associated proteins.

Subsequent association network analysis (**Figure 6C**) revealed distinct topological relationships: blue nodes denoting potential target genes and pink nodes representing pathway-associated proteins, with pink/green edges indicating positive/negative correlations respectively. The correlation heatmap (**Figure 6D**) further demonstrated that all eight key target genes demonstrated significant multivariate correlations with multiple proteins. This multilevel integration confirms that these key target genes exert regulatory control over critical immune pathway proteins, thereby validating their function at the proteomic level.

## Discussion

The alterations of modern lifestyle, including delayed sleep, reduced exercise, long-term mental stimulation, postoperative, and postpartum states, trauma, radiotherapy, and drug abuse, have significantly transformed the disease spectrum, and the patient’s conditions are complex and diverse, yet all of them exhibit symptoms of qi-deficiency, such as fatigue, shortness of breath with reluctance to speak, pale tongue, susceptible to infectious diseases and so on. And our study suggests that QDC participants exhibited a pronounced downregulation of 806 genes (*vs*. 40 upregulated) compared to BC, consistent with an immunosuppression state characterized by impaired NK cell cytotoxicity and T/B cell signaling (**Figures 1E–G**). This transcriptional signature aligns with clinical observations of increased susceptibility to infections in QDC individuals. Therefore, there is an urgent need to increase attention to this population. TCM has more advantages in managing sub-health conditions. This study investigated the regulatory impact of the QXTTF on the QDC via a single-center randomized controlled trial. We identified potential targets of the QXTTF in modulating the QDC through transcriptomics and network pharmacology, aiming to offer a more effective therapeutic approach for the clinical diagnosis and treatment of the QDC, enhance public health, and promote the application of TCM in the management of sub-health and prevention of infectious diseases.

Previous research has demonstrated that QDC can result in diminished immune markers in patients or test subjects, exhibiting a notable reduction in immune function relative to normal levels (14, 20). Furthermore, immune-related genes, such as those associated with the major histocompatibility complex and interleukin-1β, are down-regulated in individuals with the QDC (21, 22). Immunoglobulins (IgA, IgM, IgG) constitute the foundation of humoral immunity and are crucial in disease prevention and resistance. In our study, IgA and IgG in individuals with the QDC were significantly lower than in those with the BC, suggesting that immune function was reduced in individuals with the QDC. The supplementation of qi can significantly improve various immune indicators in patients, serving as an effective method to enhance immune function. It is applicable to individuals of QDC, YADC, BSC, and patients with low T-cell subpopulations, immunoglobulins, and complement levels. A study on the treatment of qi deficiency syndrome with modified Renshen Yangrong decoction demonstrated that levels of immunoglobulins (IgA, IgM, IgG) and complement components (C3, C4) were significantly enhanced in treated people compared to pre-treatment levels (*p* < 0.05) (22). In our study, in terms of the Chinese medicine constitution score, individuals with the QDC who received QXTTF intervention exhibited decreased scores compared to pre-intervention, alongside significantly elevated levels of IgA and IgG, indicating that QXTTF is effective in adjusting QDC and may function through immune regulation.

To investigate the potential mechanism of action of QXTTF in regulating the QDC, we conducted a network pharmacology analysis. Results demonstrated that *ATP2A1*, *GOT1*, *SLC16A8*, *FTCD*, *ERBB2*, *GSS*, *VLDLR*, and *IL2* were potential targets for QXTTF to adjust the QDC. *ATP2A1*, a crucial enzyme for the maintenance of intracellular calcium homeostasis, is also a cancer-related immune marker. In cholangiocarcinoma, *ATP2A1* is highly expressed and associated with the hyperimmune state of the tumor microenvironment (23). In colorectal cancer, *ATP2A1* expression was markedly and negatively linked with the infiltration abundance of CD8 + T cells, suggesting that *ATP2A1*-promoted tumor growth may be relevant to the immune (24). *GOT1*, a cytoplasmic glutamate oxaloacetate transaminase, is essential to various metabolic pathways crucial for cellular homeostasis and metabolic dysregulation (25). Previous studies have indicated that *GOT1* has emerged as a pivotal factor in the investigation of cancer metabolism. Specifically, *GOT1* is involved in several critical physiological processes, including aspartate synthesis, glutamine metabolism regulation, glycolytic pathway modulation, regulation of immune cell function, and epigenetic regulatory mechanisms, all of which significantly facilitate metabolic reprogramming in cancer (25). *SLC16A8*, encoding MCT3, is a member of the SLC16A family, which is correlated with the transport of monocarboxylates like lactate and pyruvate, and is highly involved in metabolic processes and the maintenance of acid-base balance (26). *FTCD* not only has the function of the enzyme but also plays a structural role in connecting biofilms, exhibiting expression in nearly all mammals, especially in the liver (27). Moreover, its expression level is a powerful diagnostic predictor for distinguishing hepatocellular carcinoma from benign tumors (28). *ERBB2*, a tyrosine kinase, binds to the ligands of its homologous family members to form a dimer, which activates downstream signaling pathways (including the RAS-MARK and PIK3-AKT pathways), thereby modulating cellular proliferation, differentiation, and the inhibition of apoptosis, among other functions (29). *GSS* is crucial for the synthesis of glutathione, which is an essential intracellular antioxidant and significantly contributes to the process of ferroptosis (30). *VLDLR* is a cell surface receptor with various functions, including binding to and facilitating the endocytosis of very low-density lipoproteins, thereby promoting energy metabolism (31). *IL2*, predominantly released by antigen-activated CD4 + T cells, is pivotal in cancer immunotherapy. Functionally, *IL2* promotes T cell proliferation and differentiation, enhances NK cell activity, and stimulates the generation of cytotoxic T lymphocytes (CTLs) (32). The results of GSEA demonstrated that these targets were considerably relevant to immune, metabolic, genetic information processing (translation), and other related pathways, revealing that these genes were involved in complex biological processes. Consequently, through targeting the potential targets, QXTTF may regulate multiple pathways (such as immune regulation, metabolic reprogramming, cell growth, apoptosis, etc.) to regulate the QDC. These results provide a new perspective for the study of the mechanism of QXTTF and provide a theoretical basis for its application in the intervention and treatment of QDC.

Then, molecular docking was further conducted, and results illustrated that luteolin, quercetin, and naringenin may be crucial potential active components for QXTTF in regulating the QDC. These three compounds belong to flavonoid compounds and have antioxidant, anti-inflammatory, anti-cancer, cardiovascular protection, and other effects. Previous research has demonstrated that luteolin effectively targets inflammation-related signaling pathways (such as NF-κB, MAPKs, JAK/STAT, etc.), thus diminishing the production of pro-inflammatory cytokines, the migration of inflammatory cells, and the expression of inflammatory genes (33). Quercetin exerts its potent anti-inflammatory effects mainly through inhibition of cytokine production, reduction of cyclooxygenase and lipoxygenase expression, and maintenance of mast cell stability, as well as its antioxidant activity by affecting the activity of glutathione, enzymes, and reactive oxygen species (ROS), and regulating signal transduction pathways, such as heme oxygenase 1/nuclear factor erythroid 2-related factor (Nrf2), mitogen-activated protein kinase, toll-like receptor 4/phosphatidylinositol 3-kinase, and 5’-monophosphate-activated protein kinase (34). Naringenin demonstrates anti-inflammatory effects by inhibiting the NF-κB signaling pathway, activating Nrf2, which promotes heme oxygenase-1 release from macrophages, and reducing the expression of Th1 cytokines and inflammatory mediators (35). It also blocks xanthine oxidase, antagonizes ROS, scavenges superoxide radicals, reduces oxygen-induced accessibility of K+ red blood cells, and reduces lipid peroxidation (35). These results further indicate that the potential mechanism of QXTTF as a traditional Chinese medicine compound is to regulate QDC through the comprehensive effect of various compounds.

Furthermore, proteomic validation was conducted through systematic screening of proteins associated with key enriched pathways, which demonstrated significant differential expression. Mechanistically, CBL, DOK3, GRB2, LY6D, and CSK exert immunomodulatory effects through two distinct mechanisms: 1) attenuating immune cell activation via inhibitory regulation of T cell, B cell, and NK cell activating receptors; 2) suppressing inflammatory mediator release and impairing immune cell functionality through NF-κB pathway blockade (36–39). Conversely, FYB1, CD2AP, and SRC primarily mediate T cell signaling by facilitating cellular proliferation/differentiation while paradoxically activating the NF-κB pathway to potentiate immune responses and augment NK cell cytotoxic activity (40, 41). BTK was identified as the central regulatory node in B cell signaling, coordinating downstream NF-κB pathway activation to maintain B cell homeostasis (42).

These proteins exhibit intricate interaction networks while showing significant correlations with expression patterns of previously identified QXTTF-associated candidate genes. This molecular interplay suggests their putative regulatory function in immune processes through modulation of immune pathway-related protein expression. Collectively, these findings establish proteomic-level validation of the intrinsic mechanism through which QXTTF mediates regulation of QDC homeostasis.

**Figure 7:**
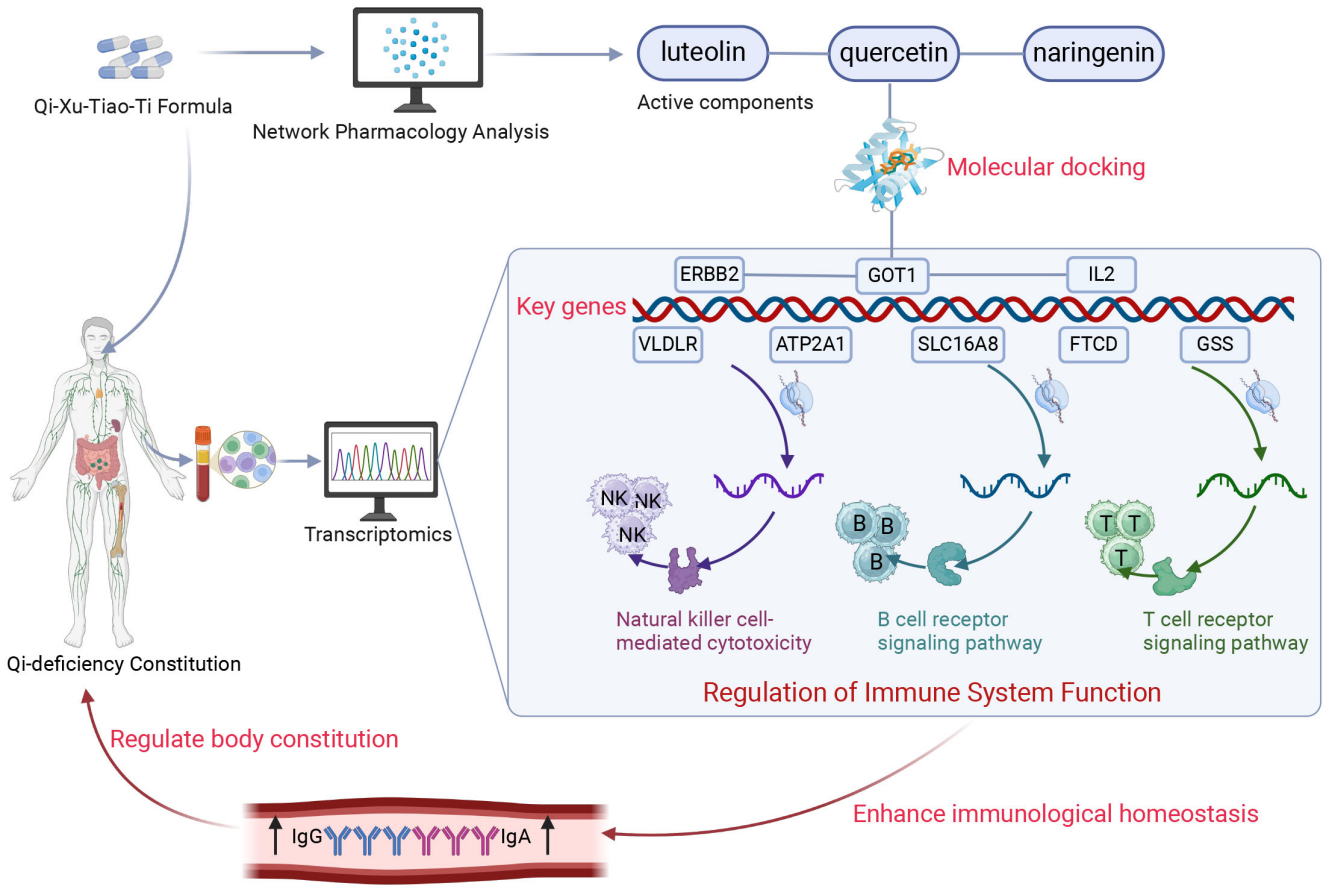
Schematic diagram of the present research. Through integrated transcriptomic profiling and pharmacological network analysis, the current investigation revealed that QXTTF manifests its multicomponent therapeutic activity via coordinated regulation of eight putative target genes (*ATP2A1*, *GOT1*, *SLC16A8*, *FTCD*, *ERBB2*, *GSS*, *VLDLR*, and *IL2*). These molecular targets exhibit statistically significant enrichment in immune-related biological processes, particularly natural killer cell-mediated cytotoxicity, T-cell receptor signaling cascade, and B-cell receptor activation pathways. This integrated analysis substantiates that QXTTF mediates therapeutic effects on QDC through potential immunomodulatory mechanisms involving both innate and adaptive immune system regulation. QXTTF, Qi-Xu-Tiao-Ti Formula; QDC, qi-deficiency constitution.

Our research indicates that QXTTF may help to restore the balance of QDC, enhance immune function, and promote cellular metabolism and antioxidant activity by targeting and modulating immune cells, metabolic pathways, and antioxidant and anti-inflammatory signaling pathways, thereby improving overall health and alleviating various pathological conditions associated with qi-deficiency (**Figure 7**). Despite our discussion on the modulatory effects of QXTTF on QDC and the potential targets, the limitations of our study persist. Initially, the scale is influenced by the participants’ subjective consciousness to some degree, resulting in a lack of objectivity in the outcomes. Secondly, the study is conducted at only one medical center, and the results should also be validated in a larger clinical sample; and immune assessment in the present study was confined to IgA and IgG measurements, so subsequent investigations should incorporate analyses of T-cell subsets and cytokine profiles to holistically assess immune function. Thirdly, the compound-target network derived from public databases may contain spurious associations, which is an inherent limitation of network pharmacology. This study performs preliminary identification of potential targets and pathways for QXTTF; however, mechanistic validation remains limited to correlational analyses. Future investigations will focus on validating key targets through *in vitro* experiments and animal models. Therefore, while this study demonstrates the potential of QXTTF in regulating QDC, additional clinical data and experimental research are required to validate its efficacy and refine the treatment strategy to guarantee its safety and effectiveness in practical applications.

## Conclusion

Through a clinical trial, our research has revealed the effectiveness of QXTTF in regulating the QDC. Moreover, we investigate possible mechanisms through network pharmacology and identify eight potential targets (*ATP2A1*, *GOT1*, *SLC16A8*, *FTCD*, *ERBB2*, *GSS*, *VLDLR*, and *IL2*). Our research demonstrates that QXTTF exhibits significant potential for modulating QDC, which may enhance immune function and resistance to infections. In the forthcoming studies, we will undertake more *in vivo* and *in vitro* investigations to provide reliable evidence regarding the application of QXTTF.

## Data Availability

The data generated during the course of this investigation are not publicly accessible due to ethical restrictions pertaining to the privacy of human participants. Requests to access de-identified datasets may be directed to the corresponding author or the Ethics Committee of Shenzhen Hospital of Beijing University of Chinese Medicine, accompanied by a scientifically justified research proposal and evidence of relevant ethical approval (if applicable).
