# Supplementary material for: Immunomodulatory effects and mechanisms of Qi-Xu-Tiao-Ti formula in Qi-deficiency constitution: a randomized controlled trial integrated with multi-omics and network pharmacology analysis
Source: Front Immunol. 2025 Oct 31;16:1675502. doi: 10.3389/fimmu.2025.1675502 (PMC12615400; doi:10.3389/fimmu.2025.1675502)
Supplement: Supplementary file 1 [file DataSheet1.pdf]

Supplementary Materials

Table of Contents

1 BGI sequencing sample test report..1

2 Quality control information of traditional Chinese medicine components contained in QXTTF (Attached with quality inspection report 1- 9).......... 25

2.1 Report 1: Lonicerae japonicae Flos (Chinese: Jinyinhua)...........26

2.2 Report 2: Phragmitis Rhizoma (Chinese: Lugen)........................27

2.3 Report 3: Imperatae Rhizoma (Chinese: Baimaogen)................ 28

2.4 Report 4: Pogostemonis Herba (Chinese: Huoxiang)................. 29

2.5 Report 5: Angelicae dahuricae Radix (Chinese: Baizhi)............. 30

2.6 Report 6: Citri Reticulatae Pericarpium (Chinese: Chenpi)......... 31

2.7 Report 7: Ginseng Radix et Rhizoma (Chinese: Renshen).........32

2.8 Report 8: Dioscoreae Rhizoma (Chinese: Shanyao).................. 33

2.9 Report 9: Glycyrrhizae Radix et Rhizoma (Chinese: Gancao).... 34

BGI sequencing sample test report

Ⅰ Project Information

Report number: TWH23111858

Project Name	Tissue Extraction Contract			Project Number	F19KF0001		
Client Name	Ma Zhenghua			Client Unit	Tissue Extraction Special Project		
Lab Sample Receiver	Lei Hongping			Lab Sample Receipt Date	20231115		
Laboratory Tester	Sun Si			Lab Test Date	20231117		
Lab Name	High-throughput Laboratory of Wuhan BGI Genomics Technology Service Co., LTD			Lab Address	B2 Floor, No.666, Gaoxin Avenue, Donghu Development Zone, Wuhan City		
Report Writer	Sun Si	Report Reviewer	Cao Wei	Report Authorized Signatory	Peng Yun	Report Date	20231118

Ⅱ Sample Testing Methods

① Concentration detection methods: □ Agilent 2100, ■ Fragment Analyzer, □ ELISA reader, □ Agilent 4200

② 28S/18S or 23S/16S detection methods: □Agilent 2100, ■ Fragment Analyzer, □Qsep-400, □ Agilent 4200

③ RIN or RQN detection methods: □Agilent 2100, ■ Fragment Analyzer, □Qsep-400, □Agilent 4200

Ⅲ Sample Testing Results

Serial Number	Sample Name	Sample Number	Tissue Name	Sample Number	Remaining Tissue Quantity	Concentration (ng/μL)	volume (μL)	Total Amount (μg)	RIN/RQN	28S/18S	Library Type	Result Description	Remarks
1	TT230411-001A	WHYR231118147_A	TT230411-001	TWH2311021764	0	40	40	1.6	1.1	0.0	DNBSEQ Transcriptome	Class C	RQN<7.0,28S/18S<1.0
2	TT230411-002A	WHYR231118148_A	TT230411-002	TWH2311021765	0	54	40	2.16	1.3	0.0	DNBSEQ Transcriptome	Class C	RQN<7.0,28S/18S<1.0
3	TT230411-003A	WHYR231118149_A	TT230411-003	TWH2311021766	0	181	40	7.24	8.6	1.9	DNBSEQ Transcriptome	Class A	
4	TT230411-004A	WHYR231118150_A	TT230411-004	TWH2311021767	0	195	40	7.8	7.8	1.8	DNBSEQ Transcriptome	Class A	
5	TT230411-006A	WHYR231118151_A	TT230411-006	TWH2311021768	0	145	40	5.8	7.2	1.9	DNBSEQ Transcriptome	Class A	
6	LV230413-003A	WHYR231118152_A	LV230413-003	TWH2311021769	0	370	40	14.8	6.2	1.4	DNBSEQ Transcriptome	Class B	RQN<7.0
7	LV230413-004A	WHYR231118153_A	LV230413-004	TWH2311021770	0	192	40	7.68	6.8	1.6	DNBSEQ Transcriptome	Class B	RQN<7.0
8	LV230413-005A	WHYR231118154_A	LV230413-005	TWH2311021771	0	127	40	5.08	7.0	1.8	DNBSEQ Transcriptome	Class B	The baseline is slightly higher
9	LV230413-006A	WHYR231118155_A	LV230413-006	TWH2311021772	0	105	40	4.2	6.5	1.5	DNBSEQ Transcriptome	Class B	RQN<7.0
10	LV230414-006A	WHYR231118156_A	LV230414-006	TWH2311021773	0	182	40	7.28	6.5	2.1	DNBSEQ Transcriptome	Class B	RQN<7.0
11	LV230417-009A	WHYR231118157_A	LV230417-009	TWH2311021774	0	300	40	12	7.6	2.8	DNBSEQ Transcriptome	Class A	
12	LV230601-002A	WHYR231118158_A	LV230601-002	TWH2311021775	0	164	40	6.56	7.7	2.4	DNBSEQ Transcriptome	Class A	
13	LV230825-009A	WHYR231118159_A	LV230825-009	TWH2311021776	0	280	80	22.4	8.8	2.3	DNBSEQ Transcriptome	Class A	
14	LV230826-016A	WHYR231118160_A	LV230826-016	TWH2311021777	0	220	80	17.6	8.6	2.5	DNBSEQ Transcriptome	Class A	
15	LV230829-014A	WHYR231118161_A	LV230829-014	TWH2311021778	0	345	40	13.8	6.5	2.0	DNBSEQ Transcriptome	Class B	RQN<7.0
16	LV230829-015A	WHYR231118162_A	LV230829-015	TWH2311021779	0	410	40	16.4	6.2	2.1	DNBSEQ Transcriptome	Class B	RQN<7.0

17	LV230829-016A	WHYR231118163_A	LV230829-016	TWH2311021780	0	230	20	4.6	7.9	1.5	DNBSEQ Transcriptome	Class A	
18	LV230830-015A	WHYR231118164_A	LV230830-015	TWH2311021781	0	160	80	12.8	8.2	2.1	DNBSEQ Transcriptome	Class A	
19	LV230831-014A	WHYR231118165_A	LV230831-014	TWH2311021782	0	350	80	28	8.6	1.9	DNBSEQ Transcriptome	Class A	
20	LV230904-003A	WHYR231118166_A	LV230904-003	TWH2311021783	0	158	40	6.32	8.2	1.7	DNBSEQ Transcriptome	Class A	
21	LV230912-010A	WHYR231118167_A	LV230912-010	TWH2311021784	0	250	40	10	8.9	1.9	DNBSEQ Transcriptome	Class A	
22	LV230927-008A	WHYR231118168_A	LV230927-008	TWH2311021785	0	208	80	16.64	8.2	1.8	DNBSEQ Transcriptome	Class A	
23	LV231020-019A	WHYR231118169_A	LV231020-019	TWH2311021786	0	565	20	11.3	9.9	2.0	DNBSEQ Transcriptome	Class A	
24	LV231023-020A	WHYR231118170_A	LV231023-020	TWH2311021787	0	445	40	17.8	9.4	1.8	DNBSEQ Transcriptome	Class A	
25	TT230411-007A	WHYR231118171_A	TT230411-007	TWH2311021788	0	212	20	4.24	7.9	1.7	DNBSEQ Transcriptome	Class A	
26	TT230411-008A	WHYR231118172_A	TT230411-008	TWH2311021789	0	172	20	3.44	6.7	1.3	DNBSEQ Transcriptome	Class B	RQN<7.0
27	TT230411-009A	WHYR231118173_A	TT230411-009	TWH2311021790	0	195	40	7.8	8.3	1.6	DNBSEQ Transcriptome	Class A	
28	TT230411-010A	WHYR231118174_A	TT230411-010	TWH2311021791	0	198	20	3.96	7.0	2.0	DNBSEQ Transcriptome	Class B	The baseline is slightly higher
29	TT230411-011A	WHYR231118175_A	TT230411-011	TWH2311021792	0	470	20	9.4	6.9	1.2	DNBSEQ Transcriptome	Class B	RQN<7.0
30	TT230411-012A	WHYR231118176_A	TT230411-012	TWH2311021793	0	159	20	3.18	8.9	1.8	DNBSEQ Transcriptome	Class A	
31	LV230412-004A	WHYR231118177_A	LV230412-004	TWH2311021794	0	188	20	3.76	6.8	1.6	DNBSEQ Transcriptome	Class B	RQN<7.0
32	LV230421-008A	WHYR231118178_A	LV230421-008	TWH2311021795	0	325	20	6.5	9.1	1.3	DNBSEQ Transcriptome	Class A	
33	LV230513-012A	WHYR231118179_A	LV230513-012	TWH2311021796	0	198	20	3.96	7.3	1.4	DNBSEQ Transcriptome	Class A	
34	LV230513-013A	WHYR231118180_A	LV230513-013	TWH2311021797	0	190	20	3.8	9.6	2.2	DNBSEQ Transcriptome	Class A	
35	LV230513-014A	WHYR231118181_A	LV230513-014	TWH2311021798	0	171	20	3.42	9.1	2.0	DNBSEQ Transcriptome	Class A	
36	LV230602-004A	WHYR231118182_A	LV230602-004	TWH2311021799	0	385	40	15.4	9.1	1.5	DNBSEQ Transcriptome	Class A	
37	LV230904-004A	WHYR231118183_A	LV230904-004	TWH2311021800	0	320	20	6.4	9.9	1.7	DNBSEQ Transcriptome	Class A	
38	LV230906-001A	WHYR231118184_A	LV230906-001	TWH2311021801	0	173	80	13.84	7.7	1.7	DNBSEQ Transcriptome	Class A	
39	LV230906-002A	WHYR231118185_A	LV230906-002	TWH2311021802	0	229	80	18.32	8.2	1.9	DNBSEQ Transcriptome	Class A	
40	LV230907-008A	WHYR231118186_A	LV230907-008	TWH2311021803	0	149	80	11.92	8.9	2.0	DNBSEQ Transcriptome	Class A	
41	LV230907-009A	WHYR231118187_A	LV230907-009	TWH2311021804	0	183	80	14.64	8.8	2.1	DNBSEQ Transcriptome	Class A	
42	LV230907-010A	WHYR231118188_A	LV230907-010	TWH2311021805	0	100	80	8	9.3	2.1	DNBSEQ Transcriptome	Class A	
43	LV230907-011A	WHYR231118189_A	LV230907-011	TWH2311021806	0	161	80	12.88	9.0	2.2	DNBSEQ Transcriptome	Class A	
44	LV230908-011A	WHYR231118190_A	LV230908-011	TWH2311021807	0	227	80	18.16	7.7	1.8	DNBSEQ Transcriptome	Class A	
45	LV230908-012A	WHYR231118191_A	LV230908-012	TWH2311021808	0	191	40	7.64	8.4	2.0	DNBSEQ Transcriptome	Class A	
46	LV230912-009A	WHYR231118192_A	LV230912-009	TWH2311021809	0	290	80	23.2	8.7	1.8	DNBSEQ Transcriptome	Class A	
47	LV231018-006A	WHYR231118193_A	LV231018-006	TWH2311021810	0	210	40	8.4	10.0	2.0	DNBSEQ Transcriptome	Class A	
48	LV231026-013A	WHYR231118194_A	LV231026-013	TWH2311021811	0	161	80	12.88	9.5	2.1	DNBSEQ Transcriptome	Class A	
49	LV230905-016A	WHYR231118195_A	LV230905-016	TWH2311021812	0	235	80	18.8	8.9	1.9	DNBSEQ Transcriptome	Class A	

50	LV230922-002A	WHYR231118196_A	LV230922-002	TWH2311021813	0	320	40	12.8	10.0	2.0	DNBSEQ Transcriptome	Class A	
51	LV230904-005A	WHYR231118197_A	LV230904-005	TWH2311021814	0	113	80	9.04	10.0	2.2	DNBSEQ Transcriptome	Class A	
52	LV231023-018A	WHYR231118198_A	LV231023-018	TWH2311021815	0	220	80	17.6	10.0	2.1	DNBSEQ Transcriptome	Class A	
53	LV231024-013A	WHYR231118199_A	LV231024-013	TWH2311021816	0	295	80	23.6	10.0	2.0	DNBSEQ Transcriptome	Class A	
54	LV231023-019A	WHYR231118200_A	LV231023-019	TWH2311021817	0	179	80	14.32	9.3	2.0	DNBSEQ Transcriptome	Class A	

Note*: The test results are based on BGI Genomics sequencing sample testing standards and represent a comprehensive evaluation of the tested samples.

1. The results interpretation provides an opinion and explanation on whether the tested samples meet the requirements based on the RNA sequencing sample quality standard.

a) Class A refers to samples that meet the quality requirements for database sequencing. The current success rate is approximately 96.18%.

b) Class B refers to products that do not fully meet the quality standards for sample submission, but can still undergo risk-based sequencing. The current success rate is approximately 85.25%.

c) Class C refers to products that do not meet the standard quality requirements for sample submission, but can be evaluated and customized. The current success rate is approximately 70.48%.

2. According to BGIs historical data, Class B/C samples may face the following risks, among others:

a) Insufficient or excessively low total RNA: This may result in failed library construction, low library yield for sequencing, or insufficient sequencing data.

b) Degraded samples: may cause library construction failure; may result in high duplication rates, poor randomness, and inaccurate gene quantification.

c) Contamination by proteins or other insoluble impurities: This may impair the efficiency of mRNA isolation and reverse transcription in RNA- Seq magnetic beads, potentially causing library construction failure.

d) The "rRNA-free sample" still contains residual rRNA, which may result in a high rRNA proportion and insufficient valid data.

e) mRNA samples: Integrity is difficult to verify, and quality cannot be guaranteed.

f) The 5S peak height in the detection results may compromise the accuracy of effective quantification, potentially causing inaccurate downstream sampling or affecting sequencing data.

g) Excessive concentration: Inaccurate sampling may cause database construction failure

3. For Class B/C, if the partner insists on using it, the partner shall bear the corresponding responsibilities and risks.

IV Attachments

Attachment 1: Detection Results from Agilent 2100 or Fragment Analyzer

Attachment 2: Original information of samples

V Declaration

1. The test results are only related to the samples in this report.

2. The test report shall not be partially copied without the written approval of the laboratory.

Attachment 1: Detection results from Agilent 2100, Fragment Analyzer, Qsep-400, or Agilent 4200

I Pre-Testing Processing

After thawing the sample on ice, centrifuge it and mix thoroughly, then take an appropriate amount of the sample for testing.

II Testing Results

1. Sample Name: TT230411-001A

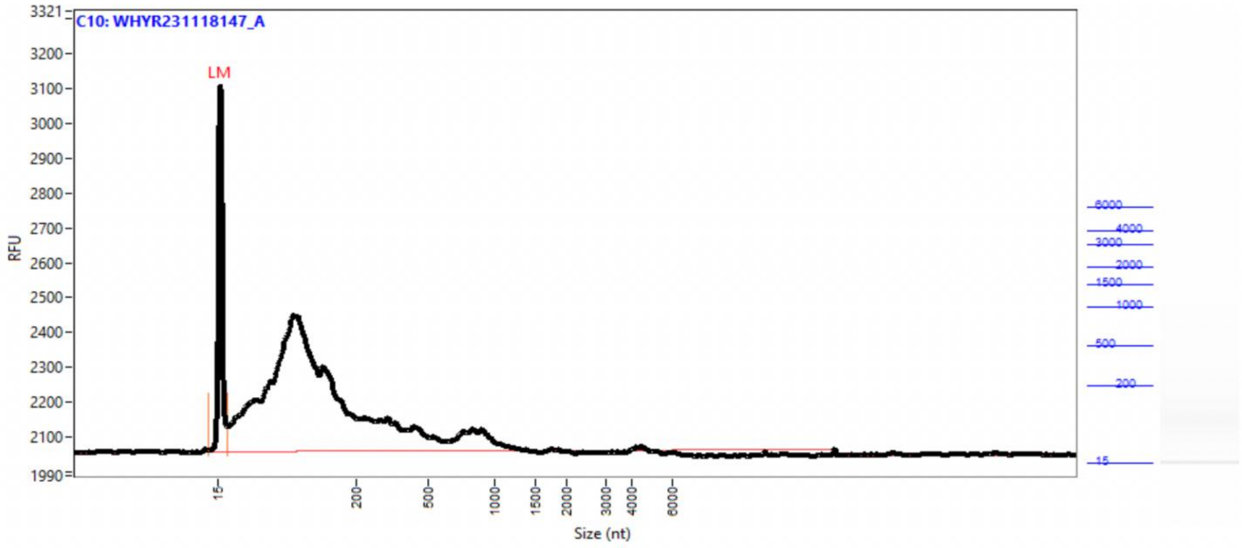

2. Sample Name: TT230411-002A

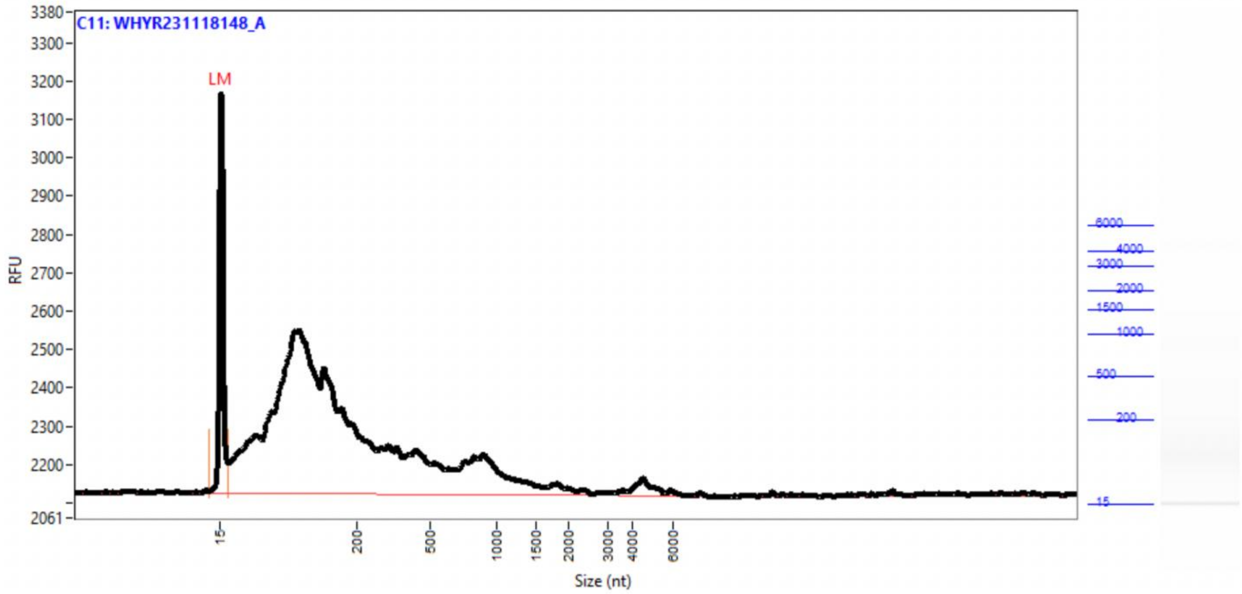

3. Sample Name: TT230411-003A

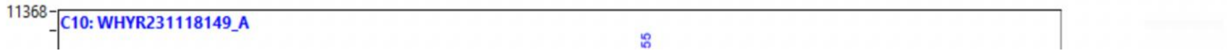

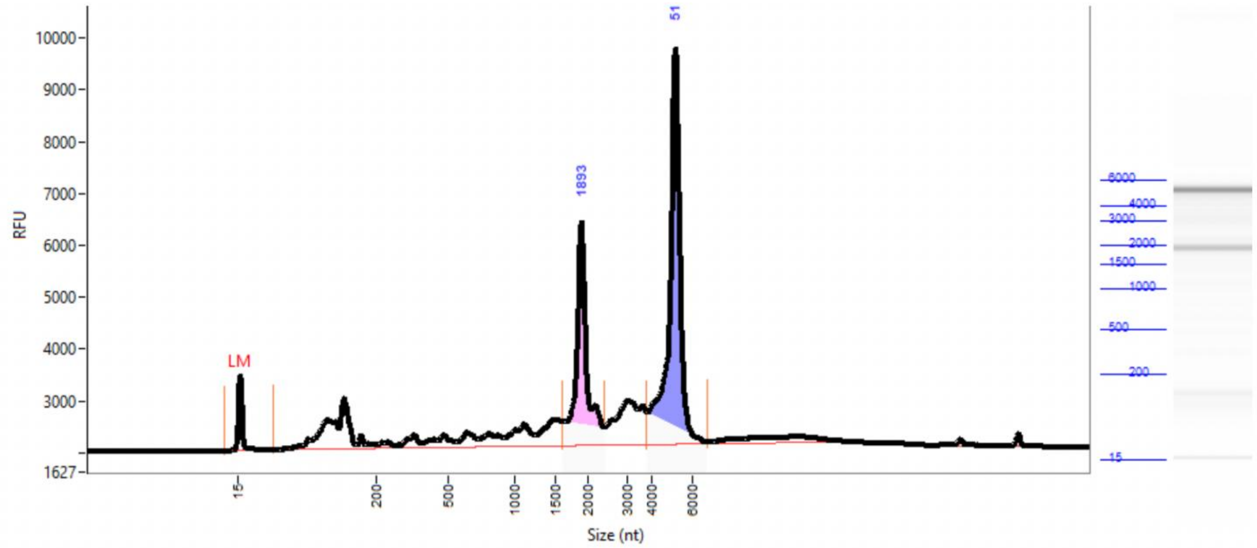

4. Sample Name: TT230411-004A

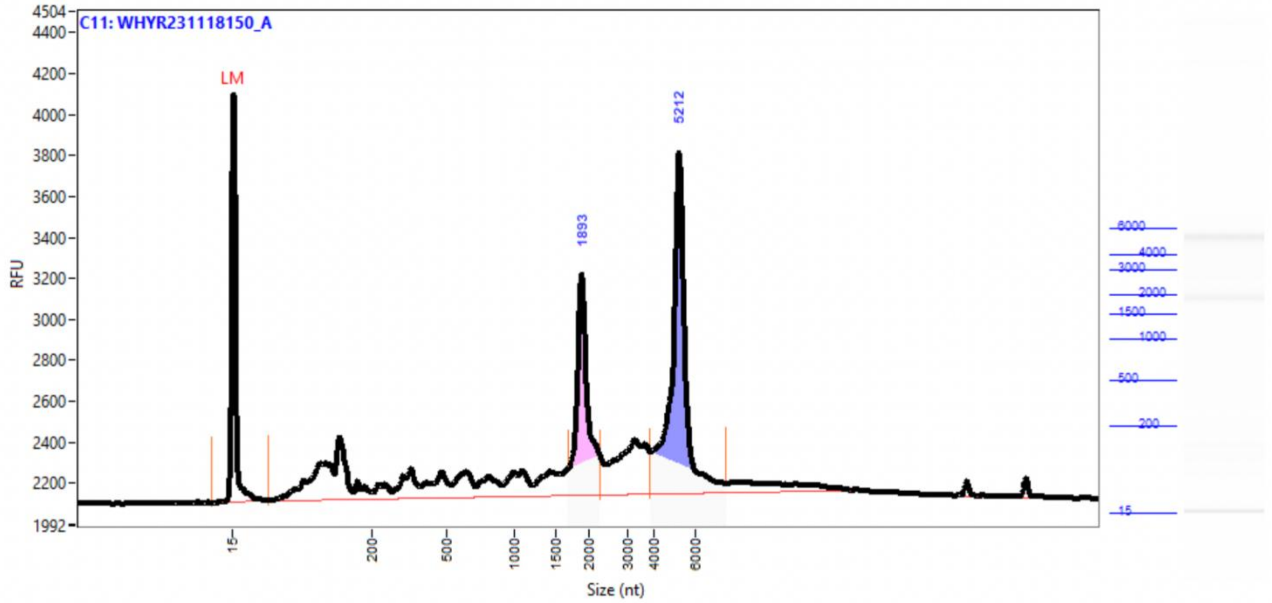

5. Sample Name: TT230411-006A

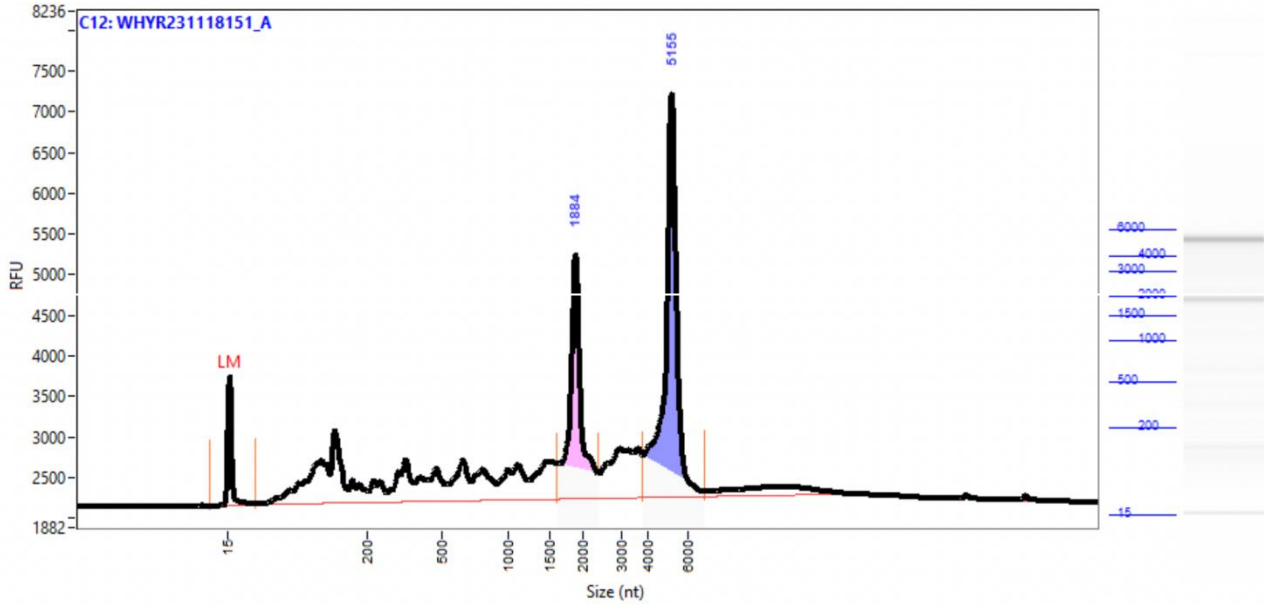

6. Sample Name: LV230413-003A

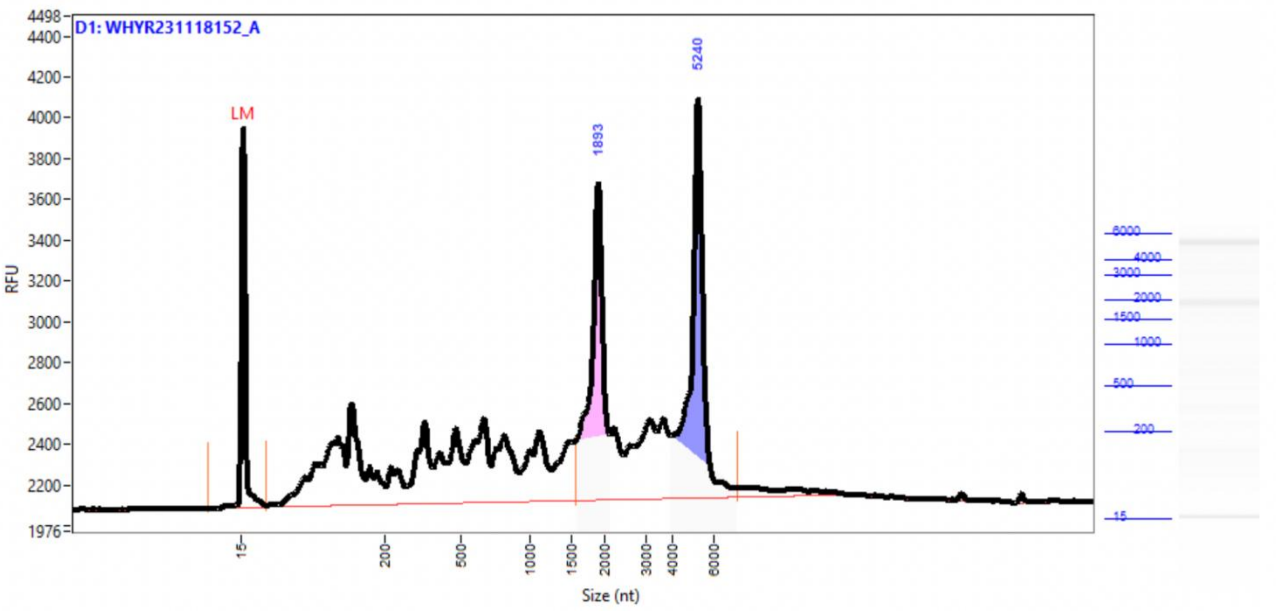

7. Sample Name: LV230413-004A

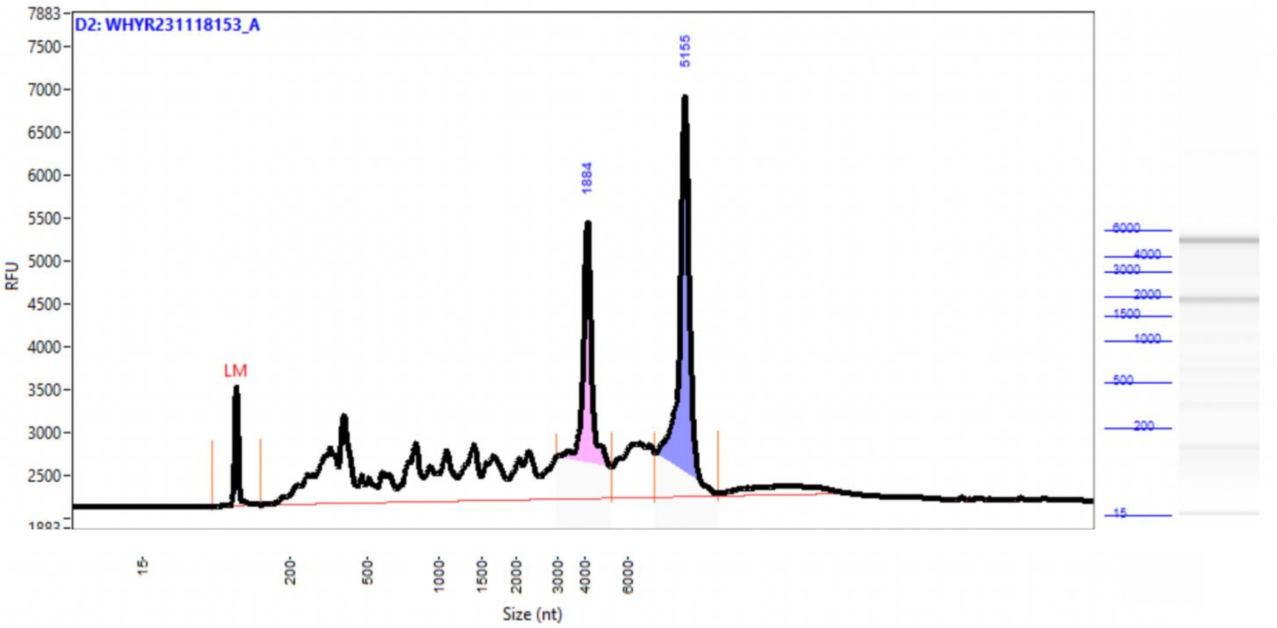

8. Sample Name: LV230413-005A

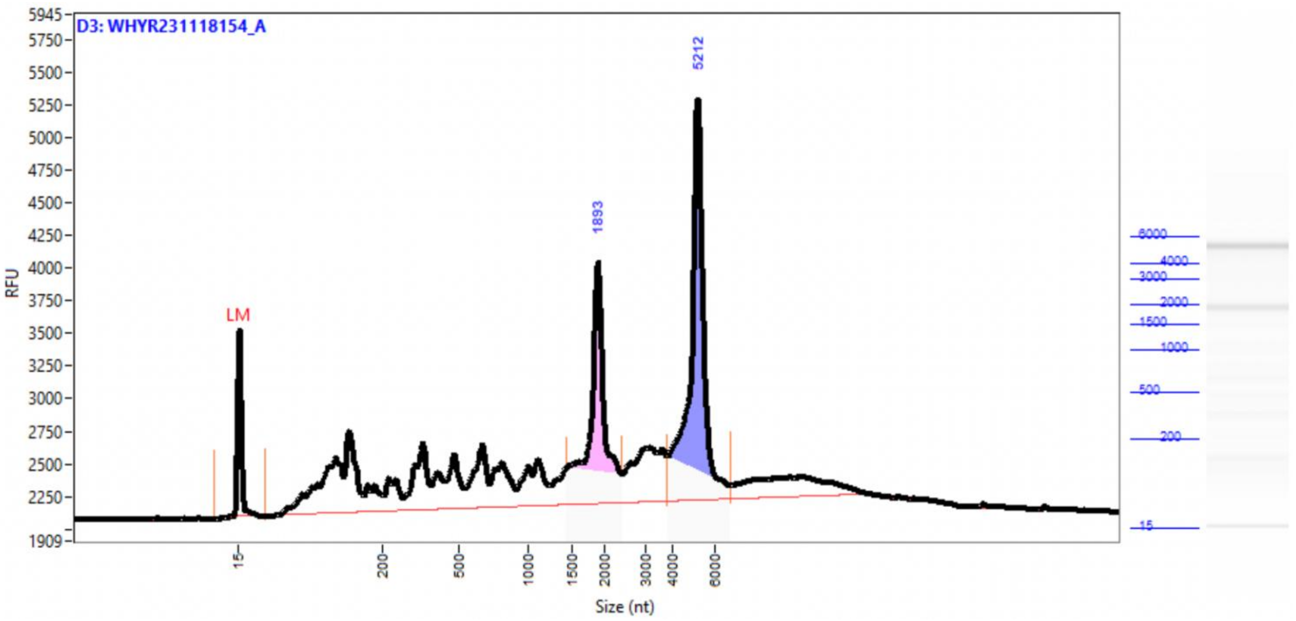

9. Sample Name: LV230413-006A

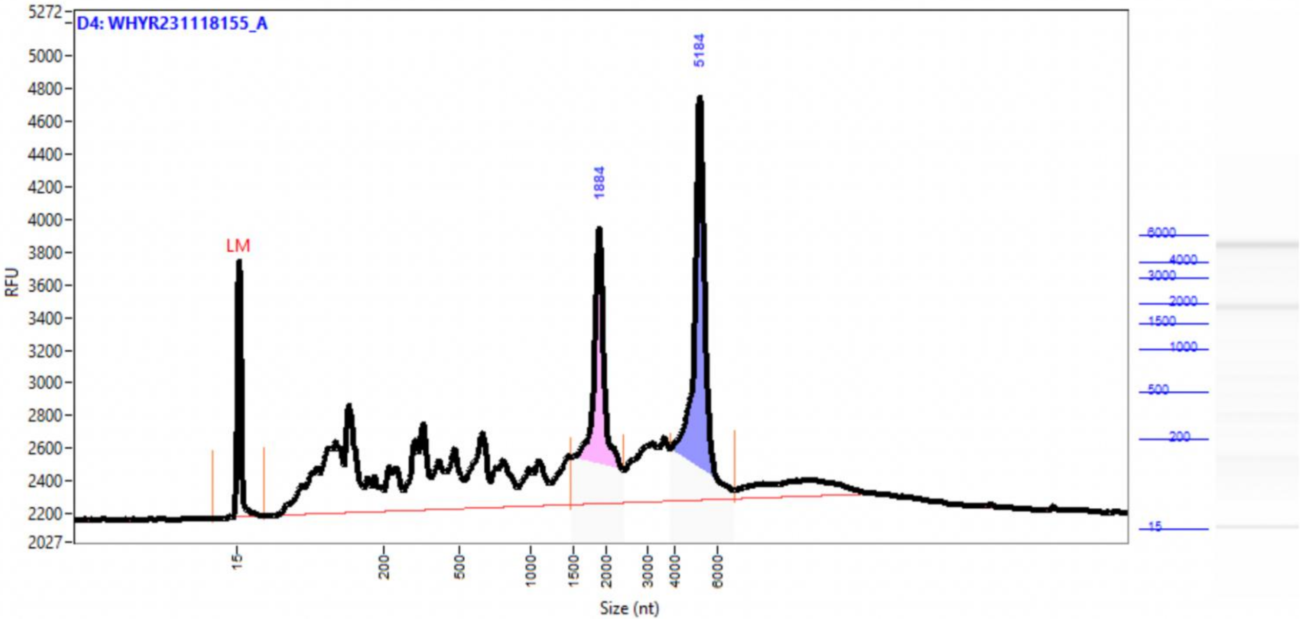

10. Sample Name: LV230414-006A

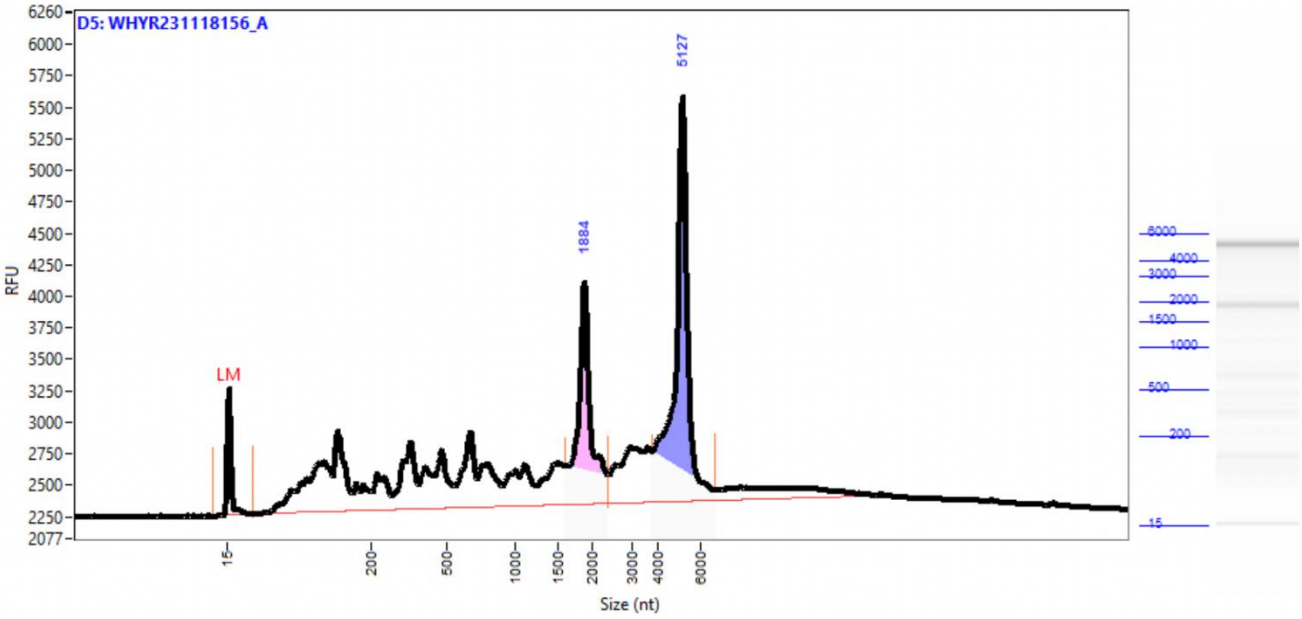

11. Sample Name: LV230417-009A

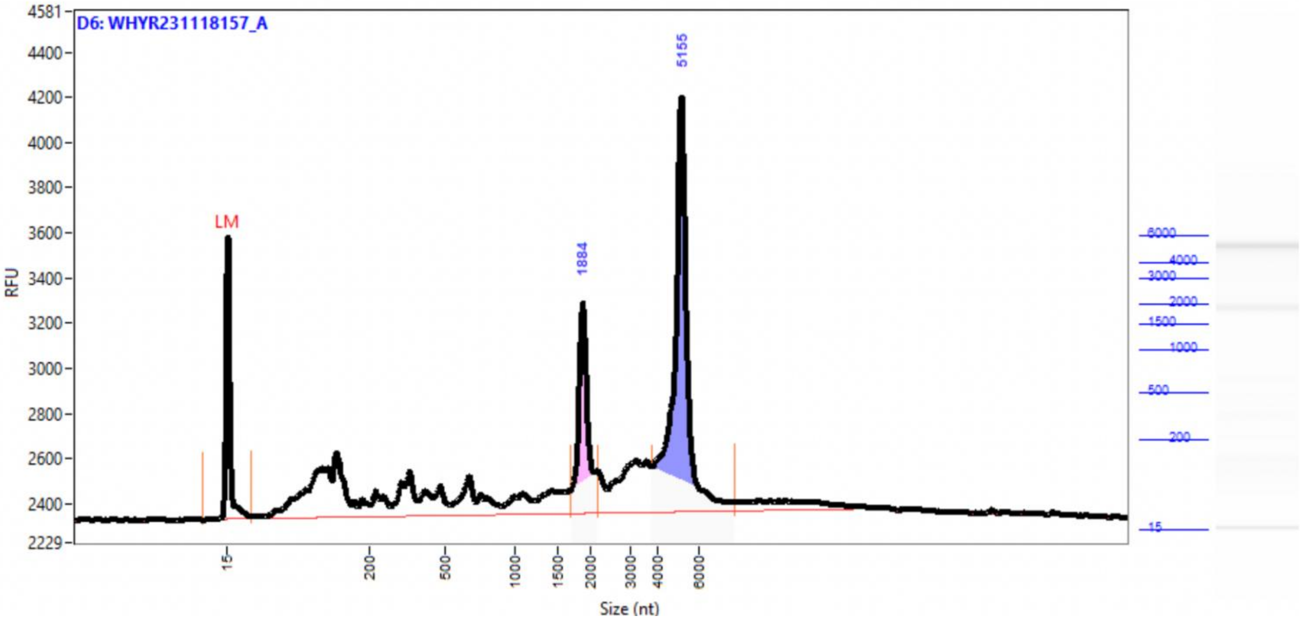

12. Sample Name: LV230601-002A

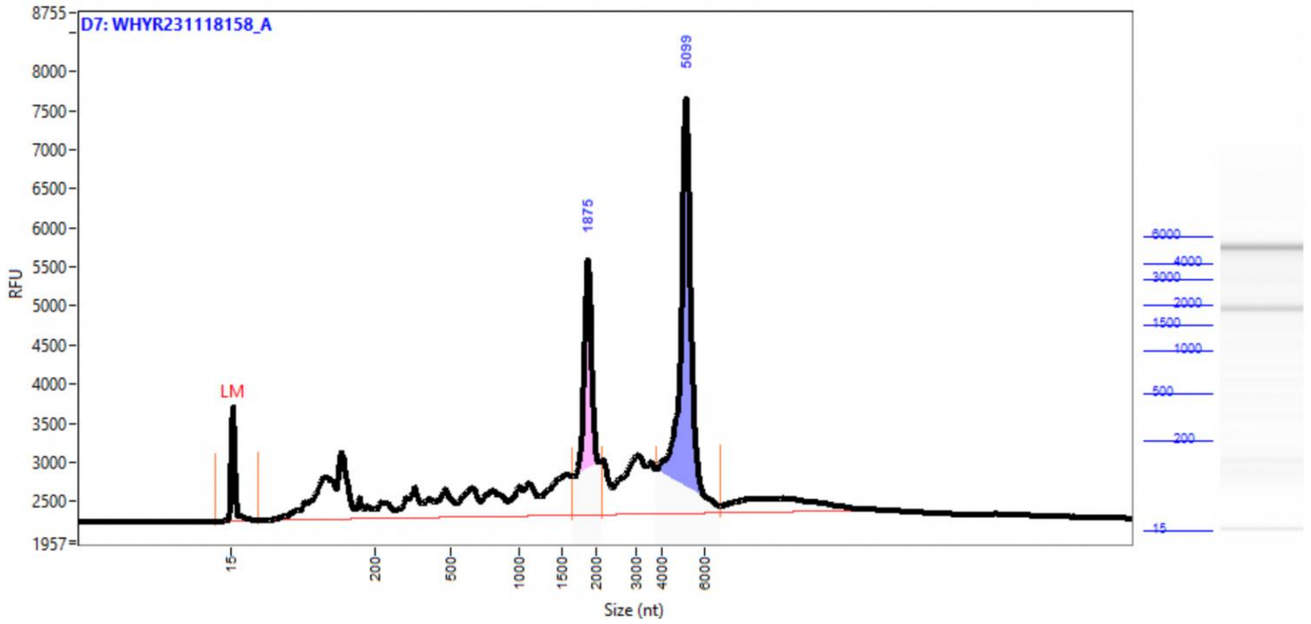

13. Sample Name: LV230825-009A

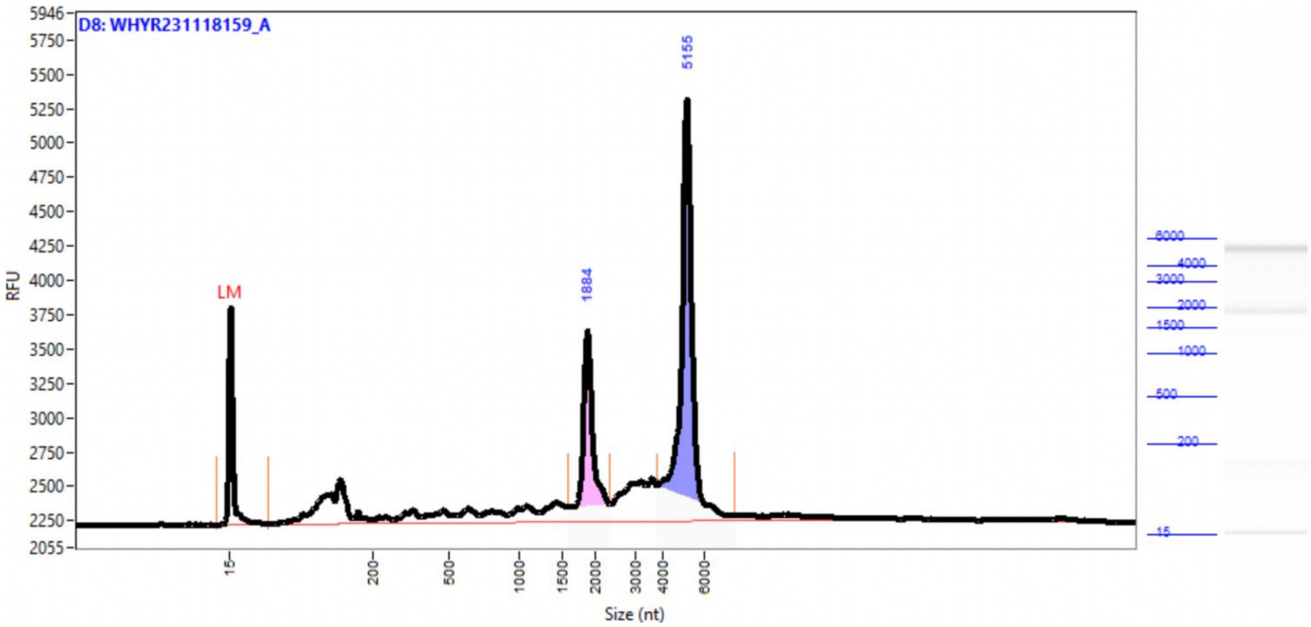

14. Sample Name: LV230826-016A

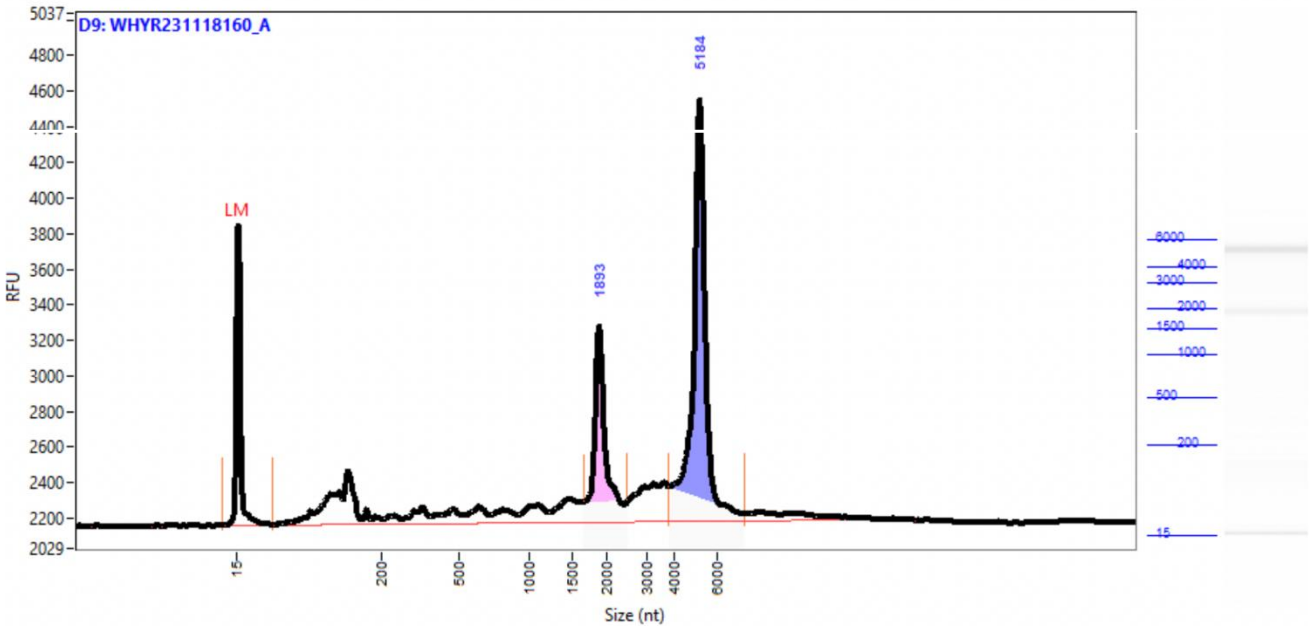

15. Sample Name: LV230829-014A

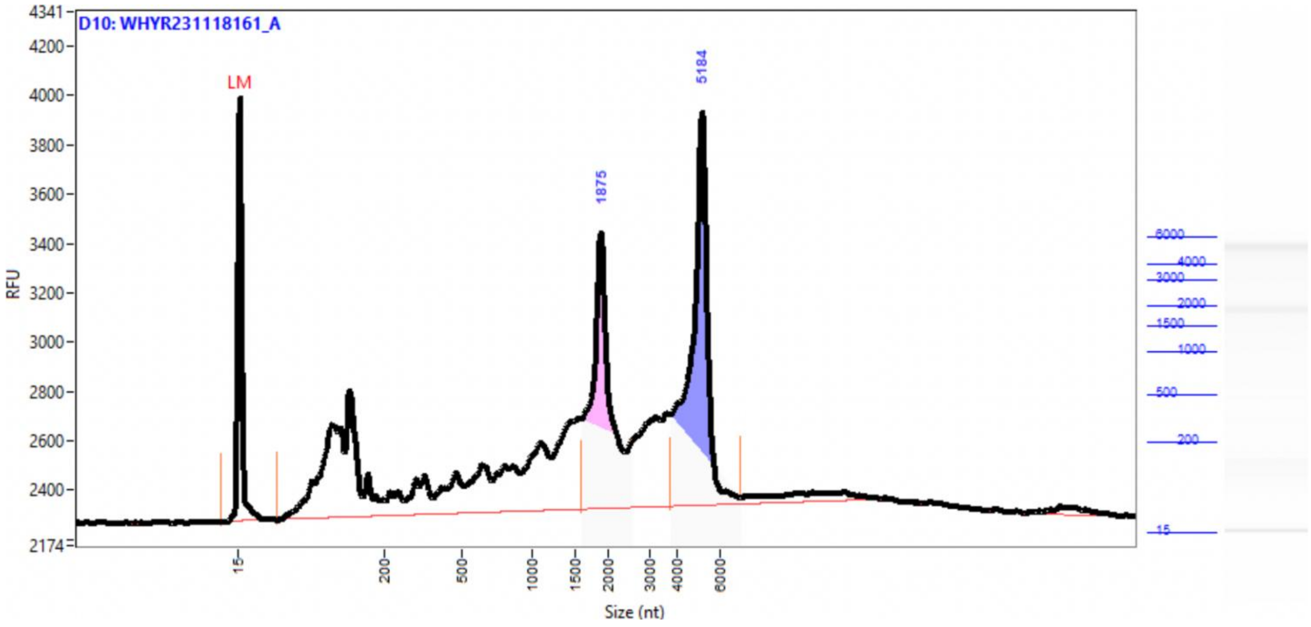

16. Sample Name: LV230829-015A

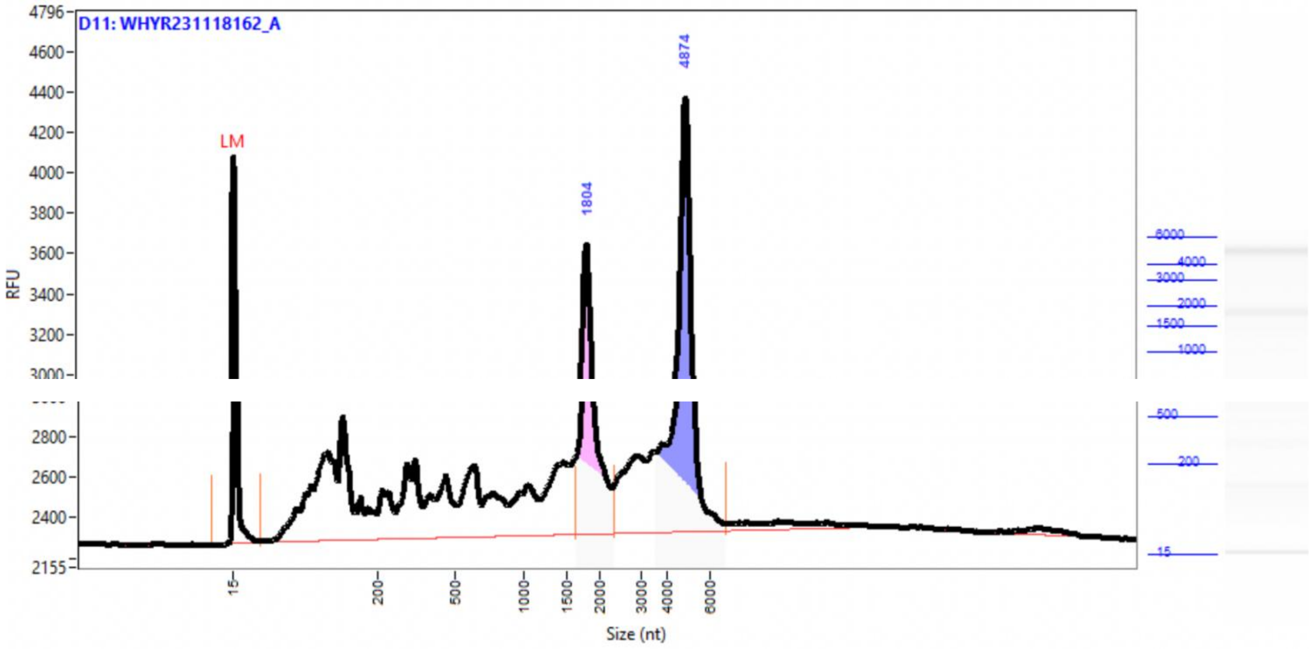

17. Sample Name: LV230829-016A

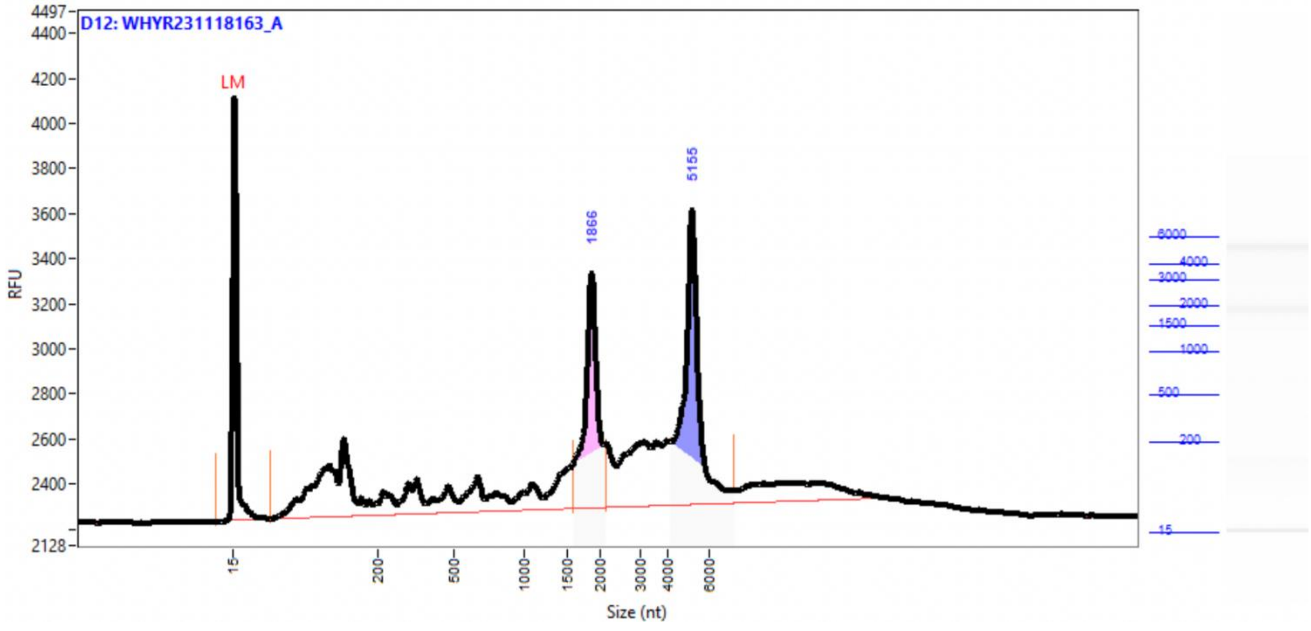

18. Sample Name: LV230830-015A

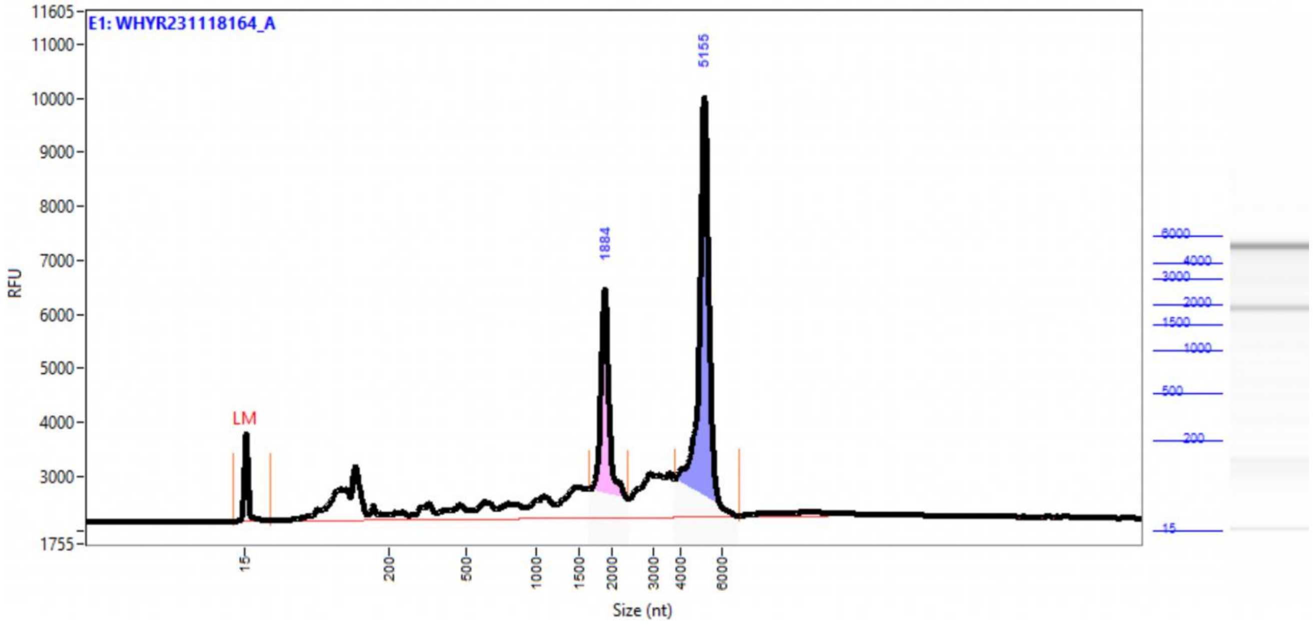

19. Sample Name: LV230831-014A

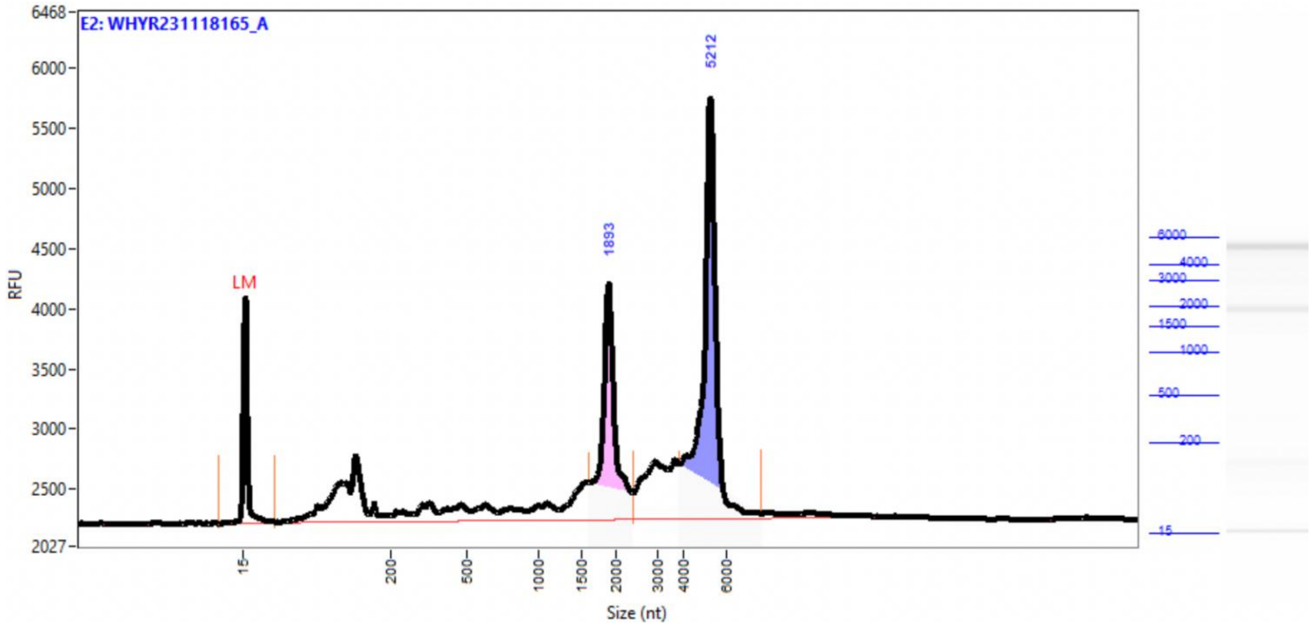

20. Sample Name: LV230904-003A

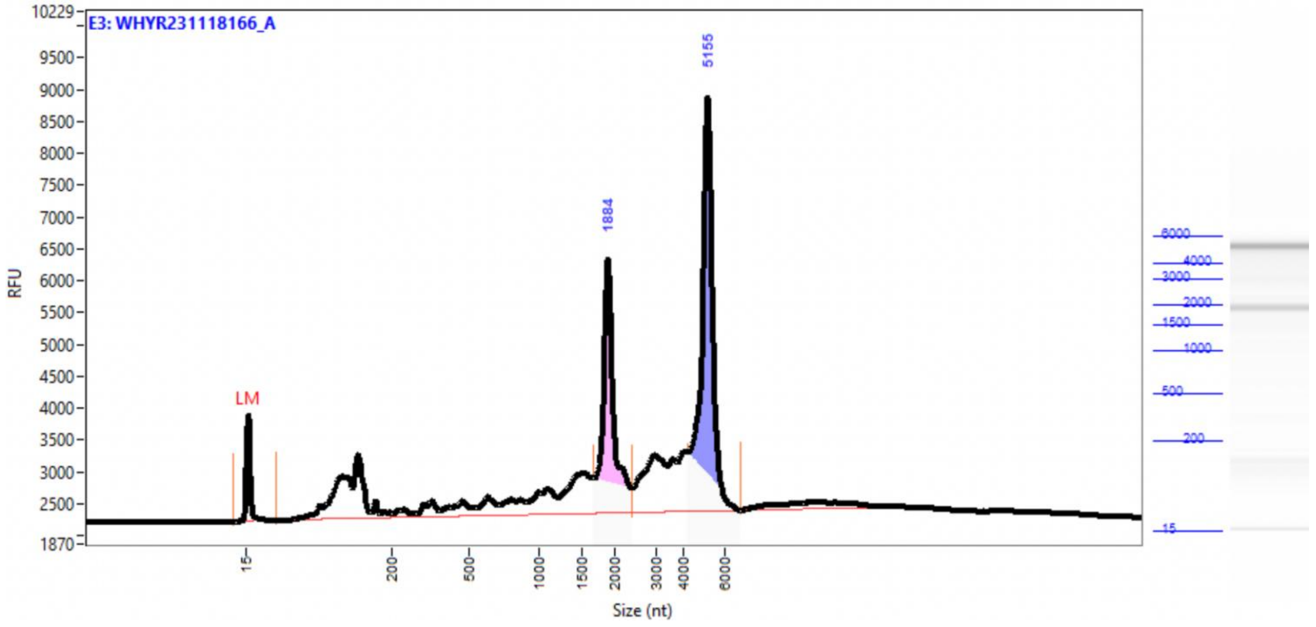

21. Sample Name: LV230912-010A

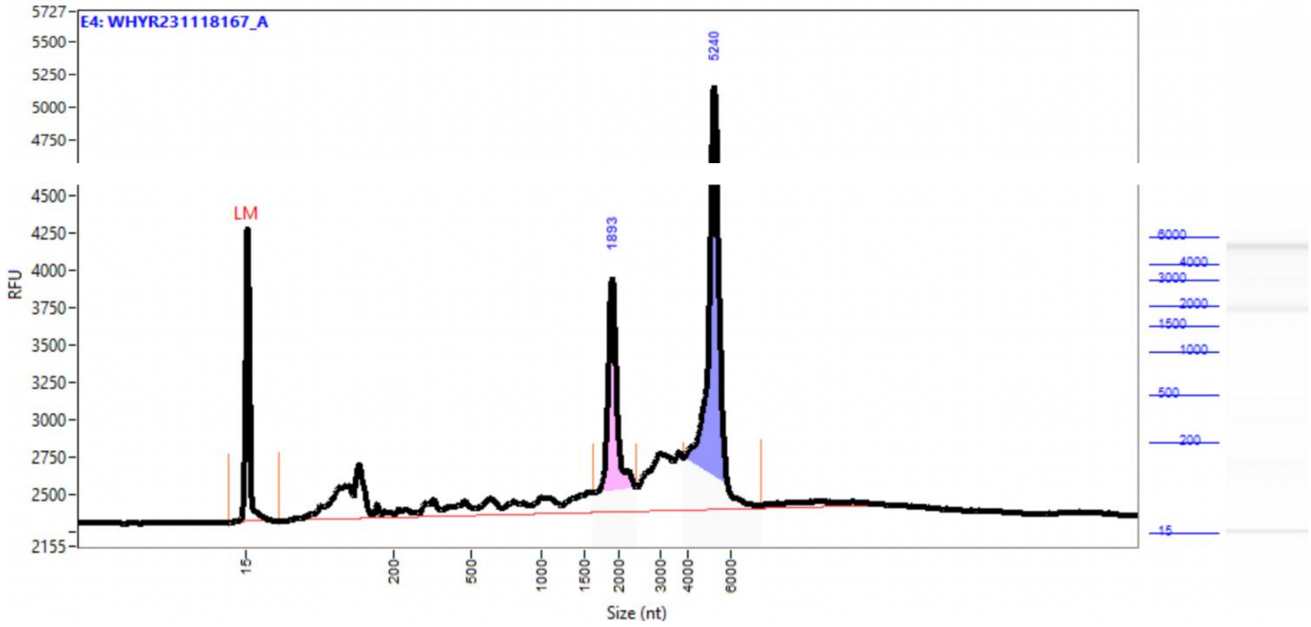

22. Sample Name: LV230927-008A

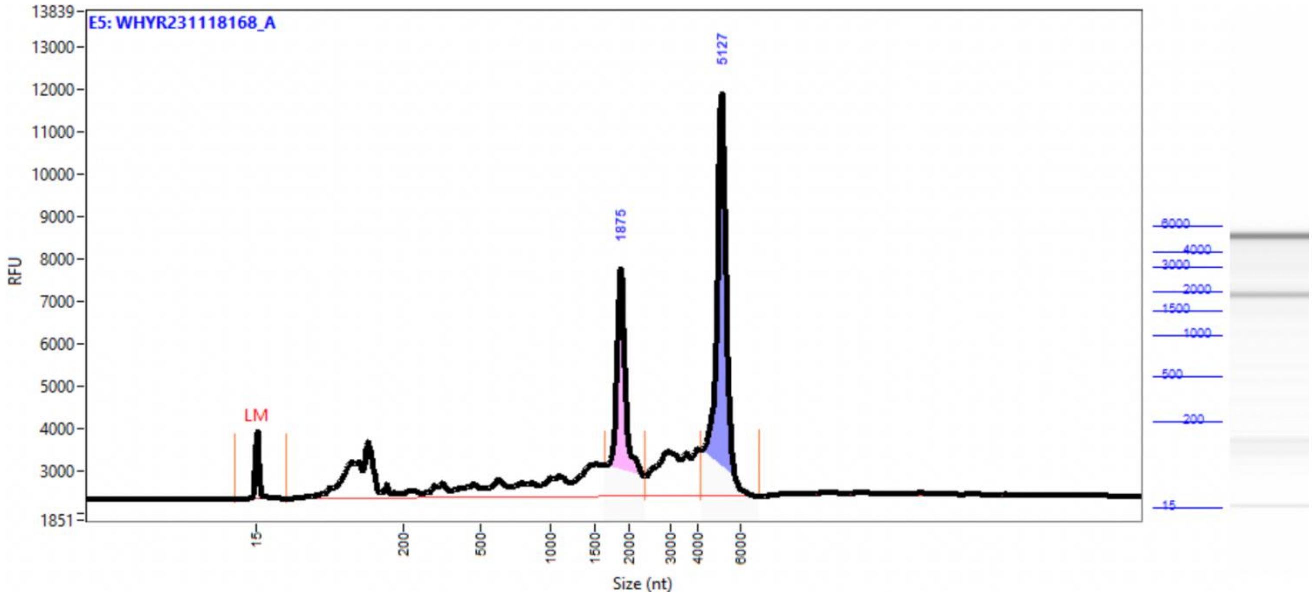

23. Sample Name: LV231020-019A

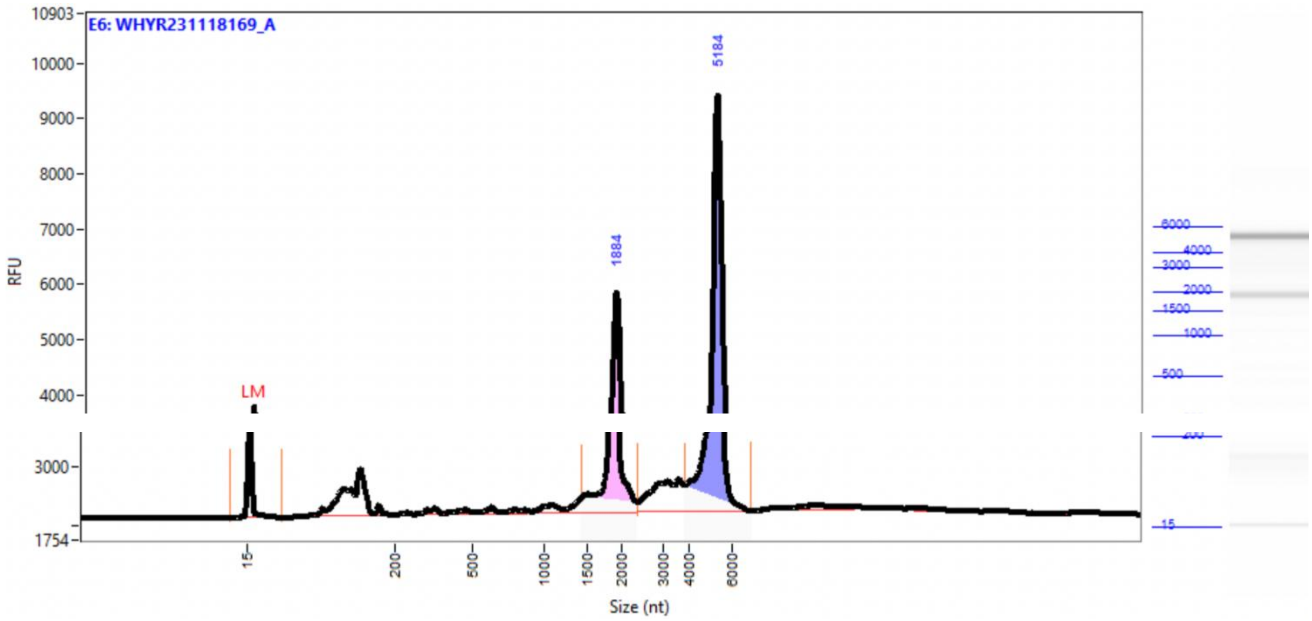

24. Sample Name: LV231023-020A

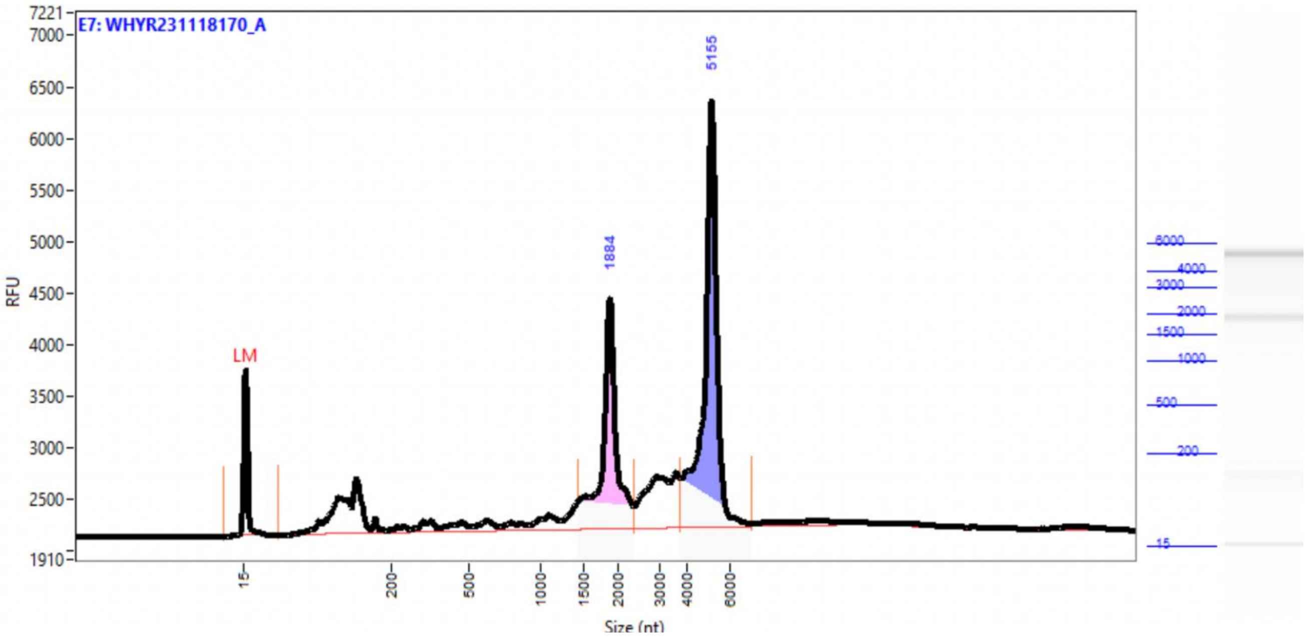

25. Sample Name: TT230411-007A

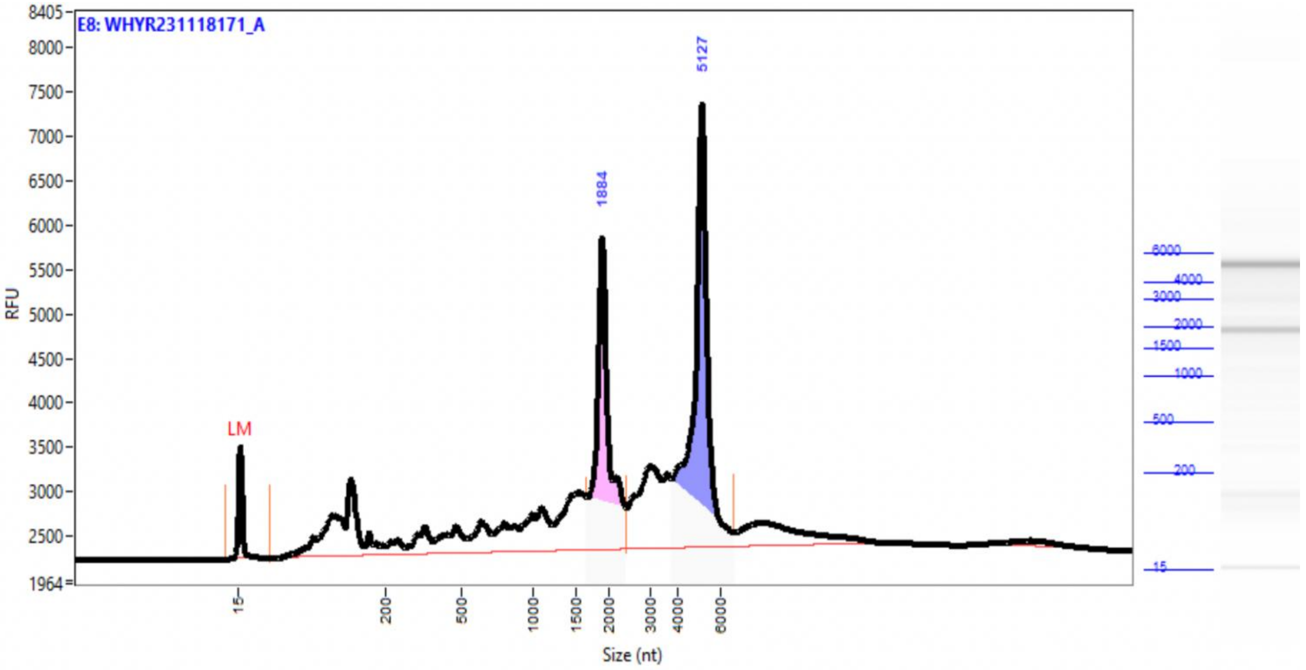

26. Sample Name: TT230411-008A

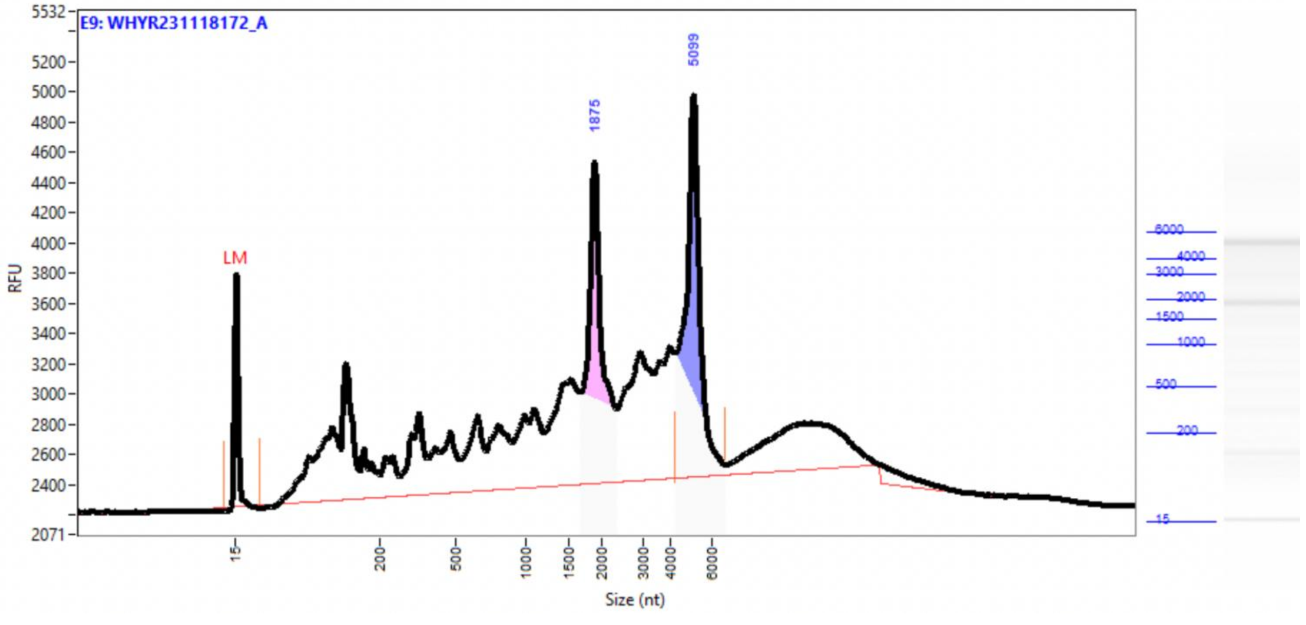

27. Sample Name: TT230411-009A

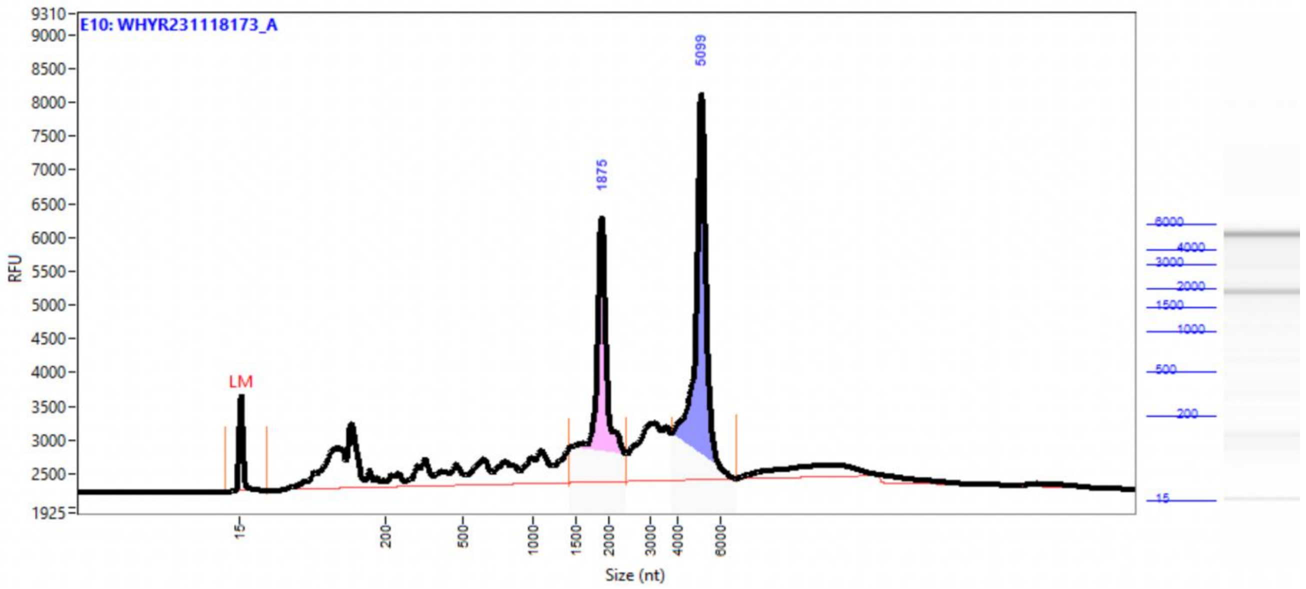

28. Sample Name: TT230411-010A

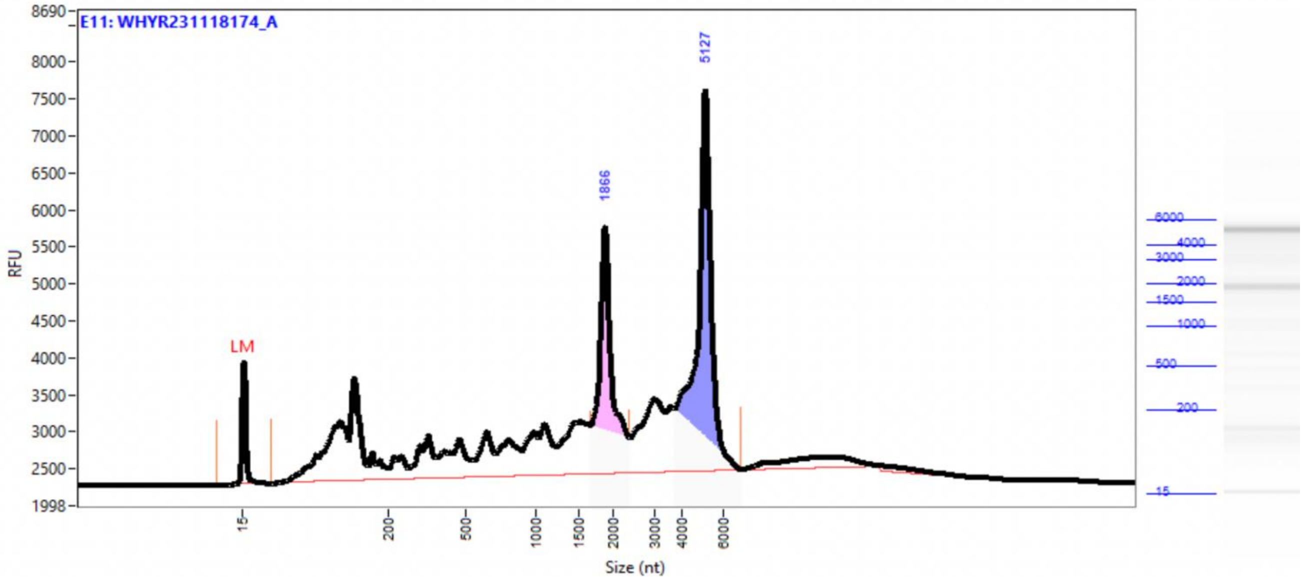

29. Sample Name: TT230411-011A

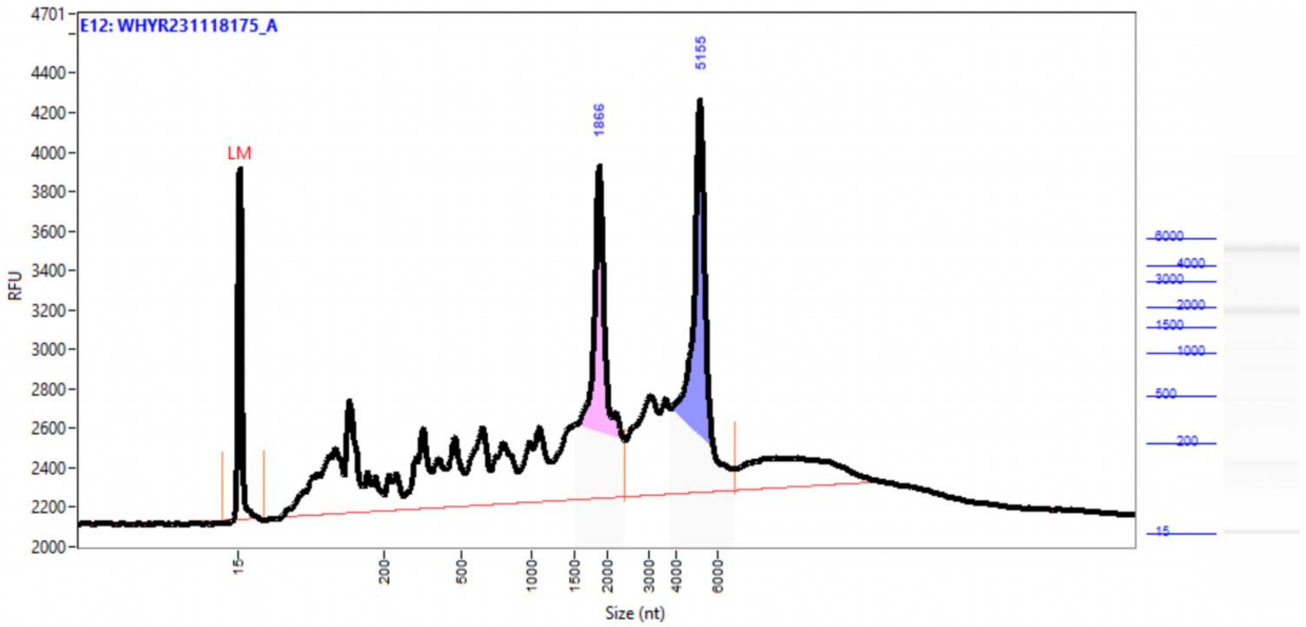

30. Sample Name: TT230411-012A

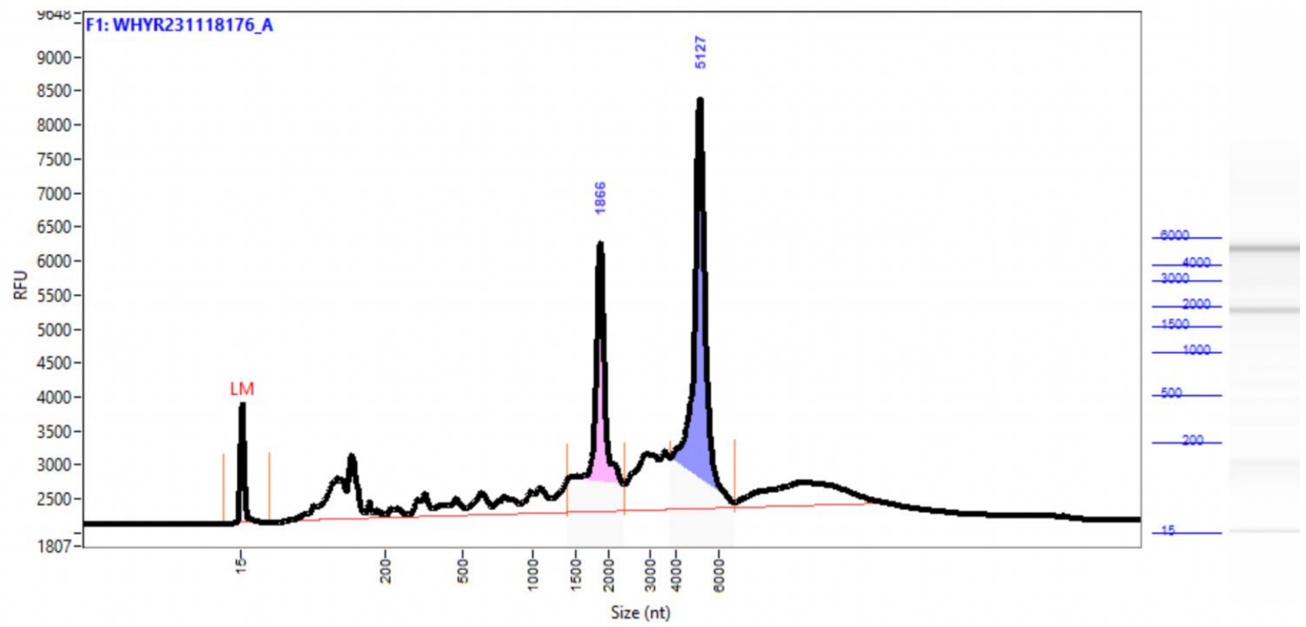

31. Sample Name: LV230412-004A

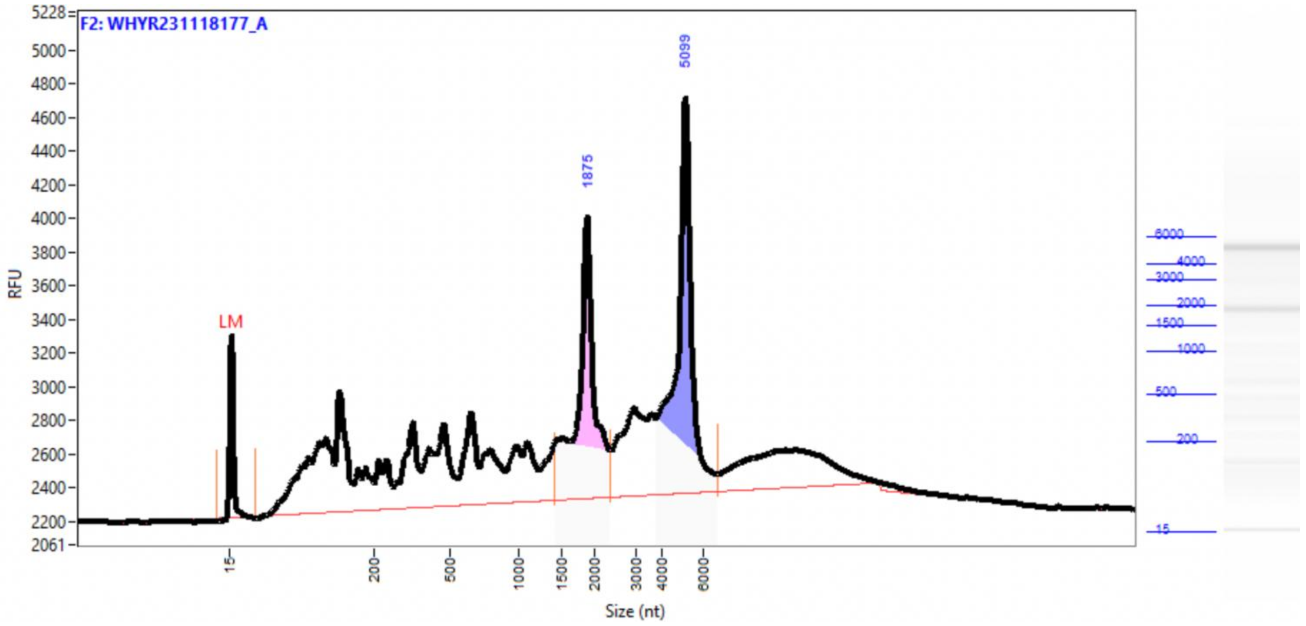

32. Sample Name: LV230421-008A

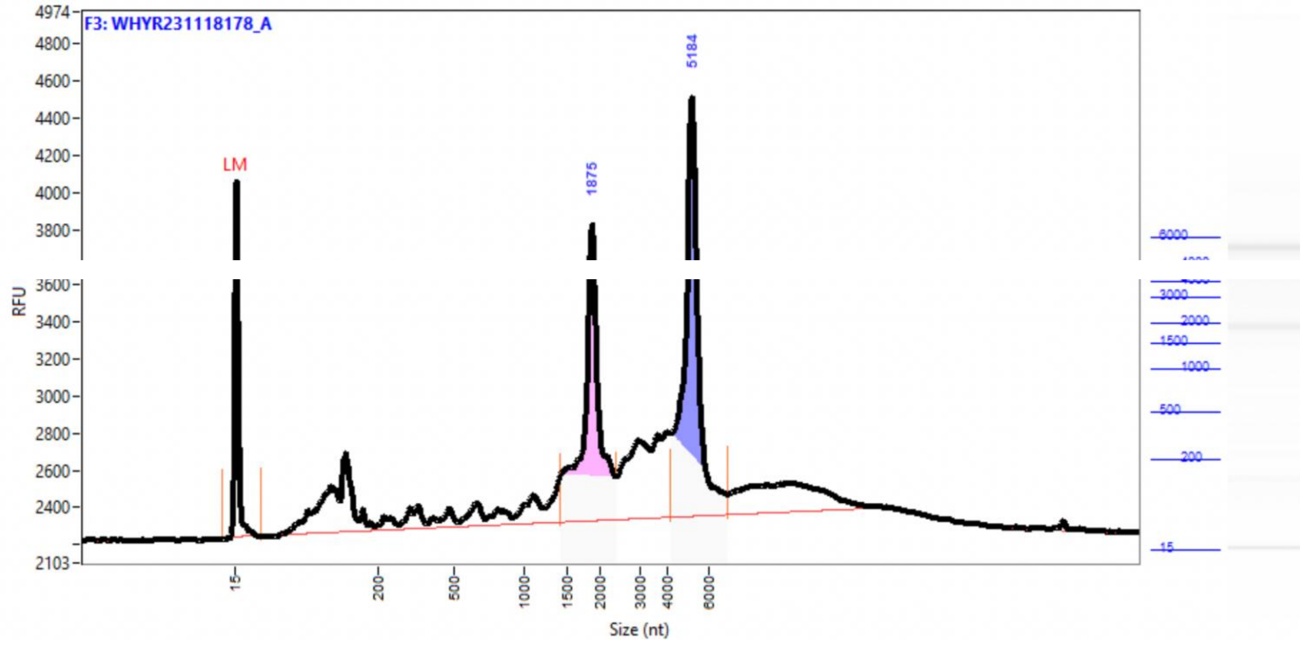

33. Sample Name: LV230513-012A

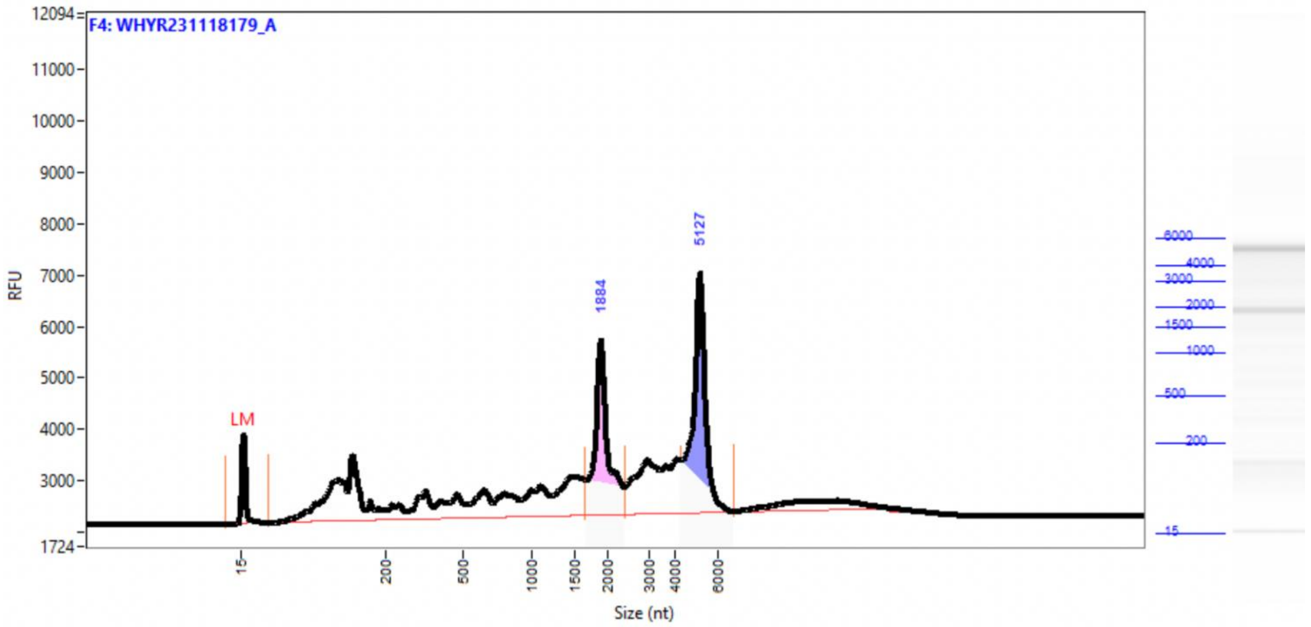

34. Sample Name: LV230513-013A

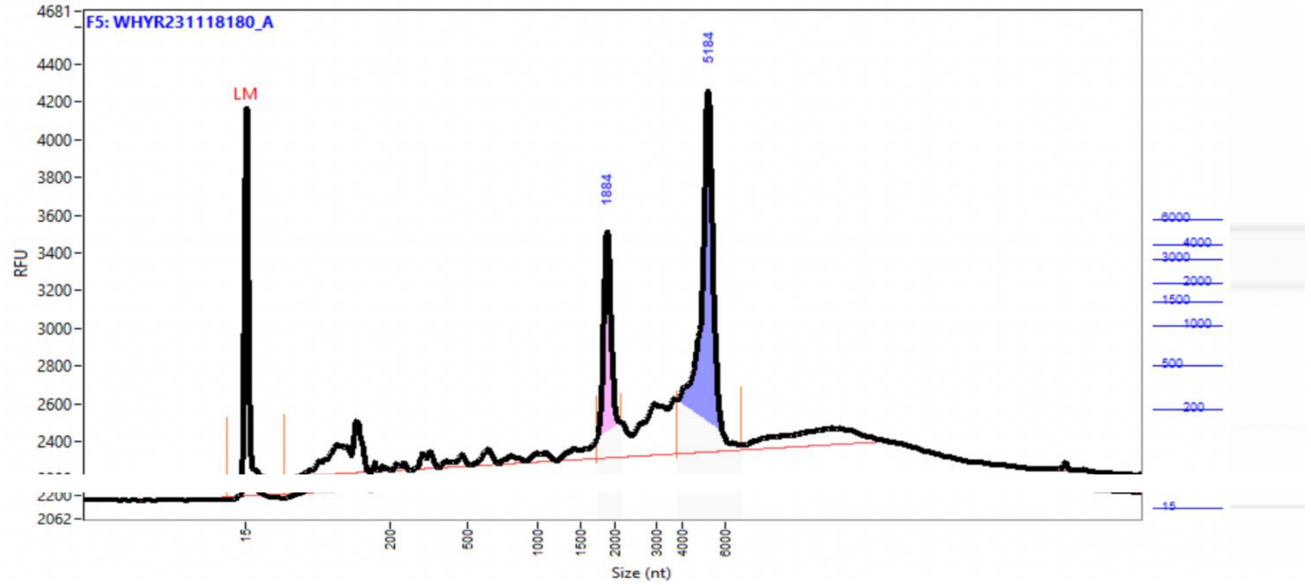

35. Sample Name: LV230513-014A

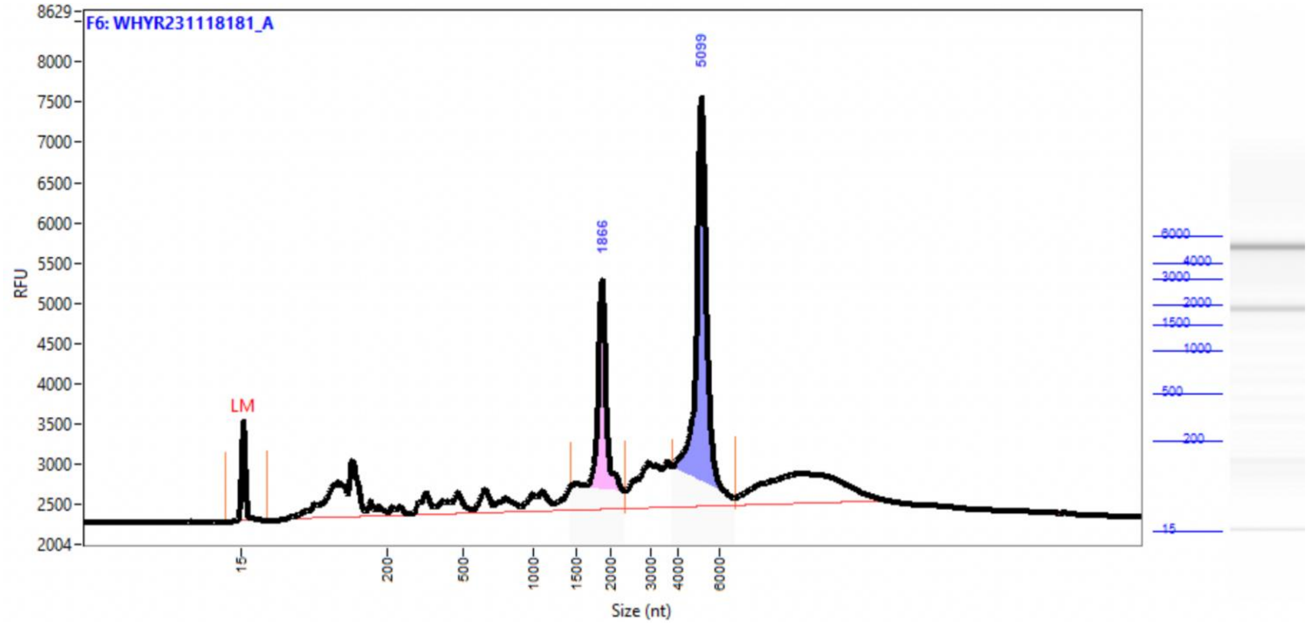

36. Sample Name: LV230602-004A

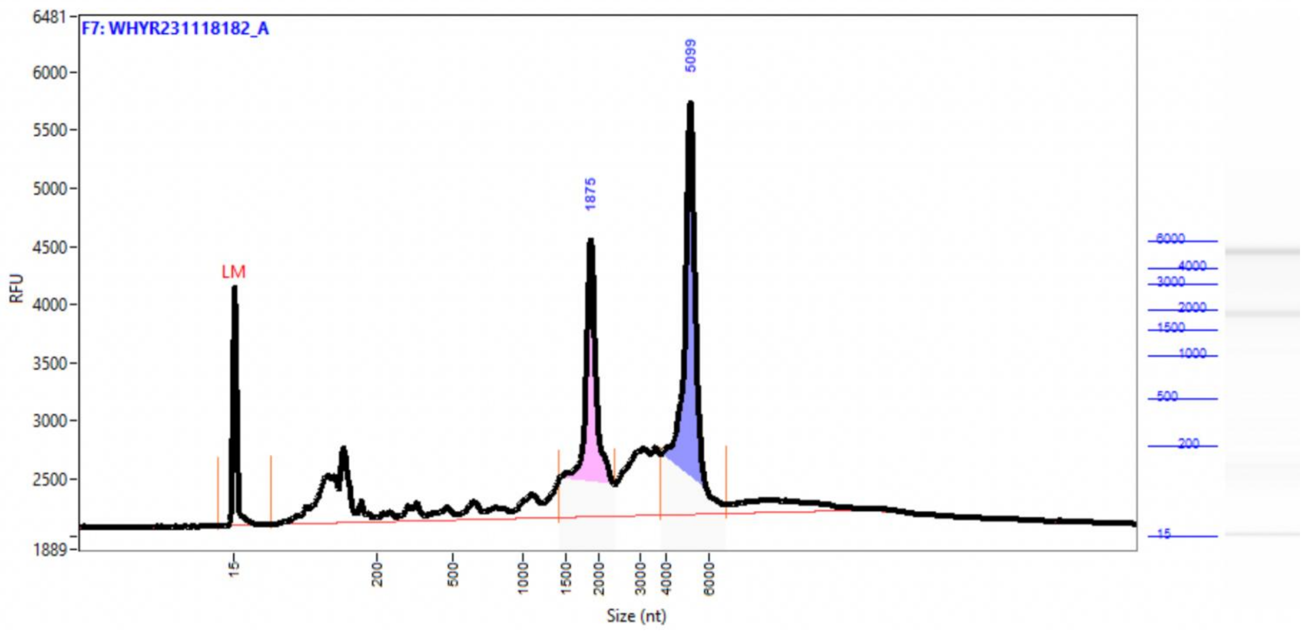

37. Sample Name: LV230904-004A

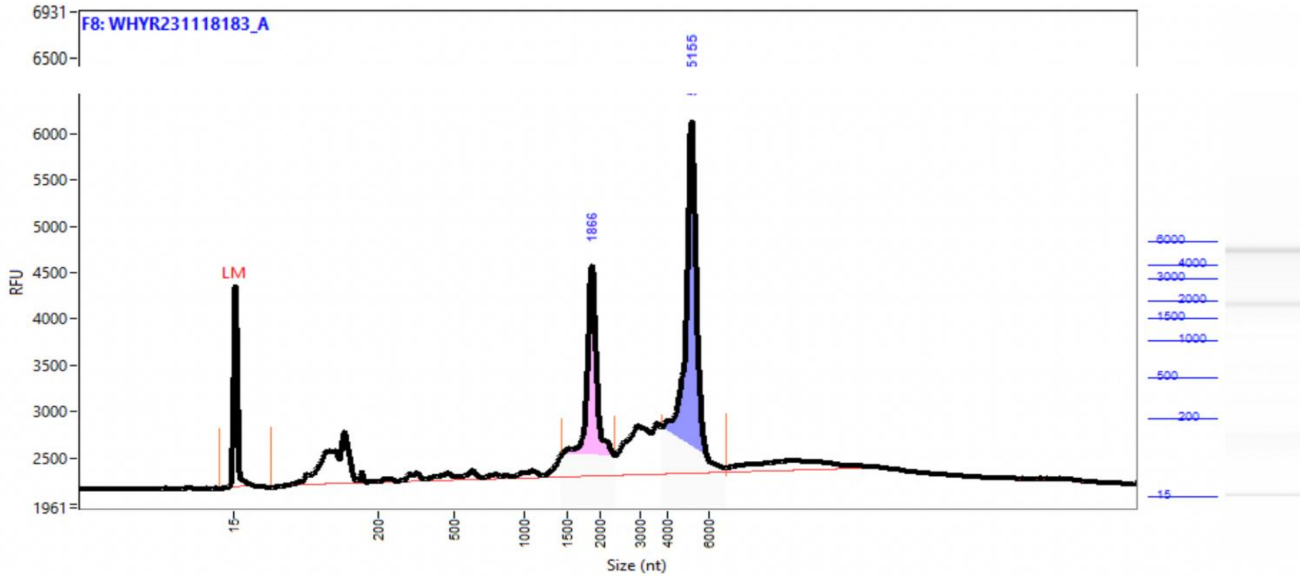

38. Sample Name: LV230906-001A

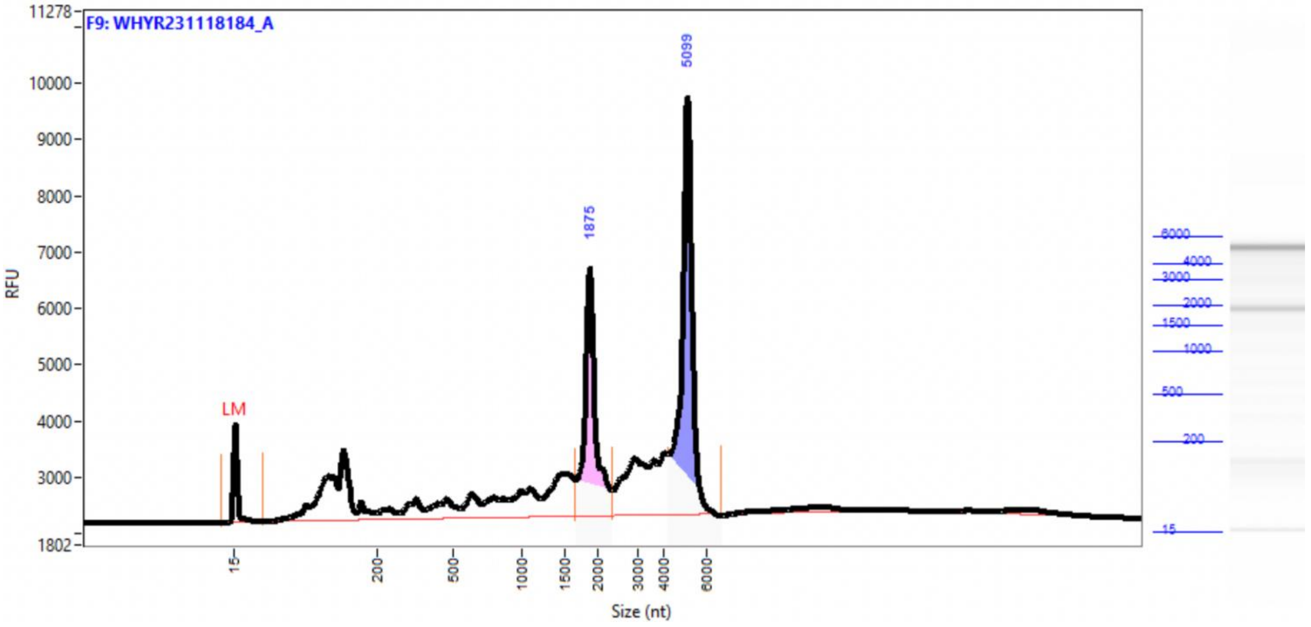

39. Sample Name: LV230906-002A

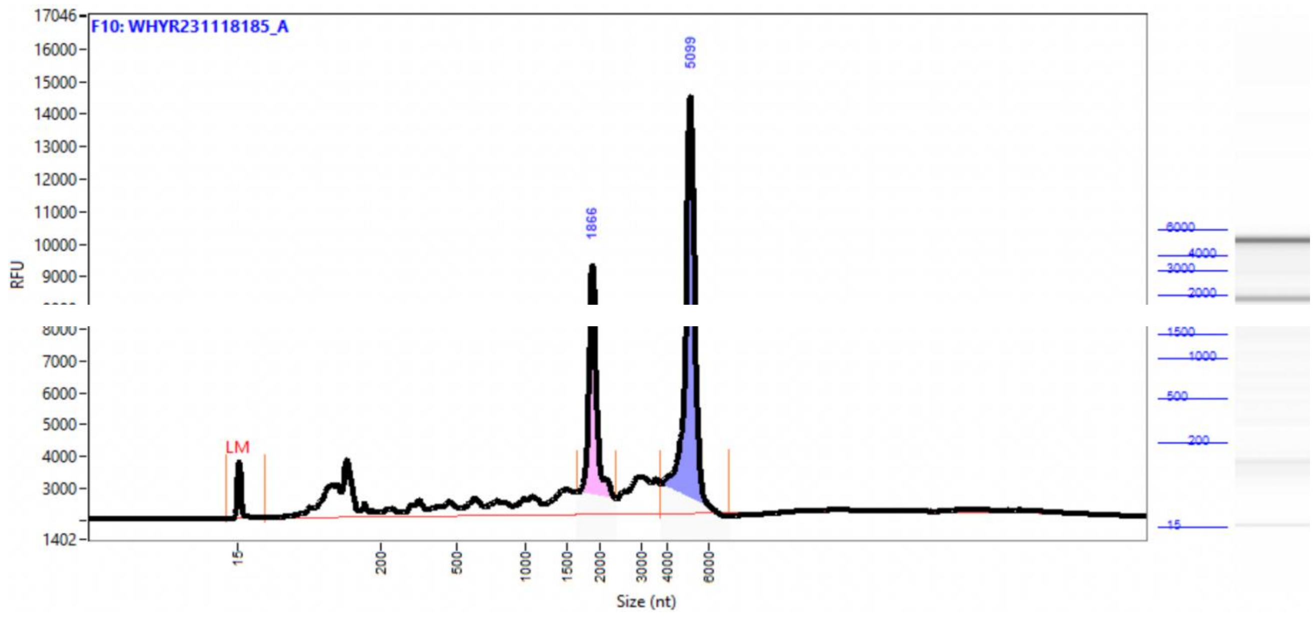

40. Sample Name: LV230907-008A

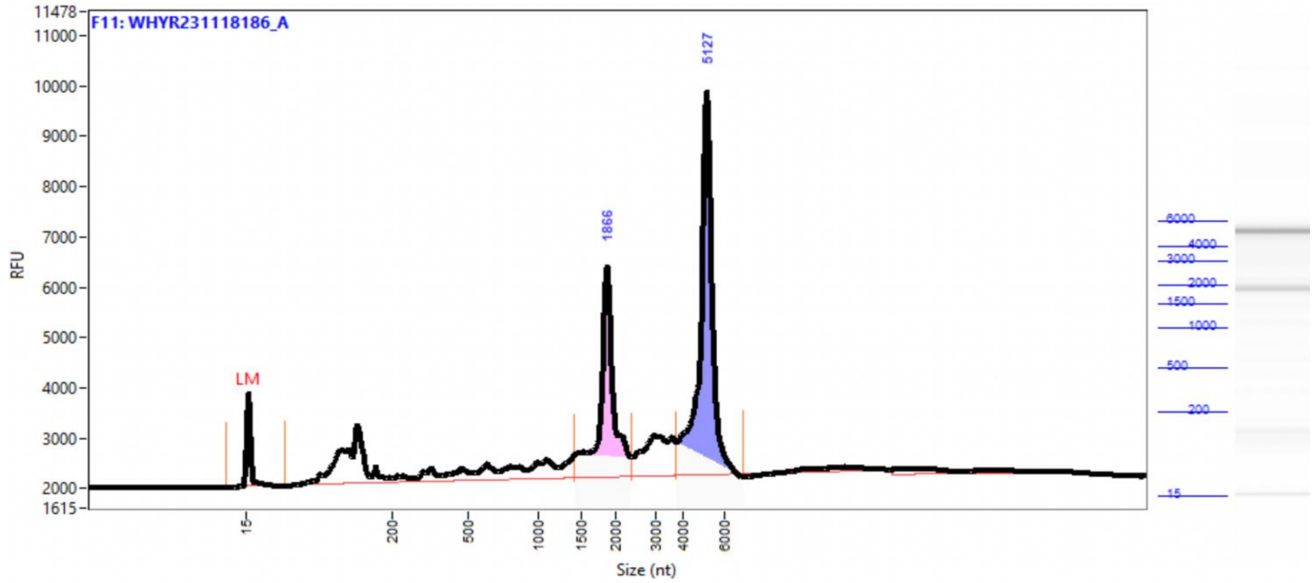

41. Sample Name: LV230907-009A

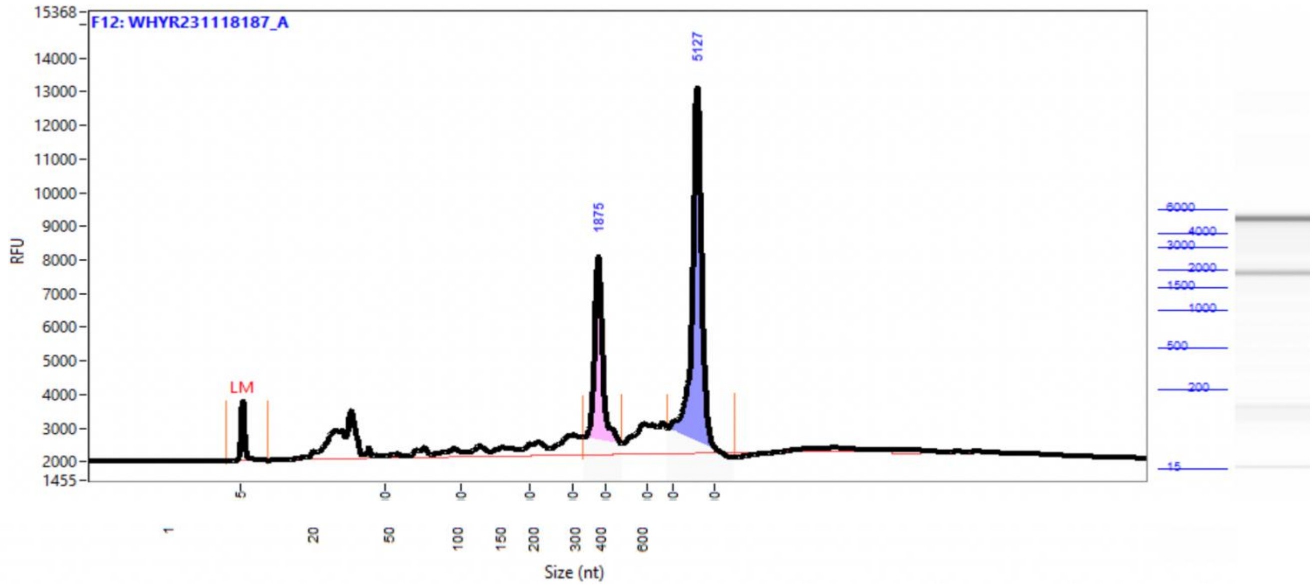

42. Sample Name: LV230907-010A

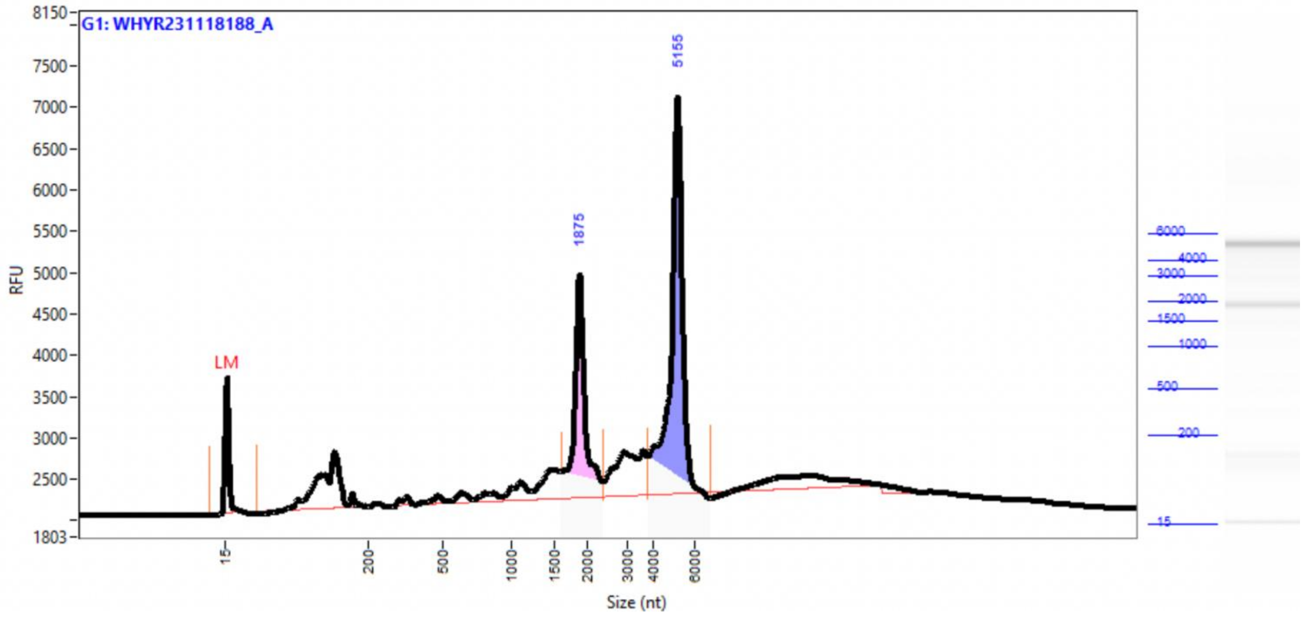

43. Sample Name: LV230907-011A

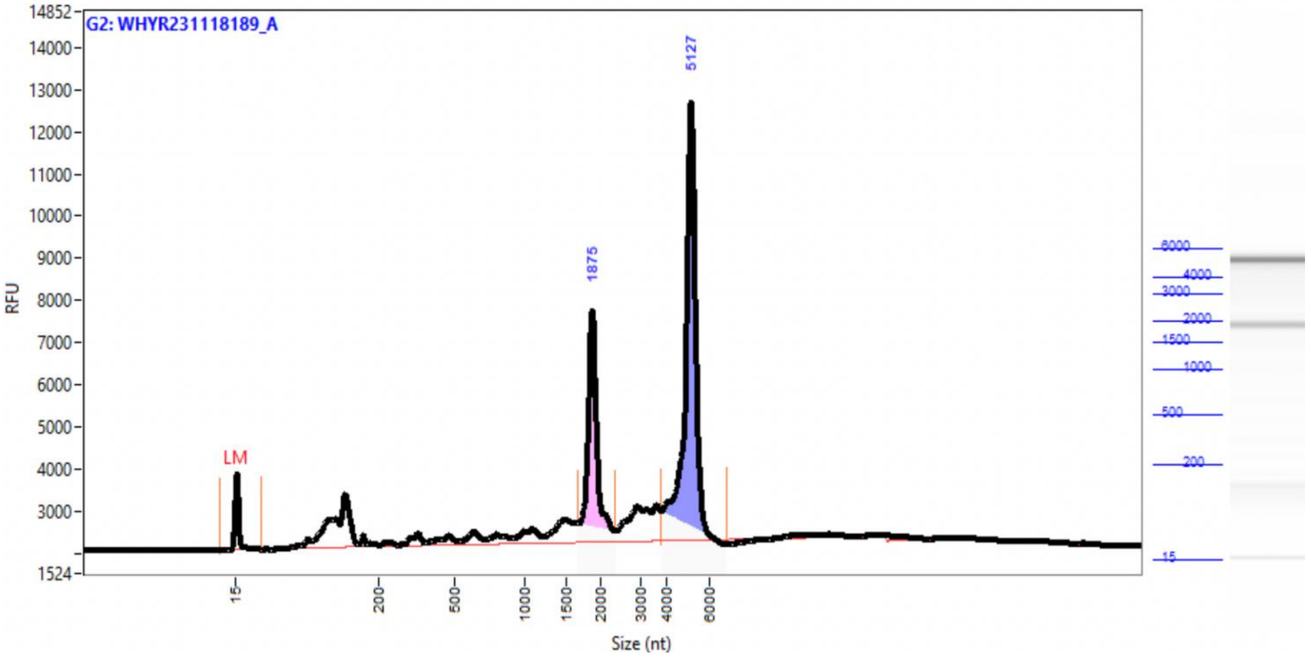

44. Sample Name: LV230908-011A

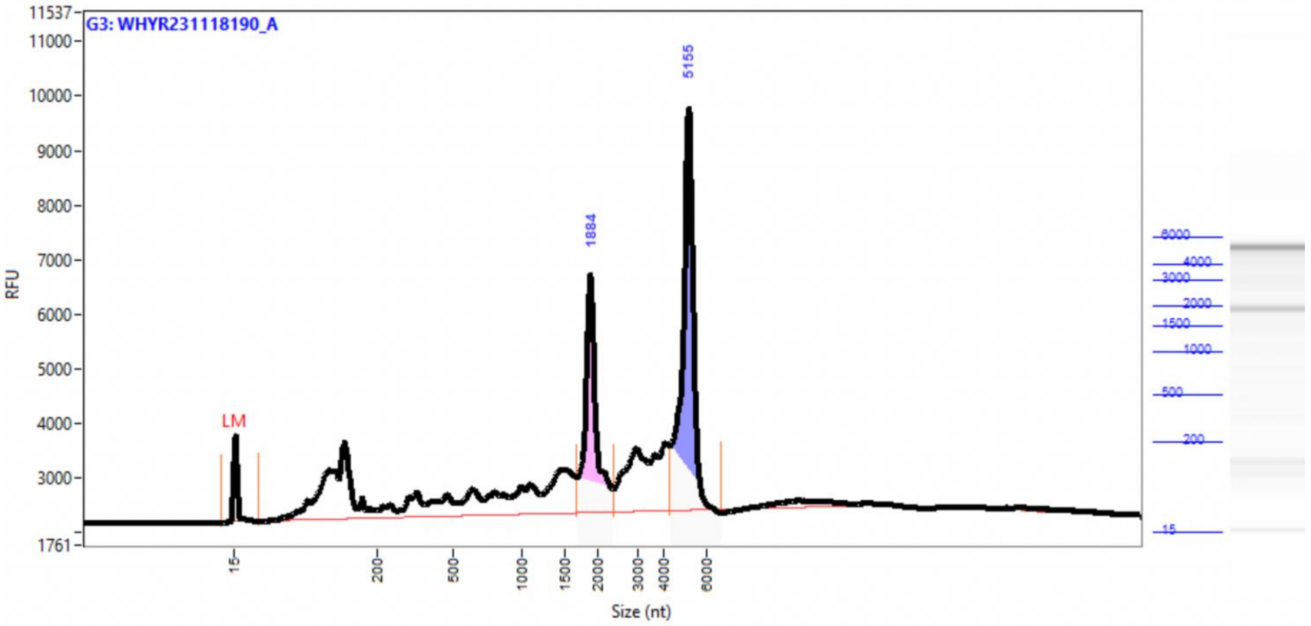

45. Sample Name: LV230908-012A

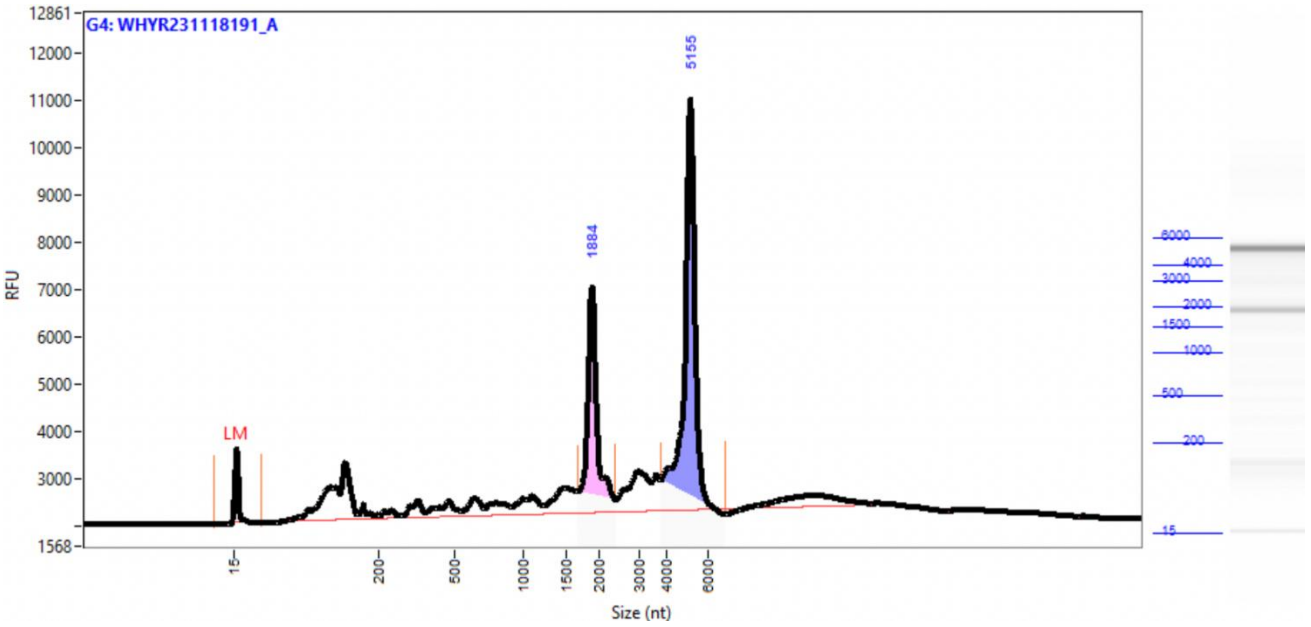

46. Sample Name: LV230912-009A

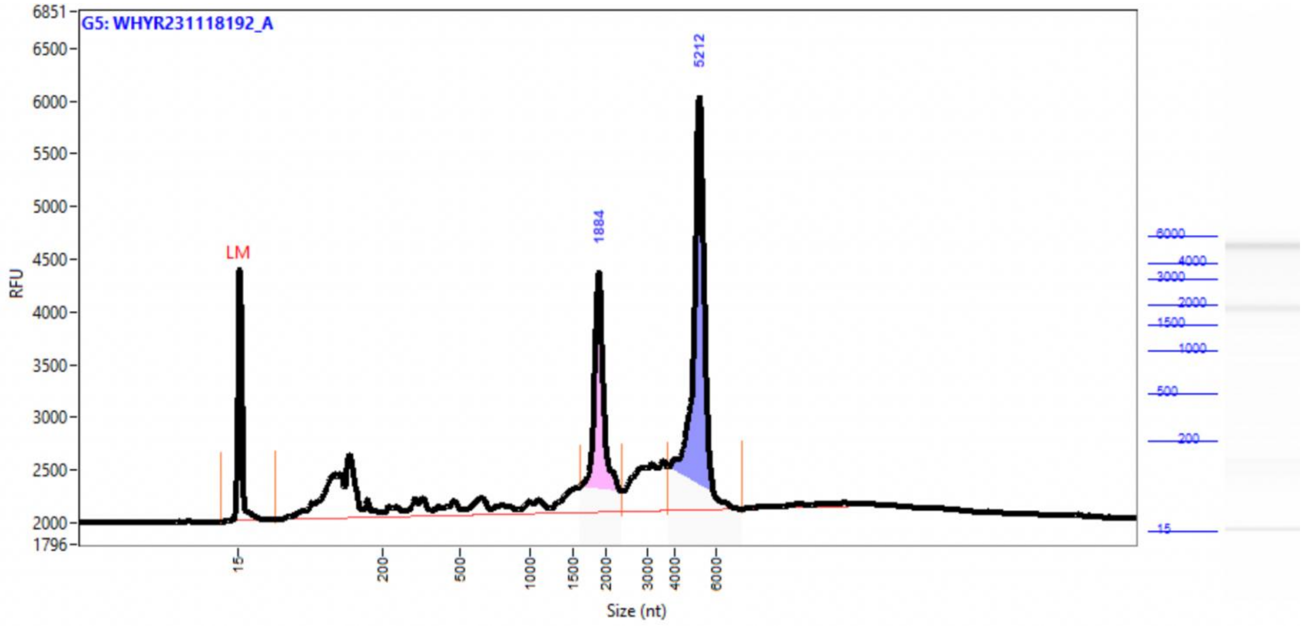

47. Sample Name: LV231018-006A

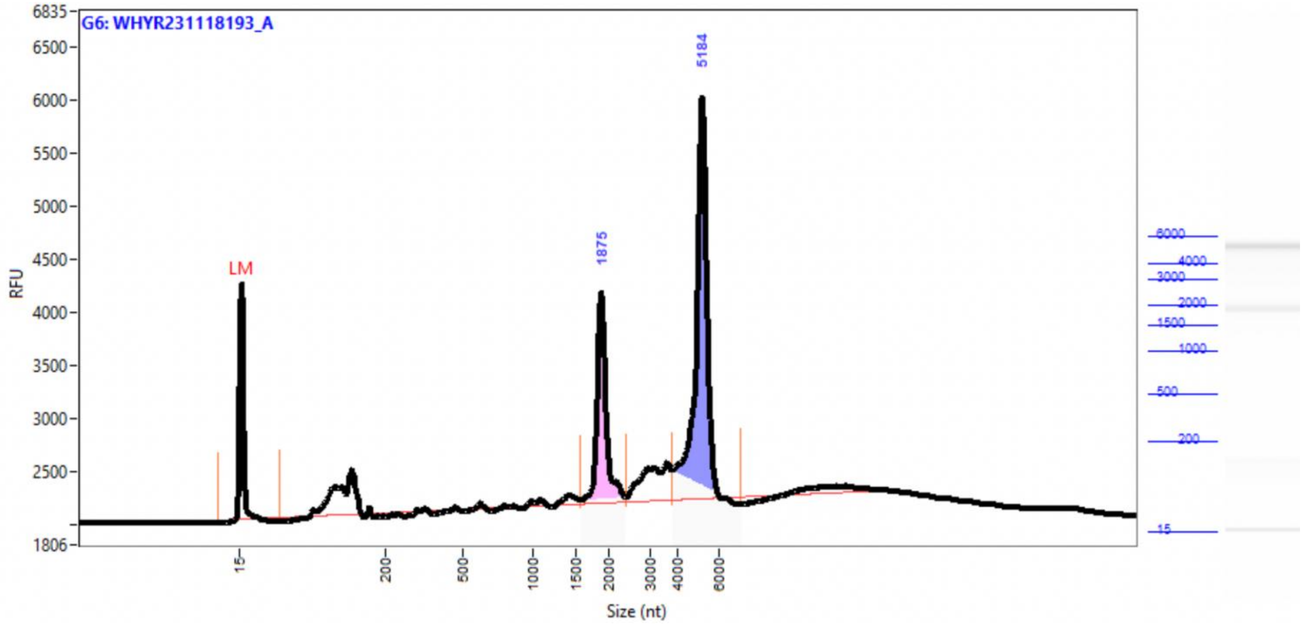

48. Sample Name: LV231026-013A

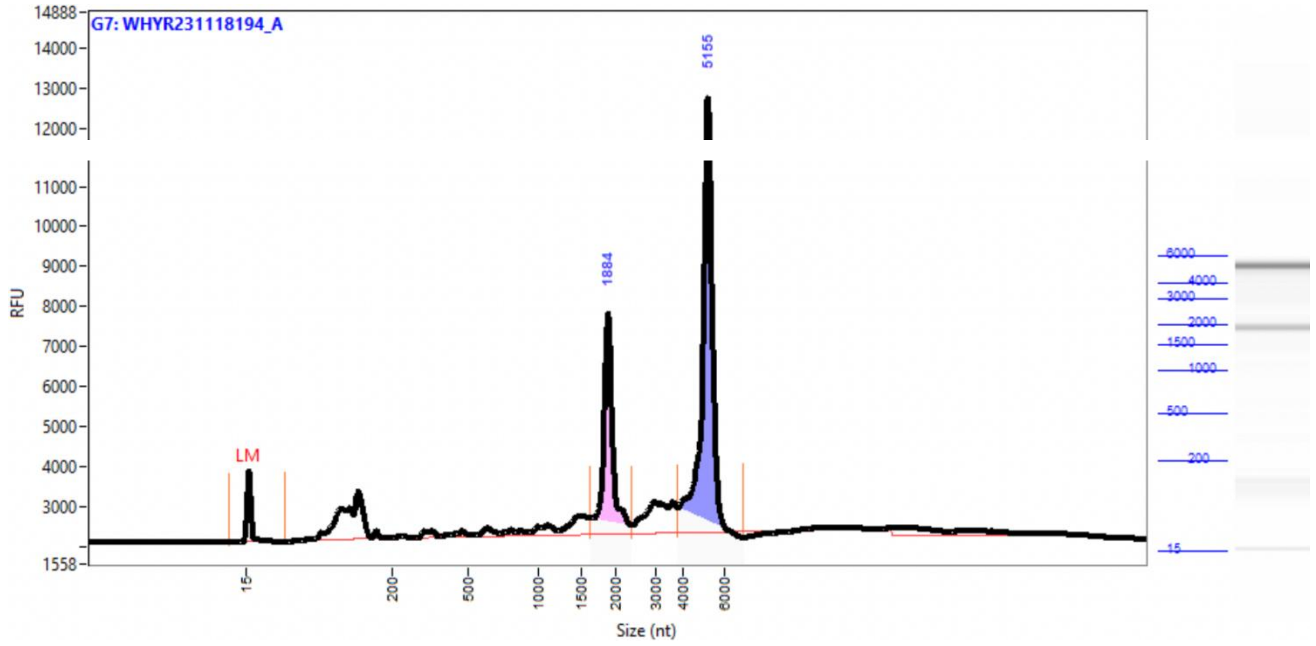

49. Sample Name: LV230905-016A

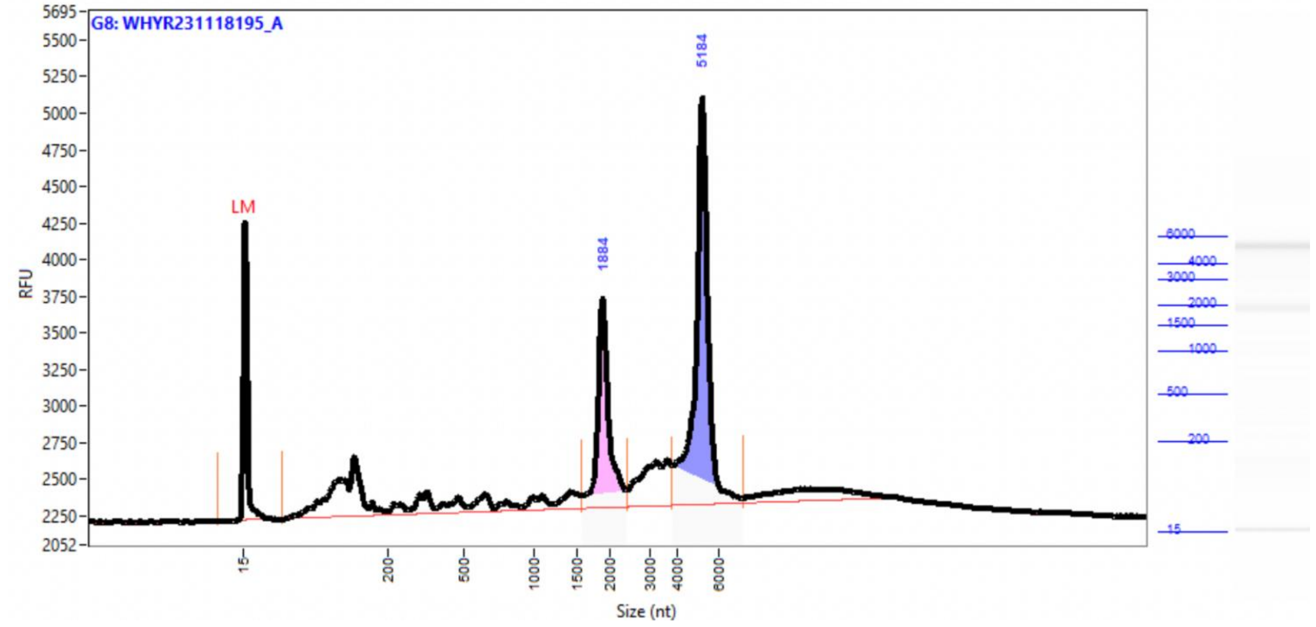

50. Sample Name: LV230922-002A

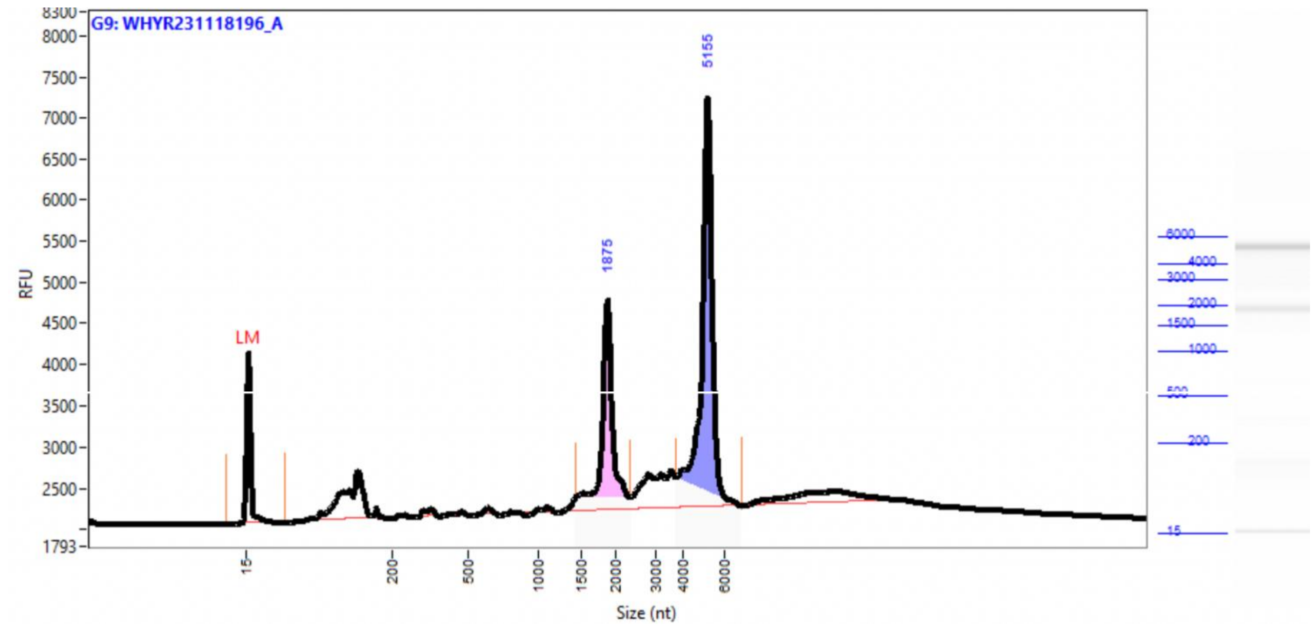

51. Sample Name: LV230904-005A

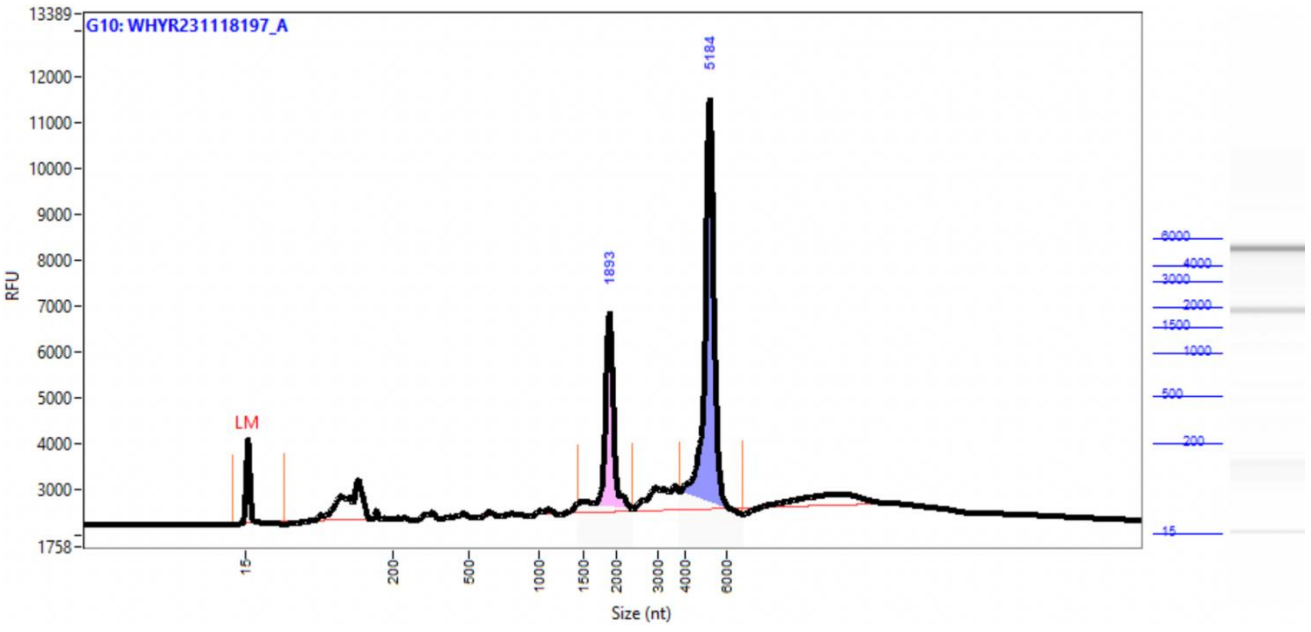

52. Sample Name: LV231023-018A

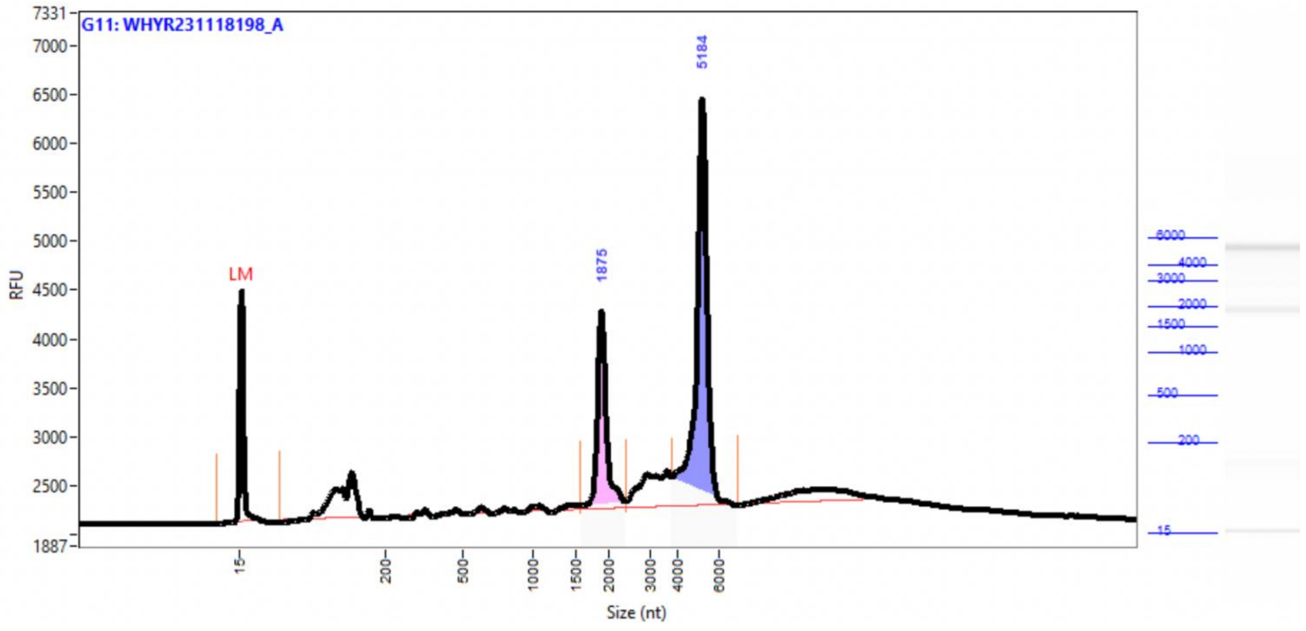

53. Sample Name: LV231024-013A

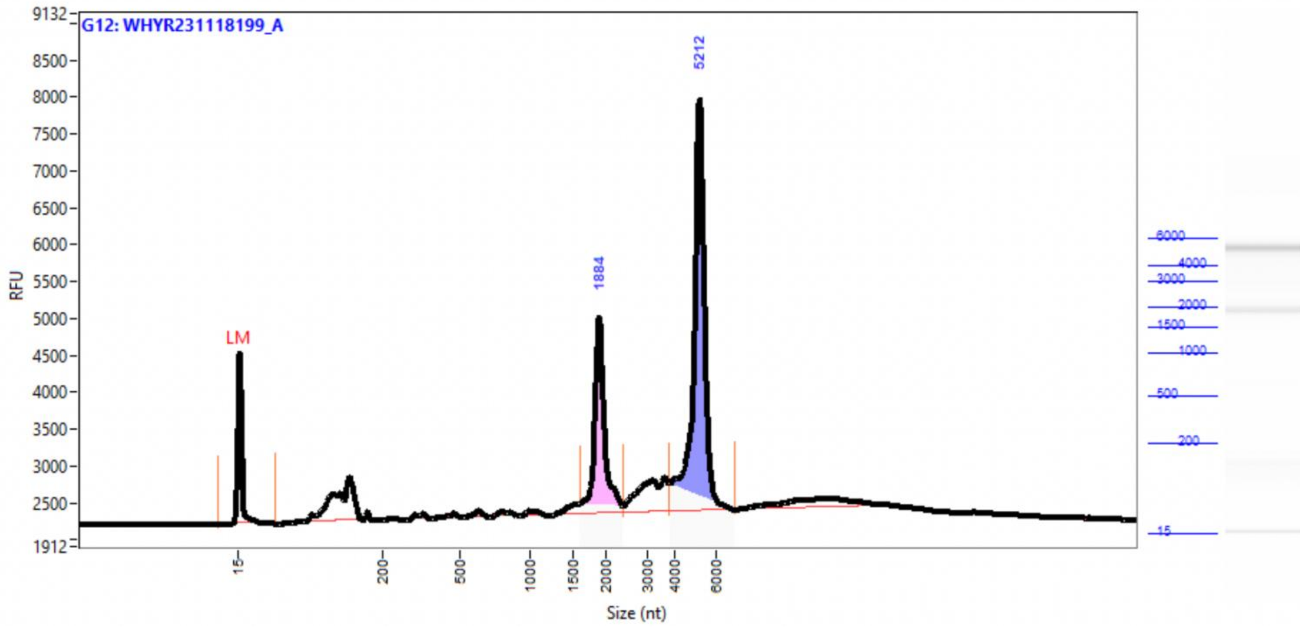

54. Sample Name: LV231023-019A

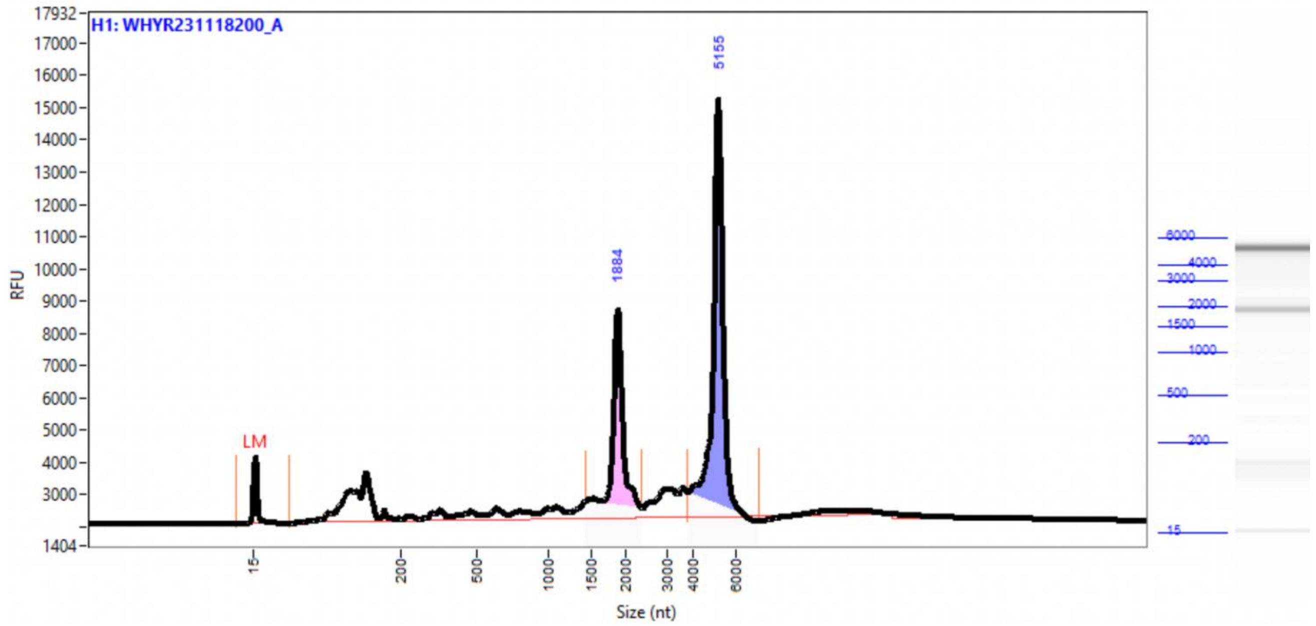

Sample Name	Detection Instrument	Detection Kit	Dilution Factor (x)	Detection Concentration (ng/μL)	RIN/RQN	28S/18S	Remarks
TT230411-001A	Fragment Analyzer	Standard Sensitivity RNA Analysis Kit(15nt) (DNF-471)	1	40	1.1	0.0	
TT230411-002A	Fragment Analyzer	Standard Sensitivity RNA Analysis Kit(15nt) (DNF-471)	1	54	1.3	0.0	
TT230411-003A	Fragment Analyzer	Standard Sensitivity RNA Analysis Kit(15nt) (DNF-471)	1	181	8.6	1.9	
TT230411-004A	Fragment Analyzer	Standard Sensitivity RNA Analysis Kit(15nt) (DNF-471)	5	39	7.8	1.8	
TT230411-006A	Fragment Analyzer	Standard Sensitivity RNA Analysis Kit(15nt) (DNF-471)	1	145	7.2	1.9	
LV230413-003A	Fragment Analyzer	Standard Sensitivity RNA Analysis Kit(15nt) (DNF-471)	5	74	6.2	1.4	
LV230413-004A	Fragment Analyzer	Standard Sensitivity RNA Analysis Kit(15nt) (DNF-471)	1	192	6.8	1.6	
LV230413-005A	Fragment Analyzer	Standard Sensitivity RNA Analysis Kit(15nt) (DNF-471)	1	127	7.0	1.8	
LV230413-006A	Fragment Analyzer	Standard Sensitivity RNA Analysis Kit(15nt) (DNF-471)	1	105	6.5	1.5	
LV230414-006A	Fragment Analyzer	Standard Sensitivity RNA Analysis Kit(15nt) (DNF-471)	1	182	6.5	2.1	
LV230417-009A	Fragment Analyzer	Standard Sensitivity RNA Analysis Kit(15nt) (DNF-471)	5	60	7.6	2.8	
LV230601-002A	Fagment Analyzer	Standard Sensitivity RNA Analysis Kit(15nt) (DNF-471)	1	164	7.7	2.4	
LV230825-009A	Fragment Analyzer	Standard Sensitivity RNA Analysis Kit(15nt) (DNF-471)	5	56	8.8	2.3	
LV230826-016A	Fragment Analyzer	Standard Sensitivity RNA Analysis Kit(15nt) (DNF-471)	5	44	8.6	2.5	
LV230829-014A	Fagment Analyzer	Standard Sensitivity RNA Analysis Kit(15nt) (DNF-471)	5	69	6.5	2.0	
LV230829-015A	Fragment Analyzer	Standard Sensitivity RNA Analysis Kit(15nt) (DNF-471)	5	82	6.2	2.1	
LV230829-016A	Fragment Analyzer	Standard Sensitivity RNA Analysis Kit(15nt) (DNF-471)	5	46	7.9	1.5	
LV230830-015A	Fragment Analyzer	Standard Sensitivity RNA Analysis Kit(15nt) (DNF-471)	1	160	8.2	2.1	
LV230831-014A	Fragment Analyzer	Standard Sensitivity RNA Analysis Kit(15nt) (DNF-471)	5	70	8.6	1.9	
LV230904-003A	Fragment Analyzer	Standard Sensitivity RNA Analysis Kit(15nt) (DNF-471)	1	158	8.2	1.7	
LV230912-010A	Fragment Analyzer	Standard Sensitivity RNA Analysis Kit(15nt) (DNF-471)	5	50	8.9	1.9	
LV230927-008A	Fragment Analyzer	Standard Sensitivity RNA Analysis Kit(15nt) (DNF-471)	1	208	8.2	1.8	
LV231020-019A	Fragment Analyzer	Standard Sensitivity RNA Analysis Kit(15nt) (DNF-471)	5	113	9.9	2.0	
LV231023-020A	Fragment Analyzer	Standard Sensitivity RNA Analysis Kit(15nt) (DNF-471)	5	89	9.4	1.8	
TT230411-007A	Fragment Analyzer	Standard Sensitivity RNA Analysis Kit(15nt) (DNF-471)	1	212	7.9	1.7	
TT230411-008A	Fragment Analyzer	Standard Sensitivity RNA Analysis Kit(15nt) (DNF-471)	1	172	6.7	1.3	
TT230411-009A	Fragment Analyzer	Standard Sensitivity RNA Analysis Kit(15nt) (DNF-471)	1	195	8.3	1.6	
TT230411-010A	Fragment Analyzer	Standard Sensitivity RNA Analysis Kit(15nt) (DNF-471)	1	198	7.0	2.0	
TT230411-011A	Fragment Analyzer	Standard Sensitivity RNA Analysis Kit(15nt) (DNF-471)	5	94	6.9	1.2	
TT230411-012A	Fragment Analyzer	Standard Sensitivity RNA Analysis Kit(15nt) (DNF-471)	1	159	8.9	1.8	
LV230412-004A	Fragment Analyzer	Standard Sensitivity RNA Analysis Kit(15nt) (DNF-471)	1	188	6.8	1.6	
LV230421-008A	Fragment Analyzer	Standard Sensitivity RNA Analysis Kit(15nt) (DNF-471)	5	65	9.1	1.3	
LV230513-012A	Fragment Analyzer	Standard Sensitivity RNA Analysis Kit(15nt) (DNF-471)	1	198	7.3	1.4	
LV230513-013A	Fragment Analyzer	Standard Sensitivity RNA Analysis Kit(15nt) (DNF-471)	5	38	9.6	2.2	
LV230513-014A	Fragment Analyzer	Standard Sensitivity RNA Analysis Kit(15nt) (DNF-471)	1	171	9.1	2.0	
LV230602-004A	Fragment Analyzer	Standard Sensitivity RNA Analysis Kit(15nt) (DNF-471)	5	77	9.1	1.5	
LV230904-004A	Fragment Analyzer	Standard Sensitivity RNA Analysis Kit(15nt) (DNF-471)	5	64	9.9	1.7	
LV230906-001A	Fragment Analyzer	Standard Sensitivity RNA Analysis Kit(15nt) (DNF-471)	1	173	7.7	1.7	
LV230906-002A	Fragment Analyzer	Standard Sensitivity RNA Analysis Kit(15nt) (DNF-471)	1	229	8.2	1.9	
LV230907-008A	Fragment Analyzer	Standard Sensitivity RNA Analysis Kit(15nt) (DNF-471)	1	149	8.9	2.0	
LV230907-009A	Fragment Analyzer	Standard Sensitivity RNA Analysis Kit(15nt) (DNF-471)	1	183	8.8	2.1	
LV230907-010A	Fragment Analyzer	Standard Sensitivity RNA Analysis Kit(15nt) (DNF-471)	1	100	9.3	2.1	
LV230907-011A	Fragment Analyzer	Standard Sensitivity RNA Analysis Kit(15nt) (DNF-471)	1	161	9.0	2.2	
LV230908-011A	Fragment Analyzer	Standard Sensitivity RNA Analysis Kit(15nt) (DNF-471)	1	227	7.7	1.8	
LV230908-012A	Fragment Analyzer	Standard Sensitivity RNA Analysis Kit(15nt) (DNF-471)	1	191	8.4	2.0	
LV230912-009A	Fragment Analyzer	Standard Sensitivity RNA Analysis Kit(15nt) (DNF-471)	5	58	8.7	1.8	
LV231018-006A	Fragment Analyzer	Standard Sensitivity RNA Analysis Kit(15nt) (DNF-471)	5	42	10.0	2.0	

LV231026-013A	Fragment Analyzer	Standard Sensitivity RNA Analysis Kit(15nt)(DNF-471)	1	161	9.5	2.1	
LV230905-016A	Fragment Analyzer	Standard Sensitivity RNA Analysis Kit(15nt)(DNF-471)	5	47	8.9	1.9	
LV230922-002A	Fragment Analyzer	Standard Sensitivity RNA Analysis Kit(15nt)(DNF-471)	5	64	10.0	2.0	
LV230904-005A	Fragment Analyzer	Standard Sensitivity RNA Analysis Kit(15nt)(DNF-471)	1	113	10.0	2.2	
LV231023-018A	Fragment Analyzer	Standard Sensitivity RNA Analysis Kit(15nt)(DNF-471)	5	44	10.0	2.1	
LV231024-013A	Fragment Analyzer	Standard Sensitivity RNA Analysis Kit(15nt)(DNF-471)	5	59	10.0	2.0	
LV231023-019A	Fragment Analyzer	Standard Sensitivity RNA Analysis Kit(15nt)(DNF-471)	1	179	9.3	2.0	

Attachment 2: Original Sample Information

Sample Type:

Sample Status:

Sample Details:

Sample Name	Species	Number of Tubes	Total Sample Amount	Collection Time	Whether Multi-tube Samples with the Same Name are Extracted Together	Remarks
LV230414-006	Human blood	1	0			
LV230413-006	Human blood	1	0			
LV230413-005	Human blood	1	0			
LV230413-004	Human blood	1	0			
LV231023-019	Human blood	1	0			
LV231024-013	Human blood	1	0			
LV231023-018	Human blood	1	0			
LV230904-005	Human blood	1	0			
LV230413-003	Human blood	1	0			
LV230922-002	Human blood	1	0			
LV230905-016	Human blood	1	0			
LV231026-013	Human blood	1	0			
LV231018-006	Human blood	1	0			
LV230912-009	Human blood	1	0			
LV230908-012	Human blood	1	0			
LV230908-011	Human blood	1	0			
LV230907-011	Human blood	1	0			
LV230907-010	Human blood	1	0			
LV230907-009	Human blood	1	0			
TT230411-006	Human blood	1	0			
LV230907-008	Human blood	1	0			
LV230906-002	Human blood	1	0			
LV230906-001	Human blood	1	0			
LV230904-004	Human blood	1	0			
LV230602-004	Human blood	1	0			
LV230513-014	Human blood	1	0			
LV230513-013	Human blood	1	0			
LV230513-012	Human blood	1	0			
LV230421-008	Human blood	1	0			
LV230412-004	Human blood	1	0			
TT230411-004	Human blood	1	0			
TT230411-012	Human blood	1	0			
TT230411-011	Human blood	1	0			
TT230411-010	Human blood	1	0			

TT230411-009	Human blood	1	0			
TT230411-008	Human blood	1	0			
TT230411-007	Human blood	1	0			
LV231023-020	Human blood	1	0			
LV231020-019	Human blood	1	0			
LV230927-008	Human blood	1	0			
LV230912-010	Human blood	1	0			
TT230411-003	Human blood	1	0			
LV230904-003	Human blood	1	0			
LV230831-014	Human blood	1	0			
LV230830-015	Human blood	1	0			
LV230829-016	Human blood	1	0			
LV230829-015	Human blood	1	0			
LV230829-014	Human blood	1	0			
LV230826-016	Human blood	1	0			
LV230825-009	Human blood	1	0			
LV230601-002	Human blood	1	0			
LV230417-009	Human blood	1	0			
TT230411-002	Human blood	1	0			
TT230411-001	Human blood	1	0			

——— Report ends ———

Quality control information of traditional Chinese medicine components contained in QXTTF (Attached with quality inspection report 1- 9)

Serial Number	Traditional Chinese Medicine components contained in QXTTF	Chinese Name	Type	Purchase Date	Purchased phamaceutical factory	Place of origin of medicinal materials	Batch Number	Quality control
1	Lonicerae japonicae Flos	Jinyinhua	Traditional Chinese Medicine Decoction Pieces	Jul. 2023	Bozhou Yonggang Traditional Chinese Medicine Co., Ltd.	Henan, China	A230324	Report 1
2	Phragmitis Rhizoma	Lugen	Traditional Chinese Medicine Decoction Pieces	Jul. 2023	Guangdong Medicinal Materials Company Chinese Herbal Pieces Factory	Hebei, China	L1422922	Report 2
3	Imperatae Rhizoma	Baimaogen	Traditional Chinese Medicine Decoction Pieces	Jul. 2023	Bozhou Yonggang Traditional Chinese Medicine Co., Ltd.	Hebei, China	A230624	Report 3
4	Pogostemonis Herba	Huoxiang	Traditional Chinese Medicine Decoction Pieces	Jul. 2023	Guangdong Medicinal Materials Company Chinese Herbal Pieces Factory	Guangdong, China	G2172211	Report 4
5	Angelicae dahuricae Radix	Baizhi	Traditional Chinese Medicine Decoction Pieces	Jul. 2023	Bozhou Yonggang Traditional Chinese Medicine Co., Ltd.	Anhui, China	A230102	Report 5
6	Citri Reticulatae Pericarpium	Chenpi	Traditional Chinese Medicine Decoction Pieces	Jul. 2023	Guangdong Huidakang Pharmaceutical Co.,Ltd.	Gungdong, China	230601	Report 6
7	Ginseng Radix et Rhizoma	Renshen	Traditional Chinese Medicine Decoction Pieces	Jul. 2023	Guangdong Huidakang Pharmaceutical Co.,Ltd.	Jilin, China	230801	Report 7
8	Dioscoreae Rhizoma	Shanyao	Traditional Chinese Medicine Decoction Pieces	Jul. 2023	Bozhou Yonggang Traditional Chinese Medicine Co., Ltd.	Henan, China	A220814	Report 8
9	Glycyrrhizae Radix et Rhizoma	Gancao	Traditional Chinese Medicine Decoction Pieces	Jul. 2023	Bozhou Yonggang Traditional Chinese Medicine Co., Ltd.	Inner Mongolia, China	A220623	Report 9

Report 1: Lonicerae japonicae Flos (Chinese: Jinyinhua)

Finished Product Inspection Report

Record Number: QC-SMP(JY)003-R03-01

**Product Name**	Lonicerae japonicae Flos (Chinese: Jinyinhua)	**Inspection Number**	CP0432003-A230324
**Batch Number**	A230324	**Inspection Type**	factory inspection
**Specification**	Selected	**Application Date**	2023-03-25
**Sample Quantity**	300g	**Report Date**	2023-03-25
**Batch Quantity**	1060kg	**Sample Source**	Finished Product Warehouse
**Inspection standard**	Chinese Pharmacopoeia (2020 Edition, Volume I)		

Inspection Item	Standard Specification	Inspection Result
* [Character]	This product is rod-shaped, thick at the upper part and thin at the lower part, slightly curved, 2–3 cm long, with an upper diameter of about 3 mm and a lower diameter of about 1.5 mm. The surface is yellowish-white or greenish-white (the color gradually darkens during storage), densely covered with short soft hairs. Leaf-like bracts are occasionally seen. The calyx is green, 5-lobed at the apex, with hairy lobes, about 2 mm long. The open corolla is tubular, bilabiate at the apex; there are 5 stamens, attached to the tube wall, yellow; 1 pistil, and the ovary is glabrous. It has a faint fragrance, and the taste is light and slightly bitter.	Complies with Specification
* [Identification]		
Microscopic Characteristics	Should have the microscopic characteristics of Lonicerae Japonicae Flos.	Has the microscopic characteristics of Lonicerae Japonicae Flos.
Thin-Layer Chromatography	In the chromatogram of the test sample, fluorescent spots of the same color should appear at the positions corresponding to those in the chromatogram of the reference substance.	Shows fluorescent spots of the same color.
* [Inspection]		
Moisture	Not more than 12.0%	7.3%
Total Ash	Not more than 10.0%	5.6%
Acid-Insoluble Ash	Not more than 3.0%	0.4%
Heavy Metals and Harmful Elements		
-Lead (Pb)	Not more than 5mg/kg	1mg/kg
- Cadmium (Cd)	Not more than 1mg/kg	0.1mg/kg
- Arsenic (As)	Not more than 2mg/kg	0.4mg/kg
- Mercury (Hg)	Not more than 0.2mg/kg	0.001mg/kg
- Copper (Cu)	Not more than 20mg/kg	12mg/kg
Residual Sulfur Dioxide	Not more than 150mg/kg	0mg/kg
* [Characteristic Chromatogram]	In the characteristic chromatogram of the test sample, 7 characteristic peaks should appear. The peak corresponding to the reference substance peak is peak S. Calculate the relative retention time of each characteristic peak and peak S, which should be within ±10% of the specified value.	Complies with Specification

Note: Items marked with * are referenced test data.

Report 2: Phragmitis Rhizoma (Chinese: Lugen) Finished Product Inspection Report

Report Number: C221214L14-1

**Sample Name**	Phragmitis Rhizoma (Chinese: Lugen)	**Origin**	Hebei, China
**Specification**	20kg (Bulk Packaging)	**Subpackage Spec.**	3g, 5g, 10g, 15g, 20g, 0.5kg, 1kg, standard
**Batch Number**	L1422922	**Quantity**	5Kg
**Sample Source**	Finished Product Warehouse (Sampling Inspection)	**Production Date**	December 09, 2022
**Sampling Quantity**	200g	**Sampling Date**	December 14, 2022
**Sampler**	Peng Jiaqu	**Receipt Date**	December 14, 2022
**Inspection Purpose**	Quality Inspection	**Issuance Date**	December 16, 2022
**Inspection Basis**	Chinese Pharmacopoeia (2020 Edition), Internal Quality Standard for Rhizoma Phragmitis Decoction Pieces		

Inspection Item	Standard Specification	Inspection Result
**[Character]**	Should comply with the character regulations under Phragmitis Rhizoma.	Complies with the character regulations under Phragmitis Rhizoma.
**[Identification]**	Should comply with the identification regulations under Phragmitis Rhizoma.	
	Microscopic Identification	Complies with Specification
	Thin-Layer Chromatography Identification	Complies with Specification
**[Inspection]**		
Impurity	Not more than 3%	0.1%
Moisture	Not more than 12.0%	8.3%
Total Ash	Not more than 11.0%	5.3%
Acid-Insoluble Ash	Not more than 8.0%	1.7%
Residual Sulfur Dioxide	Not more than 150mg/kg	9mg/kg
**[Extract]**	Water-soluble extract not less than 12.0%	20.0%

Remarks: The test results of total ash, acid-insoluble ash, and sulfur dioxide under [Identification] and [Inspection] refer to the test data of the original medicinal materials of this batch.

Conclsion	This product is tested in accordance with the Chinese Pharmacopoeia (2020 Edition) and the internal quality standard for Phragmitis Rhizoma decoction pieces, and the results comply with the specifications.				
Inspector	Liang Jianwei	Reviewer	Dong Xuelian	Person in Charge	Chen Yuedong

Report 3: Imperatae Rhizoma (Chinese: Baimaogen)

Finished Product Inspection Report

Record Number: QC-SMP(JY)003-R03-01

**Product Name**	Imperatae Rhizoma (Chinese: Baimaogen)	**Inspection Number**	N20230628140041
**Batch Number**	A230624	**Inspection Type**	factory inspection
**Specification**	Selected	**Application Date**	2023-06-28
**Sample Quantity**	300g	**Report Date**	2023-06-29
**Batch Quantity**	1650kg	**Sample Source**	Finished Product
**Inspection standard**	Chinese Pharmacopoeia (2020 Edition, Volume I)		

Inspection Item	Standard Specification	Inspection Result
**[Character]**	This product is in cylindrical segments. The outer epidermis is yellowish-white or pale yellow, slightly glossy, with longitudinal wrinkles, and some show slightly raised nodes. The cut surface of the cortex is white, mostly with fissures arranged radially, and the stele is pale yellow or hollow, easily separable from the cortex. It has a faint odor and a slightly sweet taste.	Complies with Specification
*** [Identification]**		
Microscopic Characteristics	Should have the microscopic characteristics of Imperatae Rhizoma.	Complies with Specification
Thin-Layer Chromatography	In the chromatogram of the test sample, spots of the same color should appear at the positions corresponding to those in the chromatogram of the reference medicinal material.	Complies with Specification
**[Inspection]**		
Moisture	Not more than 12.0%	9.2%
Total Ash	Not more than 5.0%	2.9%
*Residual Sulfur Dioxide	Not more than 150mg/kg	0mg/kg
**[Extract]**	Not less than 28.0% calculated on the dried basis	42.5%

Note: Items marked with * are referenced test data.

**Conclusion**	This product was tested according to the 2020 edition of the China Pharmacopoeia, Part I, and the results were in accordance with the regulations.		
**Reviewer**	Li Wei	**Approver**	Huo Mingxing

Report 4: Pogostemonis Herba (Chinese: Huoxiang)
Finished Product Inspection Report

Report Number: C221218G21-1

**Sample Name**	Pogostemonis Herba (Chinese: Huoxiang)	**Origin**	Guangdong, China
**Specification**	20kg (Bulk Packaging)	**Subpackage Spec.**	3g,5g,10g,15g,20g,0.5kg, 1kg, standard
**Batch Number**	G2172211	**Quantity**	5Kg
**Sample Source**	Finished Product Warehouse (Sampling Inspection)	**Production Date**	December 16,2022
**Sampling Quantity**	200g	**Sampling Date**	December 18, 2022
**Sampler**	Peng Jiaqu	**Receipt Date**	December 18, 2022
**Inspection Purpose**	Quality Inspection	**Issuance Date**	December 18, 2022
**Inspection Basis**	Chinese Pharmacopoeia (2020 Edition), Internal Quality Standard for pogostemonis Herb Decoction Pieces		

Inspection Item	Standard Specification	Inspection Result
**[Character]**	Should comply with the character regulations under Pogostemonis Herba.	Complies with the character regulations under Pogostemonis Herba.
**[Identification]**	Should comply with the identification regulations under Pogostemonis Herba.	
	Microscopic Identification	Complies with Specification
	Thin-Layer Chromatography Identification	Complies with Specification
**[Inspection]**		
Impurity	Not more than 3%	0.1%
Moisture	Not more than 14.0%	10.0%
Residual Sulfur Dioxide	Not more than 150mg/kg	7mg/kg

Remarks: The test results of sulfur dioxide under the item of [Identification] [Check] refer to the test data of the original batch of medicinal materials.

Conclusion	This product was tested according to the 2020 edition of the China Pharmacopoeia and the internal quality control standard of Huoxiang (Pogostemonis Herba) decoction pieces, and the results were in accordance with the regulations.				
Inspector	Liang Jianwei	Reviewer	Dong Xuelian	Person in Charge	Chen Yuedong

Report 5: Angelicae dahuricae Radix (Chinese: Baizhi)

Finished Product Inspection Report

Record Number: QC-SMP(JY)003-R03-01

**Product Name**	Angelicae dahuricae Radix (Chinese: Baizhi)	**Inspection Number**	CP0052002-A230102
**Batch Number**	A230102	**Inspection Type**	factory inspection
**Specification**	Selected	**Application Date**	2023-01-06
**Sample Quantity**	400g	**Report Date**	2023-01-06
**Batch Quantity**	1500kg	**Sample Source**	Finished Product Warehouse
**Inspection standard**	Chinese Pharmacopoeia (2020 Edition, Volume I)		

Inspection Item	Standard Specification	Inspection Result
**[Character]**	This product is in thick subrounded slices. The outer epidermis is grayish-brown or yellowish-brown. The cut surface is white or grayish-white, starchy, with a brown cambium ring, subquadrate or subrounded, and the cortex scattered with numerous brown oil spots. It has an aromatic odor, and a pungent, slightly bitter taste.	Complies with Specification
*** [Identification]**		
Microscopic Characteristics	Should have the microscopic characteristics of Baizhi (Angelicae dahuricae Radix).	Has the microscopic characteristics of Baizhi (Angelicae dahuricae Radix)
Thin-Layer Chromatography	In the chromatogram of the test sample, fluorescent spots of the same color should appear at the positions corresponding to those in the chromatograms of the reference medicinal material and reference substance.	Fluorescent spots of the same color appear
**[Inspection]**		
Moisture	Not more than 14.0%	8.8%
Total Ash	Not more than 5.0%	3.0%
*Residual Sulfur Dioxide	Not more than 150mg/kg	0mg/kg
*** [Extract]**	Not less than 28.0% calculated on the dried basis	18.2%
*** [Assay]**	Calculated on the dried basis, contains imperatorin ($C_{16}H_{14}O_4$) not less than 0.080%.	0.181%

Note: Items marked with * are referenced test data.

**Conclusion**	This product was tested according to the 2020 edition of the China Pharmacopoeia, Part I, and the results were in accordance with the regulations.		
**Reviewer**	Liu Jiayi	**Approver**	Huo Mingxing

Report 6: Citri Reticulatae Pericarpium (Chinese: Chenpi)
Finished Product Inspection Report

QR/SMP-09-12-03

Inspection number: IM23061602

**Product Name**	Citri Reticulatae Pericarpium (Chinese: Chenpi)	**Product Code**	UM2-022A
**Batch Number**	230601	**Quantity**	3416.60kg
**Production Date**	June 2, 2023	**Origin**	Guangxi, China
**Specification**	Silk	**Report date**	June 20,2023
**Inspection Standard**	Chinese Pharmacopoeia (2020 Edition, Volume I)		

Inspection Item	Standard Specification	Inspection Result
**[Character]**	Should have the character characteristics of Chenpi (Citri Reticulatae Pericarpium).	Has the character characteristics of Chenpi (Citri Reticulatae Pericarpium).
**[Identification]**		
Microscopic Characteristics	Should have the microscopic characteristics of Chenpi (Citri Reticulatae Pericarpium).	Has the microscopic characteristics of Chenpi (Citri Reticulatae Pericarpium).
Thin-Layer Chromatography	In the chromatogram of the test sample, fluorescent spots of the same color should appear at the positions corresponding to those in the chromatogram of the reference substance.	In the chromatogram of the test sample, fluorescent spots of the same color appear at the positions corresponding to those in the chromatogram of the reference substance.
**[Inspection]**		
Medicinal Debris and Impurities	Not more than 2.0%	0.2%
Moisture	Not more than 13.0%	7.1%
Aflatoxins	Aflatoxin B_1 not more than 5μg/kg; Total of aflatoxin G_2, G_1, B_2 and B_1 not more than 10μg/kg	Not detected; Not detected
Residual Sulfur Dioxide	Not more than 150mg/kg	Not detected
**[Assay]**	Not less than 2.5%	6.2%

**Conclusion**	This product is tested in accordance with the Chinese Pharmacopoeia (2020 Edition, Volume I and IV) and the internal quality standard for Chenpi (Citri Reticulatae Pericarpium) decoction pieces, and the results comply with the specifications.				
**Inspector**	Zhang Xiaofen	**Reviewer**	He Linsheng	**Person in Charge**	Li Haimei

Report 7: Ginseng Radix et Rhizoma (Chinese: Renshen) Finished Product Inspection Report

QR/SMP-09-12-03

Inspection number: IM 23090503

**Product Name**	Ginseng Radix et Rhizoma (Chinese: Renshen)	**Product Code**	UM1-070B
**Batch Number**	230801	**Quantity**	351.30kg
**Production Date**	August 29,2023	**Origin**	Jilin, China
**Specification**	Slide	**Report date**	September 11,2023
**Inspection Standard**	Chinese Pharmacopoeia (2020 Edition, Volume I)		

Inspection Item	Standard Specification	Inspection Result
**[Character]**	Should have the character characteristics of Renshen (Ginseng Radix et Rhizoma).	Has the character characteristics of Renshen (Ginseng Radix et Rhizoma).
**[Identification]**		
Microscopic Characteristics	Should have the microscopic characteristics of Renshen (Ginseng Radix et Rhizoma).	Has the microscopic characteristics of Renshen (Ginseng Radix et Rhizoma).
Thin-Layer Chromatography	In the chromatogram of the test sample, spots or fluorescent spots of the same color should appear at the positions corresponding to those in the chromatograms of the reference medicinal material and reference substance.	In the chromatogram of the test sample, spots or fluorescent spots of the same color appear at the positions corresponding to those in the chromatograms of the reference medicinal material and reference substance.
**[Inspection]**		
Medicinal Debris and Impurities	Not more than 3.0%	0.4%
Moisture	Not more than 12.0%	10.7%
Total Ash	Not more than 5.0%	3.2%
Heavy Metals and Harmful Elements	Pb ≤5mg/kg;<br>Cd ≤1mg/kg;<br>As ≤2mg/kg;<br>Hg ≤0.2mg/kg;<br>Cu ≤20mg/kg	Pb: 0.08mg/kg;<br>Cd: 0.04mg/kg;<br>As: 0.01mg/kg;<br>Hg: 0.09mg/kg;<br>Cu: 8.1mg/kg
Residual Sulfur Dioxide	Not more than 150mg/kg	Not detected
Organochlorine Pesticide Residues	Quintozene ≤0.1mg/kg; Hexachlorobenzene ≤0.1mg/kg; Heptachlor (sum of heptachlor and heptachlor epoxide) ≤0.05mg/kg; Chlordane (sum of cis-chlordane, trans-chlordane, and oxychlordane) ≤0.1mg/kg	Not detected; Not detected; Not detected; Not detected
**[Assay]**		
Total of Ginsenoside Rg1 and Ginsenoside Re	Not less than 0.27%	0.36%
Ginsenoside Rb1	Not less than 0.18%	0.23%

**Conclusion**	This product is tested in accordance with the Chinese Pharmacopoeia (2020 Edition, Volume I and IV) and the internal quality standard for Renshen (Ginseng Radix et Rhizoma) decoction pieces, and the results comply with the specifications.				
**Inspector**	Zhang Xiaofen	**Reviewer**	He Linsheng	**Person in Charge**	Li Haimei

Report 8: Dioscoreae Rhizoma (Chinese: Shanyao)

Finished Product Inspection Report

Record Number: QC-SMP (JY) 003-R03-01

**Product Name**	Dioscoreae Rhizoma (Chinese: Shanyao)	**Inspection Number**	CP0698007-A220814
**Batch Number**	A220814	**Inspection Type**	factory inspection
**Specification**	Selected	**Application Date**	2022-08-17
**Sample Quantity**	400g	**Report Date**	2022-08-18
**Batch Quantity**	2000kg	**Sample Source**	Finished Product Warehouse
**Inspection standard**	Chinese Pharmacopoeia (2020 Edition, Volume I)		

Inspection Item	Standard Specification	Inspection Result
* [Character]	This product is in subrounded, elliptical or irregular thick slices. The surface is off-white or pale yellowish-white, brittle, easily broken, and the cut surface is off-white, starchy. It has a faint odor, a bland and slightly acidic taste, and is sticky when chewed.	Complies with Specification
* [Identification]		
Microscopic Characteristics	Should have the microscopic characteristics of Dioscoreae Rhizoma.	Has the microscopic characteristics of Dioscoreae Rhizoma
Thin-Layer Chromatography	In the chromatogram of the test sample, fluorescent spots of the same color should appear at the positions corresponding to those in the chromatogram of the reference medicinal material.	Fluorescent spots of the same color appear
* [Inspection]		
Moisture	Not more than 16.0%	13.4%
Total Ash	Not more than 4.0%	1.6%
Residual Sulfur Dioxide	Not more than 400mg/kg	156mg/kg
* [Extract]	Not less than 4.0% calculated on the dried basis.	5.2%

Note: Items marked with * are referenced test data.

**Conclusion**	This product is tested in accordance with the Chinese Pharmacopoeia (2020 Edition, Volume I), and the results comply with the specifications.		
**Reviewer**	Shen Minghui	**Approver**	Wang Yonglei

Report 9: Glycyrrhizae Radix et Rhizoma (Chinese: Gancao)

Finished Product Inspection Report

Record Number: QC-SMP (JY) 003-R03-01

**Product Name**	Glycyrrhizae Radix et Rhizoma (Chinese: Gancao)	**Inspection Number**	DBH0600477-A220623
**Batch Number**	A220623	**Inspection Type**	factory inspection
**Specification**	Selected	**Application Date**	2022-06-26
**Sample Quantity**	400g	**Report Date**	2022-06-26
**Batch Quantity**	1500kg	**Sample Source**	Finished Product Warehouse
**Inspection standard**	Chinese Pharmacopoeia (2020 Edition, Volume I)		

Inspection Item	Standard Specification	Inspection Result
* [Character]	This product is in the form of subcircular or elliptical thick slices. The outer skin is reddish brown or grayish brown, with longitudinal wrinkles. The cut surface is slightly fibrous, the center is yellowish white, with obvious radial texture and cambium ring. It is solid and powdery. Slight odor, sweet and peculiar taste.	Complies with Specification
* [Identification]		
Microscopic Characteristics	Should possess the microscopic characteristics of Glycyrrhizae Radix et Rhizom.	Possesses the microscopic characteristics of Glycyrrhizae Radix et Rhizom.
Thin-Layer Chromatography	In the chromatogram of the test sample, at the positions corresponding to the chromatogram of the reference drug, fluorescent spots of the same color should be shown; at the positions corresponding to the chromatogram of the reference substance, orange-yellow fluorescent spots of the same color should be shown.	Shows fluorescent spots of the same color; shows orange -yellow fluorescent spots of the same color.
* [Inspection]		
Moisture	Not more than 12.0%	7.6%
Total Ash	Not more than 5.0%	4.6%
Heavy Metals and Harmful Elements	Lead: Not more than 5mg/kg<br>Cadmium: Not more than 1mg/kg<br>Arsenic: Not more than 2mg/kg<br>Mercury: Not more than 0.2mg/kg<br>Copper: Not more than 20mg/kg	Lead: 1 mg/kg<br>Cadmium: 0.03 mg/kg<br>Arsenic: 0.2 mg/kg<br>Mercury: 0.0004 mg/kg<br>Copper: 8 mg/kg
Sulfur Dioxide Residue	Not more than 150 mg/kg	0 mg/kg
* [Assay]	Calculated on the dried basis, contains liquiritin ($C_{21}H_{22}O_9$) not less than 0.45%, and glycyrrhizic acid ($C_{42}H_{62}O_{16}$) not less than 1.8%.	Liquiritin: 0.54 %<br>Glycyrrhizic acid: 2.6 %

Note: Items marked with * are referenced test data.

**Conclusion**	This product is tested in accordance with the Chinese Pharmacopoeia (2020 Edition, Volume I), and the results comply with the specifications.		
**Reviewer**	Shen Minghui	**Approver**	Wang Yonglei
