# Supplementary material for: Immunomodulatory effects and mechanisms of Qi-Xu-Tiao-Ti formula in Qi-deficiency constitution: a randomized controlled trial integrated with multi-omics and network pharmacology analysis
Source: Front Immunol. 2025 Oct 31;16:1675502. doi: 10.3389/fimmu.2025.1675502 (PMC12615400; doi:10.3389/fimmu.2025.1675502)
Supplement: Supplementary file 2 [file Table1.docx]

**Supplementary Table 1** The list of DEGs1 enriched GO items and KEGG pathways

| ID | Description | GeneRatio | BgRatio | pvalue | p.adjust | qvalue | Count |
| --- | --- | --- | --- | --- | --- | --- | --- |
| hsa04650 | Natural killer cell mediated cytotoxicity | 22/342 | 134/8541 | 0.0000 | 0.0000 | 0.0000 | 22 |
| hsa04613 | Neutrophil extracellular trap formation | 18/342 | 193/8541 | 0.0007 | 0.0007 | 0.0477 | 18 |
| hsa05144 | Malaria | 8/342 | 50/8541 | 0.0007 | 0.0007 | 0.0477 | 8 |
| hsa04662 | B cell receptor signaling pathway | 11/342 | 91/8541 | 0.0010 | 0.0010 | 0.0477 | 11 |
| hsa04810 | Regulation of actin cytoskeleton | 20/342 | 232/8541 | 0.0010 | 0.0010 | 0.0477 | 20 |
| hsa04510 | Focal adhesion | 18/342 | 203/8541 | 0.0013 | 0.0013 | 0.0477 | 18 |
| hsa04612 | Antigen processing and presentation | 10/342 | 81/8541 | 0.0014 | 0.0014 | 0.0477 | 10 |
| hsa05322 | Systemic lupus erythematosus | 14/342 | 141/8541 | 0.0015 | 0.0015 | 0.0477 | 14 |
| hsa04024 | cAMP signaling pathway | 19/342 | 226/8541 | 0.0018 | 0.0018 | 0.0477 | 19 |
| hsa05416 | Viral myocarditis | 9/342 | 70/8541 | 0.0018 | 0.0018 | 0.0477 | 9 |
| hsa05332 | Graft-versus-host disease | 7/342 | 45/8541 | 0.0019 | 0.0019 | 0.0477 | 7 |
| hsa05223 | Non-small cell lung cancer | 9/342 | 73/8541 | 0.0024 | 0.0024 | 0.0555 | 9 |
| hsa05212 | Pancreatic cancer | 9/342 | 77/8541 | 0.0035 | 0.0035 | 0.0741 | 9 |
| hsa04820 | Cytoskeleton in muscle cells | 18/342 | 232/8541 | 0.0054 | 0.0054 | 0.1077 | 18 |
| hsa05412 | Arrhythmogenic right ventricular cardiomyopathy | 9/342 | 86/8541 | 0.0072 | 0.0072 | 0.1112 | 9 |
| hsa05210 | Colorectal cancer | 9/342 | 87/8541 | 0.0078 | 0.0078 | 0.1112 | 9 |
| hsa04530 | Tight junction | 14/342 | 170/8541 | 0.0082 | 0.0082 | 0.1112 | 14 |
| hsa05034 | Alcoholism | 15/342 | 188/8541 | 0.0084 | 0.0084 | 0.1112 | 15 |
| hsa05218 | Melanoma | 8/342 | 73/8541 | 0.0085 | 0.0085 | 0.1112 | 8 |
| hsa00532 | Glycosaminoglycan biosynthesis - chondroitin sulfate / dermatan sulfate | 4/342 | 21/8541 | 0.0088 | 0.0088 | 0.1112 | 4 |
| hsa04923 | Regulation of lipolysis in adipocytes | 7/342 | 59/8541 | 0.0088 | 0.0088 | 0.1112 | 7 |
| hsa05213 | Endometrial cancer | 7/342 | 59/8541 | 0.0088 | 0.0088 | 0.1112 | 7 |
| hsa04062 | Chemokine signaling pathway | 15/342 | 193/8541 | 0.0105 | 0.0105 | 0.1231 | 15 |
| hsa04514 | Cell adhesion molecules | 13/342 | 158/8541 | 0.0107 | 0.0107 | 0.1231 | 13 |
| hsa05170 | Human immunodeficiency virus 1 infection | 16/342 | 213/8541 | 0.0114 | 0.0114 | 0.1231 | 16 |
| hsa05220 | Chronic myeloid leukemia | 8/342 | 77/8541 | 0.0116 | 0.0116 | 0.1231 | 8 |
| hsa05217 | Basal cell carcinoma | 7/342 | 63/8541 | 0.0125 | 0.0125 | 0.1283 | 7 |
| hsa05224 | Breast cancer | 12/342 | 148/8541 | 0.0155 | 0.0155 | 0.1532 | 12 |
| hsa05205 | Proteoglycans in cancer | 15/342 | 204/8541 | 0.0168 | 0.0168 | 0.1577 | 15 |
| hsa05410 | Hypertrophic cardiomyopathy | 9/342 | 99/8541 | 0.0174 | 0.0174 | 0.1577 | 9 |
| hsa04670 | Leukocyte transendothelial migration | 10/342 | 116/8541 | 0.0177 | 0.0177 | 0.1577 | 10 |
| hsa04061 | Viral protein interaction with cytokine and cytokine receptor | 9/342 | 100/8541 | 0.0185 | 0.0185 | 0.1597 | 9 |
| hsa04210 | Apoptosis | 11/342 | 136/8541 | 0.0203 | 0.0203 | 0.1706 | 11 |
| hsa05414 | Dilated cardiomyopathy | 9/342 | 105/8541 | 0.0245 | 0.0245 | 0.1999 | 9 |
| hsa05418 | Fluid shear stress and atherosclerosis | 11/342 | 141/8541 | 0.0258 | 0.0258 | 0.2040 | 11 |
| hsa04380 | Osteoclast differentiation | 11/342 | 143/8541 | 0.0282 | 0.0282 | 0.2087 | 11 |
| hsa03273 | Virion - Lassa virus and SFTS virus | 3/342 | 17/8541 | 0.0285 | 0.0285 | 0.2087 | 3 |
| hsa04014 | Ras signaling pathway | 16/342 | 238/8541 | 0.0294 | 0.0294 | 0.2087 | 16 |
| hsa04540 | Gap junction | 8/342 | 92/8541 | 0.0306 | 0.0306 | 0.2087 | 8 |
| hsa05214 | Glioma | 7/342 | 76/8541 | 0.0319 | 0.0319 | 0.2087 | 7 |
| hsa04010 | MAPK signaling pathway | 19/342 | 300/8541 | 0.0321 | 0.0321 | 0.2087 | 19 |
| hsa04370 | VEGF signaling pathway | 6/342 | 60/8541 | 0.0321 | 0.0321 | 0.2087 | 6 |
| hsa05222 | Small cell lung cancer | 8/342 | 93/8541 | 0.0324 | 0.0324 | 0.2087 | 8 |
| hsa04360 | Axon guidance | 13/342 | 184/8541 | 0.0333 | 0.0333 | 0.2093 | 13 |
| hsa04213 | Longevity regulating pathway - multiple species | 6/342 | 62/8541 | 0.0369 | 0.0369 | 0.2273 | 6 |
| hsa05225 | Hepatocellular carcinoma | 12/342 | 170/8541 | 0.0401 | 0.0401 | 0.2412 | 12 |
| hsa03250 | Viral life cycle - HIV-1 | 6/342 | 64/8541 | 0.0422 | 0.0422 | 0.2439 | 6 |
| hsa05215 | Prostate cancer | 8/342 | 98/8541 | 0.0423 | 0.0423 | 0.2439 | 8 |
| hsa04666 | Fc gamma R-mediated phagocytosis | 8/342 | 99/8541 | 0.0445 | 0.0445 | 0.2462 | 8 |
| hsa05231 | Choline metabolism in cancer | 8/342 | 99/8541 | 0.0445 | 0.0445 | 0.2462 | 8 |
| hsa04310 | Wnt signaling pathway | 12/342 | 174/8541 | 0.0465 | 0.0465 | 0.2524 | 12 |

**Supplementary Table 2** List of differentially expressed proteins associated with key immune pathways

| Index | Control_1 | Control_2 | Control_3 | TSTTF_1 | TSTTF_2 | TSTTF_3 | p-value | significant | q-value |
| --- | --- | --- | --- | --- | --- | --- | --- | --- | --- |
| BTK | 17.26283 | 17.68142 | 17.83713 | 18.81647 | 18.64088 | 18.87183 | 0.00307871 | ** | 0.011545161 |
| CAP1 | 19.56655 | 19.72498 | 19.21745 | 20.57822 | 20.25146 | 20.81576 | 0.00922704 | ** | 0.018254 |
| CBL | 17.59324 | 17.74994 | 17.66501 | 16.58411 | 16.01119 | 16.59088 | 0.002965968 | ** | 0.011545161 |
| CD48 | 16.60667 | 16.36465 | 16.7489 | 18.24136 | 17.9562 | 17.73735 | 0.001581695 | ** | 0.010252215 |
| CD14 | 18.92012 | 18.80192 | 18.49728 | 16.85137 | 17.71168 | 16.52853 | 0.010344163 | * | 0.018254 |
| CD27 | 17.81968 | 17.79832 | 18.14773 | 18.68392 | 18.99079 | 19.11098 | 0.004088044 | ** | 0.013626814 |
| CD2AP | 18.67032 | 17.81863 | 18.1471 | 19.37463 | 19.30777 | 18.75167 | 0.042297664 | * | 0.0488049 |
| CD59 | 17.07222 | 17.29671 | 17.86479 | 18.55259 | 22.25014 | 20.40136 | 0.05215416 | not significant | 0.0579490 |
| CD5L | 20.38577 | 20.00714 | 19.70086 | 20.91029 | 21.42734 | 20.97778 | 0.013764999 | * | 0.01979408 |
| CDC37 | 18.79295 | 18.6658 | 18.7212 | 16.27744 | 17.72231 | 16.91472 | 0.013894289 | * | 0.0197940 |
| CSK | 19.81159 | 20.83501 | 19.63638 | 17.80283 | 18.56385 | 18.69696 | 0.020255914 | * | 0.025319892 |
| Index | Control_1 | Control_2 | Control_3 | TSTTF_1 | TSTTF_2 | TSTTF_3 | p-value | significant | q-value |
| DOK3 | 19.35421 | 19.515 | 19.8443 | 18.7163 | 18.56443 | 18.85478 | 0.006741072 | ** | 0.0182544 |
| FAS | 16.39123 | 16.90379 | 17.2245 | 18.31932 | 19.09315 | 18.20339 | 0.010094359 | * | 0.0182544 |
| FYB1 | 19.49747 | 19.31906 | 19.36254 | 20.20935 | 20.85312 | 20.4674 | 0.004566392 | ** | 0.013699175 |
| GRAP2 | 17.35137 | 17.58899 | 17.11992 | 18.30678 | 19.40026 | 18.67232 | 0.01451566 | * | 0.01979408 |
| GRB2 | 18.94646 | 18.30205 | 18.12041 | 16.75751 | 17.06105 | 17.33275 | 0.009479689 | ** | 0.0182544 |
| IGD | 16.97718 | 16.49811 | 17.21925 | 18.49022 | 18.23122 | 17.81109 | 0.011576199 | * | 0.0192936 |
| IGHM | 21.03821 | 21.40257 | 22.26988 | 22.44066 | 22.55367 | 22.34435 | 0.077170743 | not significant | 0.079831 |
| IGK | 21.89644 | 21.68103 | 21.82243 | 22.86492 | 22.80537 | 22.34397 | 0.007805578 | ** | 0.0182544 |
| IGKC | 27.99402 | 27.86962 | 27.82548 | 29.13843 | 28.44145 | 28.61202 | 0.018040793 | * | 0.02353146 |
| IGL1 | 26.55644 | 26.81609 | 27.1645 | 27.66464 | 27.64468 | 28.02489 | 0.012315696 | * | 0.0194458 |
| IGLL1 | 19.45352 | 19.40339 | 19.38453 | 22.88836 | 20.33416 | 20.84864 | 0.067433425 | not significant | 0.07225009 |
| LY6D | 18.95897 | 18.58418 | 18.95897 | 17.10799 | 17.48697 | 16.90776 | 0.001386044 | ** | 0.010252215 |
| LYRIC | 19.84492 | 19.82818 | 20.13135 | 18.47242 | 18.14824 | 19.05845 | 0.008382466 | ** | 0.0182544 |
| MARCS | 18.64495 | 19.12153 | 18.94714 | 17.14823 | 17.17348 | 17.66209 | 0.001925985 | ** | 0.010252215 |
| MAVS | 16.99893 | 16.57941 | 16.78763 | 17.72041 | 17.64271 | 17.64718 | 0.002050443 | ** | 0.010252215 |
| SHIP1 | 17.83587 | 17.61383 | 17.70151 | 16.47261 | 16.84834 | 16.70035 | 0.001194053 | ** | 0.010252215 |
| SLPI | 18.74052 | 18.93355 | 20.05195 | 17.90547 | 17.50248 | 17.72312 | 0.022691264 | * | 0.027229517 |
| SRC | 17.52581 | 18.33104 | 17.32624 | 18.9103 | 18.09185 | 18.97091 | 0.090092343 | not significant | 0.090092343 |
| ZA2G | 23.99187 | 24.03322 | 24.21426 | 23.20189 | 23.2693 | 23.24016 | 0.000288979 | *** | 0.008669361 |
